# HPV16 genetic variation provides evidence of positive natural selection driven by HLA class I

**DOI:** 10.1038/s41467-026-73531-0

**Published:** 2026-06-02

**Authors:** Chase W. Nelson, Sambit K. Mishra, Michael Dean, Colm Ohuigin, Robert D. Burk, Bin Zhu, Difei Wang, Laurie Burdett, Mathias Viard, Hyo Jung Lee, Aimee J. Koestler, Apurva Narechania, Zigui Chen, Nicolas Wentzensen, Mark Schiffman, Gary M. Clifford, Elizabeth Suh-Burgmann, Thomas Lorey, Mary Carrington, Meredith Yeager, Lisa Mirabello

**Affiliations:** 1 Division of Cancer Epidemiology and Genetics, National Cancer Institute, National Institutes of Health, Rockville, MD USA; 2 Cancer Genomics Research Laboratory, Frederick National Laboratory for Cancer Research, Rockville, MD USA; 3 Basic Science Program, Frederick National Laboratory for Cancer Research, National Cancer Institute, Frederick, MD USA; 4 Laboratory of Integrative Cancer Immunology, Center for Cancer Research, National Cancer Institute Bethesda, Bethesda, MD USA; 5 Departments of Pediatrics, Epidemiology and Population Health, Microbiology and Immunology, and Obstetrics & Gynecology and Women’s Health, Albert Einstein College of Medicine, New York, NY USA; 6 Institute for Comparative Genomics, American Museum of Natural History, New York, NY USA; 7 Department of Microbiology, The Chinese University of Hong Kong, Hong Kong SAR, China; 8 Early Detection, Prevention and Infections Branch, International Agency for Research on Cancer, Lyon, France; 9 Division of Research, Kaiser Permanente Northern California, Pleasanton, CA USA; 10 Regional Laboratory and Women’s Health Research Institute, Division of Research, Kaiser Permanente Northern California, Pleasanton, CA USA; 11 Ragon Institute of Massachusetts General Hospital, Massachusetts Institute of Technology and Harvard University, Cambridge, MA USA; 12 Department of Biology, Hood College, Frederick, MD USA

**Keywords:** Tumour virus infections, Molecular evolution

## Abstract

Human papillomavirus type 16 (HPV16) causes more cancer than any other virus. However, most HPV16 infections are controlled by the host’s immune system and it remains unclear how viral and host genetic variation contribute to infection outcomes. Here, we analyze 4704 HPV16 whole genomes to identify 56 viral codons putatively under positive natural selection to change their amino acids, with evidence including *d*_N_/*d*_S_ > 1, evolutionary convergence, and structural importance in the protein. We find that codons under positive selection disproportionately overlap known HPV16 immune epitopes recognized by cytotoxic T lymphocytes, particularly those restricted by the previously reported risk allele HLA-B*07:02 (odds ratio [OR] = 4.9; 95%CI = 2.1–10.3; *P*_Fisher_ = 0.00015), exemplified by position 10 of the E6 oncoprotein. Positively selected codons also disproportionately overlap 158 nucleotide sites at which the evolutionary sub/lineages of HPV16 have diverged (OR = 19.1; 95%CI = 10.5–34.7; *P*_Fisher_ < 2.2×10^−16^), and show more rare variation in cervical precancers/cancers than controls (benign or cleared HPV16 infections) in the E1 protein (OR = 9.34, 95%CI = 1.4–402.5; *P*_Fisher_ = 0.0084). Our results suggest that a small subset of HPV16 variants can improve viral persistence through escape of HLA-related immune recognition. The interaction of HPV16 and HLA variation may help to explain how similar or identical viral isolates can have such disparate infection outcomes.

## Abstract (web summary)

Human papillomavirus (HPV) causes more cancer than any other virus, yet for unknown reasons, HPV experiences differential success infecting and persisting in individuals. To investigate how human and virus genetic variation contribute to these wide differences in outcomes, Nelson et al. analyzed HPV16 whole genomes and find that a small subset of HPV16 mutations can improve viral fitness and influence infection outcomes, with escape of T cell immune recognition as a likely selective pressure.

## Introduction

Persistent infection with one of ~13 types of oncogenic human papillomavirus (HPV) is necessary for the development of cervical cancer, which—despite screening and vaccination efforts—remains the fourth most common cancer in women worldwide^1^. For unknown reasons, HPV has differential success infecting and persisting in different individuals. Nearly all HPV infections are cleared or immune-controlled without progression to precancer or cancer^2^. Nevertheless, oncogenic HPVs are so prevalent that they cause ~661,000 new cervical cancers (~348,000 cervical cancer deaths) each year^3^, of which ~61% are due to the unique carcinogenicity of just one type: HPV16^4,5^.

The HPV16 genome comprises ~7900 bp and eight main open reading frames (ORFs): E6, E7, E1, E2, E4, E5, L2, and L1 (5′ to 3′), of which E6 and E7 encode the oncoproteins. Variation in the HPV16 genome can be used to classify isolates into four main evolutionary lineages: A, B, C, and D. Studies suggest that these lineages shared a most recent common ancestor ~500 thousand years ago, when lineage A diverged from B/C/D^6,7^. Viral diversification has since given rise to ~16 HPV16 sublineages (A1–4, B1–4, C1–4, and D1–4) that are distinguished by variants at ~150 “sub/lineage-defining” nucleotide positions^8^ (Box 1 concept key), with the most diverged isolates differing by ~2% of the genome. Risk of cervical precancer/cancer varies depending on which sublineage infects an individual: relative to A1/A2, risk is lower for B1 infections but higher for A4, C1, D2, and D3 infections, with the greatest risk observed for D2 (odds ratio [OR] = 137 for adenocarcinoma)^9^. At a finer scale of variability, HPV16 SNPs and E7 genetic conservation have also been linked to different risks of precancer/cancer^10,11^. It is important to understand how infection outcomes are mediated by variation in the HPV16 genome and how this variation interacts with the human host. For instance, variation in the E6 or E7 oncoproteins could influence their biological function and/or immune recognition.

The genetic diversity of HPV16 has been primarily shaped by random genetic drift^12^ and no compelling instances of recombination have been reported^8^. However, just like the genome of its human host, the HPV16 genome has also been subject to natural selection^7,13–15^. Natural selection can be categorized into two main types: (1) purifying selection, which acts to eliminate disadvantageous mutations, and (2) positive selection, which acts to favor advantageous mutations^16^. Purifying selection promotes conservation of functionally important genome positions that are said to be constrained (intolerant of change), whereas positive selection promotes functionally important genetic changes. In protein-coding regions, nonsynonymous mutations tend to have more functional impact than synonymous mutations. Thus, purifying selection tends to manifest as *d*_N_ < *d*_S_—a decreased rate of nonsynonymous divergence (*d*_N_) relative to synonymous divergence (*d*_S_)—whereas positive selection tends to manifest as *d*_N_ > *d*_S_. Positive selection’s contribution to HPV16 variation and evolution remains unclear, and it can be difficult to distinguish from random genetic drift of neutral changes^16^.

Immune control of viral infection is mediated by CD8+ cytotoxic T lymphocytes (CTL)^17^ that recognize epitopes—viral peptide fragments ~7–11 amino acids in length that are presented on the infected cell’s surface by major histocompatibility complex (MHC) class I molecules^18^ (Box 1 concept key). Several lines of evidence suggest that MHC is important for HPV control. One of HPV’s eight proteins, E5, disrupts MHC class I expression and epitope presentation to CTL^19^. HPV-associated cervical disease is exacerbated in immunosuppressed individuals^20^. Somatic mutation or loss of human leukocyte antigen (HLA) genes, which encode MHC molecules, is frequently observed in cervical precancers and cancers^21–24^. Cervical cancer heritability^25,26^ has been reported as high as 34%^27,28^, and the HLA region is the most consistently identified locus in genome-wide (GWAS)^29–33^ and targeted^34–36^ genetic association studies of cervical precancer/cancer—including associations that differ by HPV type or by variant within type^32,34–36^. For HPV16, GWAS suggest that risk of cervical disease is affected by HLA class I alleles B*07:02 (risk) and B*15:01 (protective)^32^, with B*07:02 being among the most high-frequency alleles at the HLA-B locus^37^.

We hypothesized that, just as pathogens have exerted detectable positive selection on the human genome’s HLA genes^38^, human immunity has exerted detectable positive selection on the HPV16 genome^39^ by favoring amino acid changes that provide a selective advantage to the virus. For example, escaping MHC binding or CTL detection would be beneficial for the virus and select for viral variants within epitopes, potentially leading to *d*_N_ > *d*_S_ at specific codons^40^. Direct selection to increase viral incidence or persistence could inadvertently affect carcinogenicity^8^. To test for evidence of positive selection, we used multiple approaches to analyze a dataset of 4704 HPV16 whole genomes. We identified 56 codons putatively under positive selection that had high rates of amino acid change and may impact both cancer risk prognosis and immunotherapies. We documented the molecular, evolutionary, and immunological properties of all HPV16 codons, and provided Supplementary Data 1 as a community resource for further research.

Box 1 Concept key

**Table:** 

Term	Definition
Convergence	Repeated evolution of the same molecular changes in different parts of the viral evolutionary tree; may involve recurrence (e.g., C→T occurs twice) or reversion to an ancestral state (e.g., C→T followed by T→C).
Cytotoxic T lymphocytes (CTL)	CD8 + T cells that recognize viral peptide fragments (epitopes) presented by MHC class I molecules on the surface of infected cells; control infection through cell killing or cytokine-mediated suppression.
Epitope	A short segment of a viral protein that is bound by MHC molecules, presented on the cell surface, and recognized by the immune system; most commonly 9-mers for CTL.
HLA/MHC class I	Human leukocyte antigen (HLA) class I genes encode major histocompatibility complex (MHC) class I molecules; variations in the HLA genes determine the specificity of the viral epitopes their MHC products can bind and present to CTL.
Sub/lineage-defining sites	Genome positions at which HPV16 evolutionary sub/lineages (sublineages or lineages) have evolved nucleotides differences.
Physicochemical amino acid distance	A measure of the difference in charge, polarity, and/or volume of two amino acids, on a spectrum of conservative (very similar) to moderate (somewhat different) to radical (very different).
Positive selection	Evolutionary pressure favoring an increase in the frequency of mutations that confer a fitness advantage.
Centrality/second order degree centrality (SODC)	An amino acid residue’s total number of distinct interactions that are two neighbors away in the protein’s three-dimensional structure; a measure of the structural and functional importance of the residue’s position.
STOP-proximal codons	Codons that can mutate to a STOP codon via a single nucleotide change: AAA, AAG, AGA, CAA, CAG, CGA, GAA, GAG, GGA, TAC, TAT, TCA, TCG, TGC, TGG, TGT, TTA, or TTG.

## Results

### Sequence data

We evaluated 4704 HPV16 whole genome sequences^9,10^ obtained from HPV16-positive cervical samples in three studies: 1342 internationally collected by the International Agency for Research on Cancer (IARC); 2773 from the Kaiser Permanente Northern California (KPNC)-NCI HPV Persistence and Progression (PaP) cohort; and 589 from the NCI Study to Understand Cervical Cancer Early Endpoints and Determinants (SUCCEED) (summarized in Table 1). All HPV16 samples underwent identical sequencing, processing, masking, and quality control filtering (see Methods; Supplementary Information).

**Table 1:** Characteristics of 4704 HPV16 whole genome sequences from three studies

Study^a^	Infection outcome^b^	Sub/lineage							Total
		A1/A2 N (col %)	A3 N (col %)	A4 N (col %)	B N (col %)	C N (col %)	D2/D3 N (col %)	Other/NA N (col %)	
PaP	Control	718 (32.0)	5 (33.3)	14 (25.9)	30 (45.5)	11 (18.0)	33 (16.9)	39 (28.1)	850
	CIN2	671 (29.9)	5 (33.3)	13 (24.1)	16 (24.2)	22 (36.1)	30 (15.4)	25 (18.0)	782
	CIN3/precancer	805 (35.9)	5 (33.3)	24 (44.4)	17 (25.8)	26 (42.6)	97 (49.7)	73 (52.5)	1047
	Cancer	49 (2.2)	0	3 (5.6)	3 (4.5)	2 (3.3)	35 (17.9)	2 (1.4)	94
	Total	2243 (100)	15 (100)	54 (100)	66 (100)	61 (100)	195 (100)	139 (100)	2773
SUCCEED	Total^c^	522	1	3	6	17	36	4	589
IARC	Total^d^	794	88	163	64	123	97	13	1342
All studies	No. (row %)	3559 (75.7)	104 (2.2)	220 (4.7)	136 (2.9)	201 (4.3)	328 (7.0)	156 (3.3)	4704 (100)

^a^PaP = Kaiser Permanente Northern California (KPNC)-NCI HPV Persistence and Progression cohort; SUCCEED = NCI Study to Understand Cervical Cancer Early Endpoints and Determinants; IARC = International Agency for Research on Cancer.

^b^PaP was the largest study with controls, precancers, and cancers; Controls = cervical intraepithelial neoplasia (CIN) grade 1 or lower ( ≤ CIN1); CIN2 = CIN grade 2; CIN3 = CIN grade 3.

^c^SUCCEED includes 149 < CIN2, 134 CIN2, 182 CIN3 precancer, and 57 cancer samples.

^d^IARC includes 252 non-cancer, 535 cancer, and 7 unknown status samples.

### HPV16 sub/lineage-defining sites are enriched for convergent mutations

**Fig. 1:**
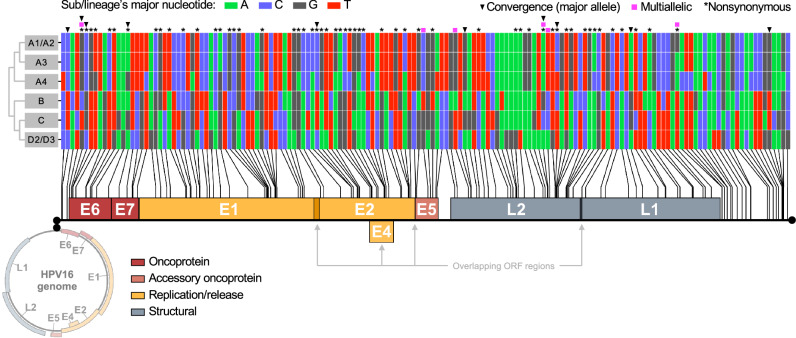
Sub/lineage-defining sites in the HPV16 genome. The circular HPV16 genome has been linearized to display 158 sites at which nucleotide differences were nearly fixed between six phylogenetically independent sub/lineages (see Supplementary Data 2 and source data for the list of sites displayed). Tile color^92^ denotes each sub/lineage’s major (most common) nucleotide (green = A, blue = C, black = G, red = T). ORF color denotes its protein’s functional role (dark red = oncoprotein, salmon = accessory oncoprotein, yellow = replication/release, grey = structural). E8, a short spliced region overlapping E1, is not shown. Convergence = sites at which the same major alleles have evolved multiple times via recurrent or revertant mutation, indicated with inverted triangles. Multiallelic = sites with more than two major alleles, indicated with magenta squares (multiallelic minor alleles are not indicated). Nonsynonymous = sites at which the major nucleotide difference implies an amino acid change, indicated with asterisks (annotated only with respect to E2 in the E2/E4 overlapping region).

We first inferred the major genetic changes involved in HPV16 evolution by identifying genome positions at which six phylogenetically independent, epidemiologically relevant viral lineages or sublineages differ (A1/A2, A3, A4, B, C, and D2/D3; hereafter “sub/lineages”) (Supplementary Fig. S1). This analysis yielded 158 sub/lineage-defining sites (Fig. 1 and Supplementary Data 2). These sites were more likely than the remainder of the genome to exhibit patterns of molecular convergence (Box 1 concept key): 99% (all but one) exhibited convergence, compared to only 39% (911 of 2335) of the remaining HPV16 polymorphic sites (Supplementary Fig. S2 and Supplementary Data 3). This pattern held even when limiting to only multiallelic sites (98.6% vs. 67.7% with convergence). At ten sub/lineage-defining sites, convergent changes had risen in frequency to become the sub/lineage’s defining nucleotide (Fig. 1). Thus, sub/lineage-defining sites have undergone more convergent evolution than the rest of the HPV16 genome and may represent hotspots of evolutionary change.

### Sub/lineage-defining codons have elevated diversity and relaxed constraint

**Fig. 2:**
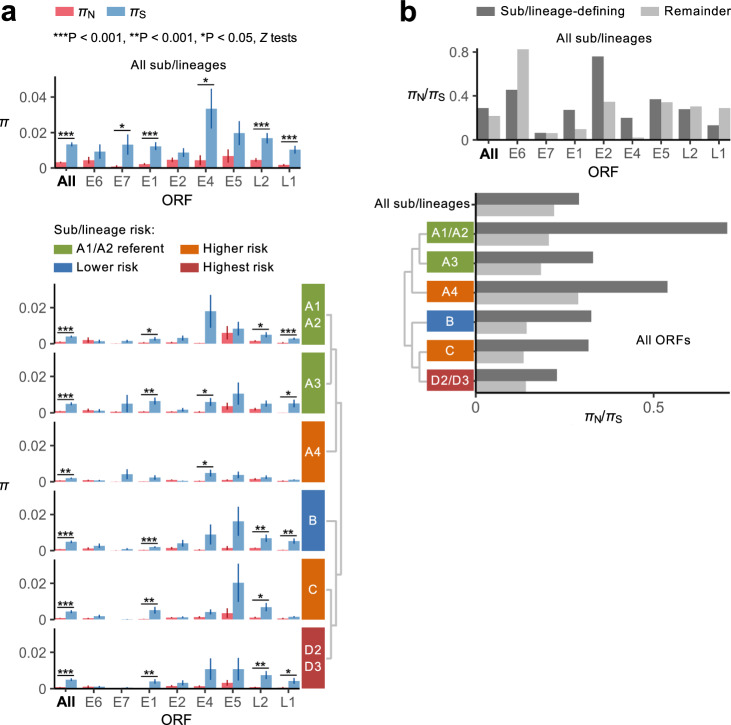
Nucleotide diversity of HPV16 protein-coding ORFs. **a** Nonsynonymous (*π*_N_; red) and synonymous (*π*_S_; blue) nucleotide diversities were estimated among all samples of all sub/lineages (top; includes both between- and within-sub/lineage comparisons) and among all samples within each sub/lineage (bottom; within-sub/lineage comparisons only). Error bars show standard errors estimated by bootstrapping codon units (alignment columns) with 1000 replicates. *P* values refer to Z tests of *π*_N_ = *π*_S_ (two-sided) with no correction for multiple comparisons. Numbers of sequences for each sub/lineage were: All = 4704, A1/A2 = 3559, A3 = 104, A4 = 220, B = 136, C = 201, and D2/D3 = 328. Numbers of codons (ORF lengths excluding STOPs) were: All = 2422 (whole coding genome), E6 = 151, E7 = 98, E1 = 649, E2 = 365, E4 = 86 (3′-spliced portion), E5 = 83, L2 = 473, and L1 = 505 codons. Sub/lineages are colored with respect to epidemiologically determined risk^9^ (green = A1/A2 referent; blue = lower risk; orange = higher risk; dark red = highest risk). The grey tree shows phylogenetic topology (evolutionary relationships); branch lengths are not to scale. **b** *π*_N_/*π*_S_ ratios at sub/lineage-defining codons (dark grey) compared to all other codons (“remainder”; light grey).

To characterize genetic diversity in HPV16’s protein-coding ORFs (Supplementary Table S1), we estimated viral nucleotide diversity (*π*) among all pairs of isolates. Diversity was lower at nonsynonymous (*π*_N_) than synonymous (*π*_S_) sites in the full protein-coding genome, with *π*_N_/*π*_S_ = 0.24 (Fig. 2a, top). All significant *π*_N_/*π*_S_ ratios were <1 for all sub/lineages and ORFs. HPV16 isolates had an average of 17 nonsynonymous differences (0.32% of 5357 nonsynonymous sites) and 22 synonymous differences (1.34% of 1656 synonymous sites) between pairs of samples in our dataset, which is ~76% fewer amino acid changes than expected by random drift of neutral mutations. These results support previous observations that the average HPV16 codon is under purifying selection^12,14,41^.

Of 2418 codons analyzed, 137 (5.7%) contained sub/lineage-defining sites. These sub/lineage-defining codons had significantly higher *π*_N_/*π*_S_ (0.29) than the remainder of codons (0.21) across all sub/lineages (*P*_Wilcoxon_ = 0.031) (Fig. 2b). Thus, the codons at which sub/lineages differed also had elevated *π*_N_/*π*_S_ and tended to be the most variable positions within each sub/lineage.

Comparing the HPV16 sub/lineages, the highest nonsynonymous diversity was observed for the globally most common A1/A2 sub/lineage (*π*_N_ = 0.00105; average of 5.6 amino acid differences between isolates), while the lowest nonsynonymous diversity was observed for the rarer A4 sublineage, which is most dominant in East Asia^42,43^ (*π*_N_ = 0.00067; average of 3.6 amino acid differences between isolates). The more carcinogenic but globally less prevalent D2/D3 sub/lineage was the most constrained (i.e., lowest *π*_N_/*π*_S_) at the whole-genome level (*π*_N_/*π*_S_ = 0.16) (Fig. 2a, bottom). Comparing the ORFs, the most constrained was the critical E7 oncoprotein (*π*_N_/*π*_S_ = 0.06), followed by E4 (*π*_N_/*π*_S_ = 0.13) (Fig. 2a, top). E5 had the highest *π*_N_ (0.007) but an intermediate level of constraint (*π*_N_/*π*_S_ = 0.34). E2 showed the least constraint, but with a point estimate still suggesting purifying selection predominates (*π*_N_/*π*_S_ = 0.53). Thus, while the HPV16 genome was dominated by purifying selection, signals of selection differed by sub/lineage, ORF, and genomic region, and elevated nonsynonymous diversity was observed at sub/lineage-defining sites.

### Structurally important amino acids show heightened constraint

The overlap between nonsynonymous diversity and sub/lineage-defining sites has competing explanations: positive selection (functional importance) or random genetic drift of neutral changes (functional unimportance). To distinguish these hypotheses, we first predicted the monomeric structure of each HPV16 protein to determine each amino acid residue’s structural importance as estimated by its second order degree centrality (SODC; Box 1 concept key) (Supplementary Fig. S3). This allowed us to identify functionally important positions within the protein structure using a method that is independent of *π*_N_/*π*_S_.

**Fig. 3:**
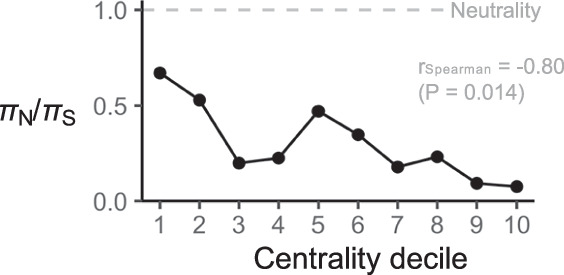
Inverse correlation between amino acid residue structural centrality and codon constraint. Monomeric protein structures were predicted for each viral protein using AlphaFold2^86^. Structural centrality was measured using second order degree centrality (SODC). Constraint was estimated using *π*_N_/*π*_S_ (all samples, all sub/lineages) and displayed as a function of SODC deciles, limiting to high-confidence residues with predicted Local Distance Difference Test (pLDDT) values of >70. The *P* value refers to a Spearman’s rank correlation coefficient (two-sided; centrality decile unit).

Amino acid structural centrality and *π*_N_/*π*_S_ were inversely correlated: more important residues were more constrained by purifying selection as indicated by less nonsynonymous diversity (*r* = −0.80; *P*_Spearman_ = 0.0137), with the lowest and highest SODC deciles having the highest (0.67) and lowest (0.08) *π*_N_/*π*_S_, respectively (Fig. 3). Nevertheless, exceptional positions having both high *π*_N_/*π*_S_ and high SODC were observed (Supplementary Fig. S4 and Supplementary Data 1), suggesting that positive selection may have favored certain amino acid changes predicted to be functionally impactful (next section).

### Positively selected codons are associated with sub/lineage-defining sites, convergence, and infection outcome

**Fig. 4:**
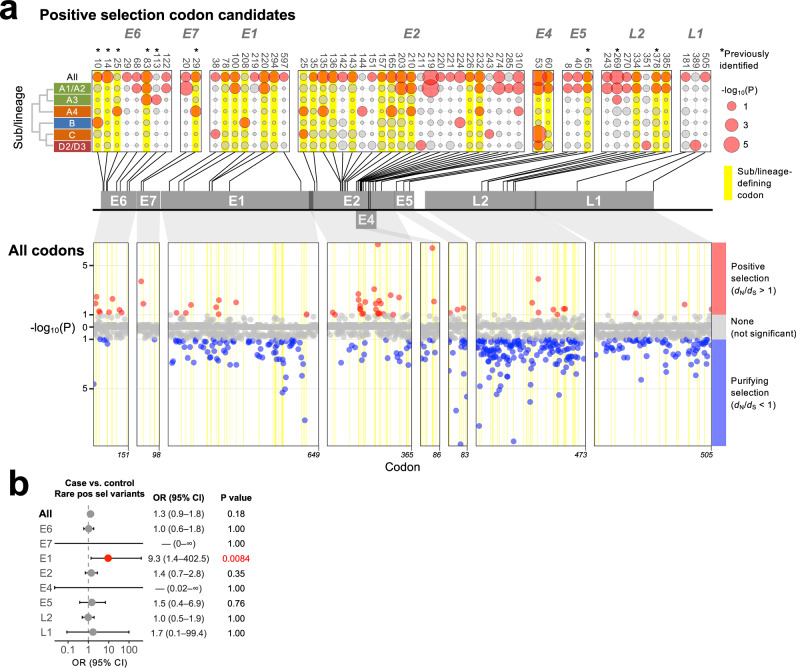
Natural selection and cervical precancer/cancer associations in HPV16 protein-coding ORFs. **a** Selection analyses for the protein-coding HPV16 genome. A *d*_N_/*d*_S_ analysis was performed for each ORF using both HyPhy-FEL and -MEME, where *d*_N_/*d*_S_ > 1 (red dots) indicates positive selection and *d*_N_/*d*_S_ < 1 (blue dots) indicates purifying selection. Likelihood ratio tests (LRTs) were two-sided for FEL and one-sided for MEME, each with a significance threshold of *P*_LRT_ < 0.1 and no correction for multiple comparisons. Only internal tree branches were considered (see Methods). Each selection analysis was run five times with distinct but statistically indistinguishable maximum likelihood trees^78^ and the median *P* value was retained for each codon (minimum of FEL or MEME). Top: codons with evidence of positive selection (*P*_LRT_ < 0.1) in at least one sub/lineage. Black lines indicate genome position; bubble size depicts significance of the likelihood ratio test (salmon = significant, grey = non-significant); yellow highlight indicates a sub/lineage-defining codon; “Previously identified” indicates codons with published evidence of positive selection^7,13–15^ (Supplementary Table S2). Middle: the circular HPV16 genome has been linearized for ease of reference. Bottom: selection results for all codons, where grey is statistically non-significant (*P*_LRT_ ≥ 0.1), and red (positive selection) or blue (purifying selection) are significant (*P*_LRT_ < 0.1), displaying the minimum *P* value observed across sub/lineages. **b** Associations between cervical case/control status and the presence of rare (frequency <5% of samples) amino acid variants at positively selected codons. Results are based on 854 cases and 718 controls infected with A1/A2 sub/lineage virus. *P* values refer to Fisher’s exact tests (two-sided) on sample counts. Dots show odds ratios (red = *P*_Fisher_ < 0.05); error bars show 95% confidence intervals. The *x*-axis uses a log_10_ scale. Point estimates were not possible in E7 or E4 given insufficient variation (only two positively selected codons each).

To test for positive selection (*d*_N_ > *d*_S_) at specific HPV16 codons, we used phylogenetic trees to infer nonsynonymous (*d*_N_) and synonymous (*d*_S_) substitution rates along evolutionary lines of descent, i.e., branches (Fig. 4a). These trees allowed us to determine the evolutionary relationships among isolates regardless of geographic origin. Only internal tree branches were examined (Supplementary Fig. S5), thereby limiting to viral mutations that have undergone at least one round of successful transmission^44^ and are likely “circulating” in the population (see “Methods”).

For 2410 HPV16 codons at which *d*_N_/*d*_S_ was estimable, we detected evidence of positive selection at 56 codons (Fig. 4a, top) and purifying selection at 349 codons (Fig. 4a, bottom). The positively selected codons included nine that have been identified in previous studies^7,13–15^ (E6:10, 14, 25, 83, 113; E7:29; E5:65; L2:269, 378; Supplementary Table S2) and 47 new candidates. Information about each of the 56 codons with evidence for positive selection is given in Supplementary Data 4.

Positively selected codons were enriched for sub/lineage-defining sites (OR = 19.1; 95% CI = 10.5–34.7; P_Fisher_ < 2.2 × 10^−16^) (Fig. 4a, top). All but three of the positively selected codons also had *π*_N_ > *π*_S_ in our full sequence dataset (Supplementary Data 1), indicating relatively high global levels of amino acid diversity at these positions. Consistent with positive selection, these codons were also enriched for convergent changes (87.5%) compared to the remaining 1492 polymorphic codons (50.5%; OR = 6.8; 95% CI = 3.1–18.0; *P*_Fisher_ = 1.31 × 10^−8^).

To determine whether positively selected variation was relevant to oncogenesis, we compared 718 controls (less than cervical intraepithelial neoplasia [CIN] grade 2 [<CIN2]) and 854 cervical precancer/cancer cases (CIN grade 3 and cancer [CIN3+]) with A1/A2 sub/lineage virus in the PaP prospective cohort study (Table 1). No individual amino acid position showed a significant case vs. control difference after accounting for multiple comparisons. However, considering positively selected codons collectively, cases were more likely than controls to have rare (frequency <5%) nonsynonymous variants in E1 (OR = 9.34, 95% CI = 1.4–402.5; *P*_Fisher_ = 0.0084) (Fig. 4b), suggesting amino acid changes at these positions were associated with viral persistence or progression to cancer. Specifically, cases had more rare variation than controls at E1 positions 38 (N-terminal domain), 100 (nuclear export signal), and 208 and 294 (DNA-binding domain; UniProt ID: P03114) (Supplementary Fig. S6 and Supplementary Data 5). These E1 variants do not cluster in a single viral subclade or haplotype, but instead arose independently several times over the course of viral evolution (Supplementary Fig. S6).

To explore the possible functional roles of these E1 positions, we superimposed our AlphaFold2-predicted monomeric structure of the HPV16 E1 protein onto the experimentally determined hexameric E1–DNA complex from bovine papillomavirus (PDB ID: 7APD^45^). Positions 208, 219, and 220 were located in the proximity of the DNA molecule, suggesting roles in DNA binding and unwinding (Supplementary Fig. S7). Of these, position 208 was closest to the DNA, and its variants (L208Q, L208V) were predicted to destabilize the E1 protein structure (ΔΔG < −1.0, DUET^46^ and DynaMut2^47^; Supplementary Data 5).

### Known CTL epitopes are enriched for positively selected sites

**Fig. 5:**
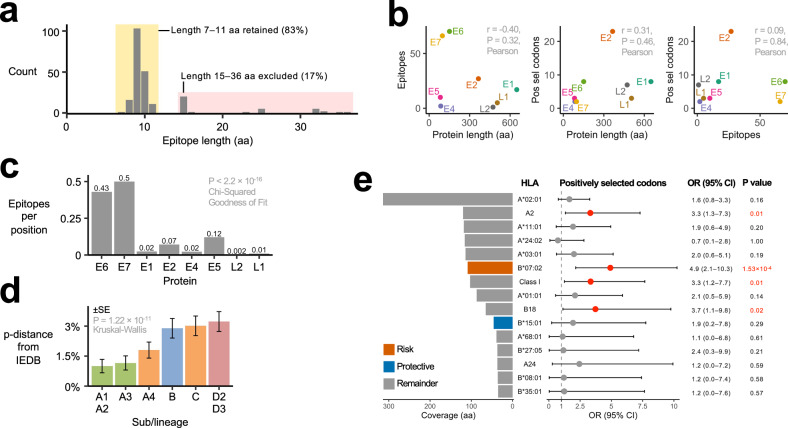
HPV16 CTL epitopes from the Immune Epitope Database. **a** Length distribution of HPV16 CTL epitopes in the IEDB. We retained only the 181 unique epitopes that were of length 7–11 amino acids (yellow box) and excluded the remainder (pink box). **b** Relationships between number of CTL epitopes, positively selected codons, and protein length across the ORFs/proteins (dot colors). *P* values refer to Pearson’s correlation coefficients (two-sided; ORF unit). **c** Number of IEDB CTL epitope records mapped per position for each viral protein. **d** Mean amino acid p-distance between the IEDB epitope sequences and the protein variants of each sub/lineage (colors as in Fig. 2). Error bars show standard errors of mean p-distance. The *P* value refers to a Kruskal–Wallis test of differences in p-distance from the 181 epitopes across sub/lineages. **e** Associations (degree of overlap) between positively selected codons and CTL epitopes as a function of the HLA allele label provided by the IEDB (either a specific allele or a broader category label). Left: number of distinct amino acid positions occurring in CTL epitopes restricted by each IEDB HLA label (ordered by descending count); only HLA labels with epitopes covering ≥35 residues are shown. Color indicates status as a known risk (orange) or protective (blue) allele for HPV16-related cervical precancer/cancer^32^. Right: Fisher’s exact tests (two-sided) on amino acid counts for overlap between the CTL epitopes of each HLA allele or serotype and positively selected codons (genome-wide). Dots show odds ratios (red = *P*_Fisher_ < 0.05); error bars show 95% confidence intervals. Numbers of amino acids (epitopes) per HLA allele were: A*02:01 = 314 (66), A2 = 120 (21), A*11:01 = 118 (18), A*24:02 = 116 (16), A*03:01 = 114 (13), B*07:02 = 109 (13), Class I = 103 (16), A*01:01 = 87 (12), B18 = 65 (8), B*15:01 = 46 (5), A*68:01 = 39 (4), B*27:05 = 37 (5), A24 = 37 (5), B*08:01 = 36 (5), B*35:01 = 35 (4). IEDB = Immune Epitope Database.

One selective pressure favoring viral amino acid changes is host immunity. To explore associations between positively selected codons and experimentally verified immune targets in the HPV16 genome, we obtained all 181 unique HPV16 human CTL epitopes of length 7–11 amino acids from the Immune Epitope Database^48^ (IEDB), limiting to those with a positive T cell or MHC ligand assay (see “Methods”) (Fig. 5a and Supplementary Data 6 and 7).

HPV16 CTL epitopes were enriched for positively selected codons (OR = 2.9; *P*_Fisher_ = 0.000105). There was no correlation between a protein’s length, number of epitopes, or number of positively selected codons (Fig. 5b), and the distributions of epitopes and positively selected codons differed significantly across ORFs (*P*_*χ*²-GOF_ = 6.70 × 10^−11^), suggesting that the observed enrichment was not due to these potentially confounding variables. Unbiased comparisons among proteins or among sub/lineages were not possible because the epitopes in IEDB showed ascertainment bias favoring the E6 and E7 oncoproteins (Fig. 5c and Supplementary Fig. S8) and the most prevalent sub/lineage, A1/A2 (Fig. 5d).

To determine whether the association between CTL epitopes and positive selection in HPV16 was driven by specific HLA alleles, we categorized each epitope by its restricting HLA label as provided by the IEDB, which was either an individual allele (e.g., B*07:02) or a broader grouping (e.g., B7). For individual HLA alleles, enrichment for positively selected codons was observed solely for the known risk allele B*07:02 (OR = 4.9; 95% CI = 2.1–10.3; *P*_Fisher_ = 0.000153; Fig. 5e): 5 of the 13 B*07:02 epitopes contained 10 positively selected codons in E6 and E2 (Table 2). For broader HLA labels, enrichment was independently observed for B7, which includes B*07:02 (OR = 10.8; 1.1–55.7; *P*_Fisher_ = 0.0214): B7 had one epitope, which contained two positively selected codons in E6 (Table 2 and Supplementary Data 8). In contrast, no association was observed for the known protective allele B*15:01 (Fig. 5e), which had five epitopes.

**Table 2:** HLA-B*07:02-restricted CTL epitopes containing positively selected codons in HPV16

Epitope^a^	IEDB record	HLA allele	Positively selected codon(s)	References^c^
RPRKLPQLC	2157390	B*07:02	E6:10,14	Santegoets et al., 2023^103^
RPRKLPQLCT	911841	B*07:02	E6:10,14	Kristensen et al., 2024^104^; Krishna et al., 2018^105^
QERPRKLPQL	113122	B7^b^	E6:10,14	Piersma et al., 2008^106^
CPEEKQRHL	110197	B*07:02	E6:113	Kristensen et al., 2024^104^; Krishna et al., 2018^105^
SPEIIRQHL	911857	B*07:02	E2:210,211	Kristensen et al., 2024^104^; Krishna et al., 2018^105^
HPAATHTKAV	911753	B*07:02	E2:219,220,221,224,226	Bhatt et al., 2020^107^; Kristensen et al., 2024^104^; Krishna et al., 2018^105^

^a^Positively selected positions are underlined.

^b^B7 is shown for completeness but was not included in the B*07:02 association test.

^c^References provided by the Immune Epitope Database (IEDB).

### A functionally important amino acid in the E6 oncoprotein has strong evidence of positive selection

Position 10 of the E6 oncoprotein (E6:10) is encoded by a sub/lineage-defining codon that is under positive selection and falls within at least two B*07:02 epitopes (Fig. 4a and Table 2 and 3). This position has intermolecular contacts with both p53 (Gln104 and Arg110) and E6AP (Gly9)^49^ and has previously been associated with cervical pathology^50–52^, the stability of the E6-E6AP-p53 complex^52^, and positive selection^7,13–15^.

**Table 3:** Five sub/lineage-defining positions with moderate/radical amino acid changes at structurally important residues

ORF	Codon (genome positions)	Lineage-defining site (type)^a^	Centrality (SODC)^b^	HPV16REF^c^ codon (aa)	Radical (interm) aa pairs	*d*_N_/*d*_S_ *P* value^d^	HLA restriction^e^ (epitope count)	Divergence from IEDB epitope^f^
E6	10 (131–133)	132 (NSYN)	Interm (26)	AGA (R)	G/I, G/R (I/R, I/T, R/T)	0.050	Class I (1), A*11:01 (1), B*07:02 (2), B7 (1)	0.09
	78 (335–337)	335 (NSYN)	Interm (23)	CAT (H)	N/Y (H/Y)	0.176	Class I (1), A*01:01 (2), A*02:01 (1), A*11:01 (1), A*24:02 (1), A24 (1), B*27:05 (1), B*40:01 (1), B18 (1)	0.15
E2	135 (3158–3160)	3159 (NSYN)	Interm (27)	ACA (T)	— (K/T)	0.002	—	—
	136 (3161–3163)	3161 (NSYN)	Interm (25)	CAT (H)	C/N, N/Y (C/H, C/Y, H/Y)	0.006	—	—
L1	76 (5864–5866)	5864 (NSYN)	Interm (30)	CAT (H)	— (H/Y)	0.336	—	—

^a^NSYN = nonsynonymous.

^b^SODC = second order degree centrality as determined by AlphaFold2; only residues with high-confidence predictions (pLDDT>70); Interm = intermediate centrality (middle 40%; SODC 20–32).

^c^HPV16REF (A1 sublineage) from PaVE; aa = amino acid.

^d^HyPhy likelihood ratio test; minimum *P* value of FEL and MEME methods across sub/lineages.

^e^Immune Epitope Database (IEDB) allele or group labels.

^f^Mean amino acid p-distance of our HPV16 samples from the IEDB epitope sequence(s).

**Fig. 6:**
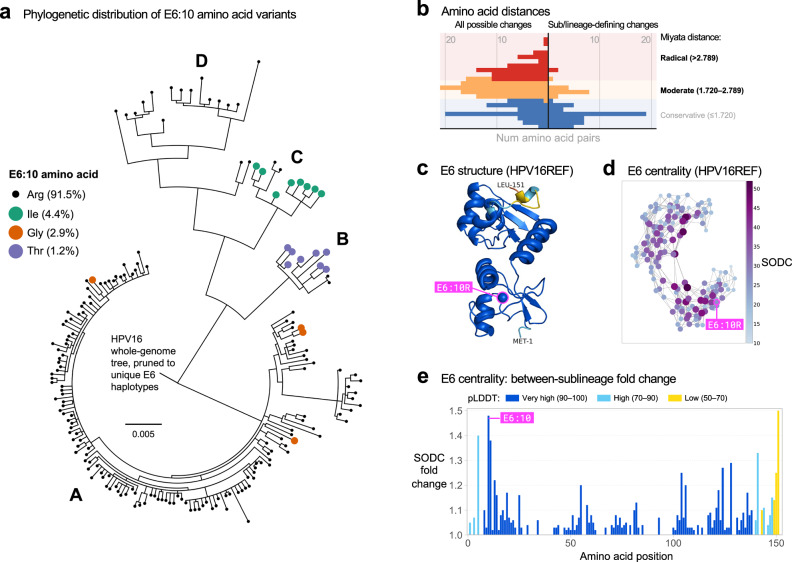
Evolution, amino acid variation, and structural centrality of E6 position 10. **a** Amino acid variants at E6:10 are overlaid on the best-found maximum likelihood phylogenetic tree inferred from HPV16 whole genomes by RAxML-NG. Tree leaves correspond to unique E6 nucleotide haplotypes, with duplicates pruned using HyPhy. Colored circles denote the amino acid encoded by each haplotype at E6:10 (black = Arg; green = Ile; orange = Gly; purple = Thr). Bold uppercase letters indicate HPV16 lineages. **b** Physicochemical distances between all possible amino acid pairs (left) and sub/lineage-defining differences (right) using the values of Miyata et al.^93^, categorized as radical (>2.89, red), intermediate (1.720–2.789, orange), or conservative (≤1.720, blue). Pairs that require multiple nucleotide changes, which are disproportionately non-conservative, were included. **c** Predicted structure of the monomeric E6 protein; color denotes structural confidence (pLDDT = predicted local distance difference test) as in (**e**), and E6:10 is indicated in magenta. **d** E6 network graph; node size and color denote second order degree centrality (SODC; darker purple = higher centrality). **e** Maximum fold change (max/min; *y* axis) of SODC values observed at all E6 protein positions (*x* axis) between variants encoded by HPV16 sublineage reference sequences (PaVE^108^). Color denotes structural confidence based on pLDDT values: blue = very high (90–100); light blue = high (70–90); and yellow = low (50–70).

Four amino acid variants were present at E6:10 in our dataset: Arg, Ile, Gly, and Thr (Fig. 6a). Lys has also been reported^52^. At the sub/lineage-defining level, two changes at E6:10 were physicochemically moderate (Box 1 concept key): Arg→Ile and Arg→Thr (Fig. 6a, b). At lower frequencies, physicochemically radical changes and molecular convergence were also observed, including three independent Arg→Gly substitutions (Fig. 6a and Supplementary Fig. S9). This level of moderate/radical amino acid variation was surprising given E6:10 had intermediate/high network centrality in the predicted protein structure (Fig. 6c, d), a property typically associated with strong evolutionary constraint. Furthermore, the network centrality of this position varied substantially between variants, being ~1.5-fold higher in the C lineage (SODC = 34; high) than in the D2/D3 sub/lineage (SODC = 23; intermediate) (Fig. 6e). At the nucleotide level, the E6:10 codon (genome positions 131–133) had *π*_N_/*π*_S_ = 28.2 among isolates and *d*_N_/*d*_S_ = 4.1 along internal phylogenetic branches (Supplementary Data 1). Finally, the three Arg→Gly convergences each created a potentially hypermutable CpG site, representing positive selection of a radical amino acid change in direct opposition to known mutational biases^53,54^.

Briefly, other sub/lineage-defining positions also evolved changes that might be unexpected under neutral genetic drift. Five had both intermediate structural centrality and moderate/radical amino acid changes: E6:10, E6:78, E2:135, E2:136, and L1:76 (Table 3). Two others were both radical and convergent: E2:341-Cys/Leu/Trp and L2:378-Ala/Cys/Phe/Ser/Val/Tyr (Supplementary Figs. S10 and S11), where L2:378 is one of ten sub/lineage-defining convergences (Fig. 1). We also observed five additional positions with intermediate centrality and moderate sub/lineage-defining changes; seven with high centrality and conservative changes; and nine with intermediate centrality and conservative changes. Full metadata for all codons is available in Supplementary Data 1.

### HPV16 proteins relevant to HLA immunity have unique sequence features

**Fig. 7:**
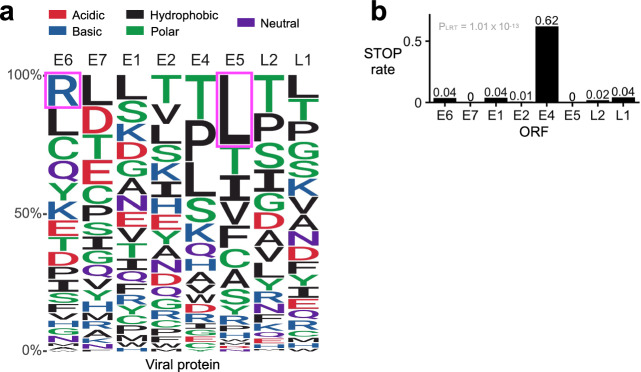
Sequence composition of HPV16 proteins. **a** Amino acid composition of each viral protein (HPV16REF; PaVE^108^). Magenta boxes denote the most overrepresented amino acids (R = Arg in E6; L = Leu in E5). Letter color denotes amino acid properties (red = acidic; blue = basic; black = hydrophobic; green = polar; purple = neutral). **b** STOP rate refers to normalized number of samples with premature STOP mutations, determined as the number of samples with a premature STOP divided by the number of STOP-proximal codons in the ORF. The *P* value refers to a likelihood ratio test (omnibus) of heterogeneity in STOP mutation rates at STOP-proximal codons across ORFs.

Beyond specific codons, our analyses also identified broader aspects of the HPV16 proteome that are consistent with a role for HLA-driven positive selection in viral evolution. E5, the viral protein that disrupts MHC class I peptide presentation, had at least three distinguishing sequence features. First, E5 contained zero Glu, Gly, Lys, or Gln residues (all non-hydrophobic) (Fig. 7a), consistent with its transmembrane nature. Second, Leu (hydrophobic) was significantly more abundant in E5 (25%) than any other protein (6–13%; *P*_Fisher_ = 6.24 × 10^−6^) (Fig. 7a). Third, E5, which disrupts MHC class I presentation, contained more 9-mer peptides predicted to be bound by MHC class I than any other viral protein (Supplementary Figs. S12 and S13).

Turning to other proteins, the highly expressed E4 protein^55^ had more samples with premature STOP mutations than any other ORF (*P*_LRT_ = 1.01 × 10^−13^) (Fig. 7b and Supplementary Fig. S14). The E7 oncoprotein, in contrast, had zero premature STOP mutations, consistent with its critical role in carcinogenesis^10^.

## Discussion

Our study characterized the molecular, evolutionary, and immunological variation of HPV16 protein-coding ORFs in unprecedented detail. By performing the largest HPV16 natural selection analysis to date, we identified 56 codons with evidence of positive selection favoring amino acid changes. Only nine of these codons were previously reported in selection analyses of HPV16^7,13–15^, which focused on the oncoproteins or had limited power due to smaller datasets. The 56 codons were enriched for both sub/lineage-defining sites and CTL epitopes documented in the IEDB, particularly for the known cervical precancer/cancer risk allele HLA-B*07:02. Our results suggest that host immunity has exerted selective pressure on HPV16, thereby influencing viral diversity and evolution.

Our 56 positively selected codons exhibited several types of evidence for positive selection. First, the codons all had significant *d*_N_ > *d*_S_ across multiple statistically plausible phylogenetic trees. Additionally, many of these codons had (1) *π*_N_ > *π*_S_ among all samples, indicating that amino acid diversity at these positions is common; (2) evolutionary convergence, with the same mutations arising multiple times over the course of HPV16 evolution; (3) intermediate/high predicted network centrality in their protein structures, implying functional significance; and/or (4) moderate/radical amino acid changes.

Our finding of positive selection in the HPV16 genome is not inconsistent with previous work indicating genetic drift predominates in the evolution of HPV genome sequence composition^12^. Further, purifying selection is the dominant form of selection in virtually all genes and organisms^40^. Our 56 positive selection candidates are interesting precisely because they represent an exception to the rule.

Rare amino acid variants at positively selected positions within E1 were enriched in cervical precancers/cancers compared to controls, suggesting that variants at these positions enabled viral persistence, e.g., through immune escape, thus potentiating carcinogenesis. This rare variation occurs in 11 of 854 cases with A1/A2-sublineage virus, and it is possible they could represent passenger mutations arising during prolonged infection rather than drivers of persistence or progression. However, given our previous observation that such rare viral variation is overall more common in controls^10^, positively selected positions are atypical in that they instead have more rare amino acid variation in precancers/cancers.

We evaluated associations between positive selection and a biologically established a priori hypothesis known to operate in host/pathogen coevolution: HLA-mediated immune recognition^56^. We reasoned that HLA-related selection pressures are likely to be greatest for early (E) proteins that are expressed where immune exposure is greatest, i.e., in the lower epithelial layers^57^. Consistent with this, we observed more positively selected positions in the E proteins, and more rare amino acid variants in cervical precancers/cancers than controls, specifically at positively selected codons in E1. These E1 variants could contribute to persistence and/or progression: E1, E6, and E7 transcript levels are all associated with progression^58,59^, and because E1 induces DNA damage, failure to control its nuclear levels (e.g., due to a mutation at position 100 in the nuclear export signal) could lead to genomic instability, integration, or other changes promoting carcinogenesis^60^. In the E1 DNA-binding domain, positions 208, 219, and 220 were predicted to be close to the interacting DNA molecule, with variants at 208 predicted to destabilize the protein. Furthermore, position 294 is located in experimentally verified epitopes restricted by HLA-A*02:01 and -A*68:01 (IEDB IDs: 111459 and 769595), and recent evidence from A*02 knock-in mice suggests E1 epitopes induce the strongest CTL responses of any HPV16 protein in terms of both magnitude and breadth (Gayton et al. 2025, “Leveraging Computational AI-based Tools to Design a Rational Therapeutic T cell-based Vaccine for HPV”, International Papillomavirus Society conference presentation). This evidence is consistent with E1 having a case/control difference at positively selected codons, particularly if the strongest selective pressures are due to antigen-specific immunity. By contrast, relatively few positively selected codons were observed in the viral capsid, L1. This may be a consequence of the fact that no capsid is produced until the upper layers of the epithelium, where exposure to HLA-mediated immunity and related selective pressures is minimal.

Our 56 codon candidates are targets to avoid in immunotherapies like therapeutic vaccines. The most broadly effective epitopes are generally expected to be functionally constrained regions that cannot withstand mutation or undergo positive selection. By contrast, all of the positively selected positions we report involved amino acid changes that have undergone successful viral transmission.

Experimentally verified CTL epitopes of the cervical precancer/cancer risk allele B*07:02 were enriched for positively selected codons. This provides a simple mechanistic rationale for the risk conferred by this allele: its HPV16 epitopes lack constraint, allowing the virus to evolve by positive selection. Consistent with this, B*07:02 is among the most common of the thousands of HLA-B alleles^37^, suggesting HPV16 would have frequently encountered this allele and experienced immune selection. Viral variation is important to consider in future cervical cancer GWAS because the effect of an HLA allele can differ with variation in viral epitopes, as documented for other viruses^61–63^. More broadly, understanding infection outcomes likely requires modeling both host variation and viral variation together, as well as their interaction.

Beyond individual codons, broader aspects of HPV16 molecular variation also suggest that this virus has evolved to evade HLA-based immunity. E5, one of only eight HPV16 proteins, disrupts MHC class I presentation of epitopes to CTL. Miyauchi et al.^19^ observed that no E5-derived peptides were presented on the surface of CAL-27 cells (HLA-A*26:08; B*44:03/51:01; C*07:02/16:01), but we found that E5 nonamers had more predicted MHC class I binding than any other protein. We also observed that E5 lacks Lys, which is the target for ubiquitination to signal proteasomal degradation^64^. This might suggest that E5’s amino acid composition evolved to avoid MHC presentation^65^; alternatively, given E5’s function to downregulate MHC, its expression may prevent its own presentation, shielding the protein from immune selection and allowing the accumulation of epitopes in E5 by drift. The E4 protein was also remarkable, acquiring premature STOP mutations in a significant number of samples. Given that E4 is highly expressed^55^, these STOP mutations might be positively selected during noninfectious within-host persistence because they eliminate the expression of epitopes; alternatively, E4 STOP mutations may be a byproduct of nonsynonymous mutations in the overlapping E2 ORF. Further analysis is required to differentiate between these hypotheses, but the high *π*_S_ of E4 is more consistent with the latter.

E6:10 is an especially compelling case of positive selection. Here, codon position 2 (100% nonsynonymous) is a hypermutable TpC site subject to APOBEC3 mutagenesis; therefore, biased mutation rather than positive selection could have been suspected as the cause of *d*_N_ > *d*_S_. However, when we inferred the codon’s evolutionary history, the expected APOBEC3 mutation (TpC→TpT) was never observed. Furthermore, Arg→Gly converged multiple times at this position, creating CpG sites—a case of positive selection overcoming the opposing force of mutation bias to create a hypermutable motif. Thus, this position’s centrality, *π*_N_ > *π*_S_, *d*_N_ > *d*_S_, radical amino acid changes in opposition to mutation bias, evolutionary convergence, functional roles, pathology associations, and sub/lineage-defining changes all suggest it has evolved in response to selective pressures. Previous work^34^ suggested that E6:10-Arg→Gly remains bound by B*07:02 but alters T cell contact residues, with potential implications for vaccine design and cervical cancer. Of note, E6:10 demonstrates that the mere presence of a hypermutable motif does not necessarily imply *d*_N_ > *d*_S_ is a false positive. Elevated mutation rates caused by APOBEC3 or CpG hypermutability may even supply the variation that allows subsequent positive selection.

Our study has limitations. Our list of codons under positive selection is based on comparative sequence analysis, and laboratory or clinical studies are needed to confirm their functional relevance. CTL epitope records in the IEDB are subject to change and are biased in favor of the HPV16 A lineage and the E6 and E7 oncoproteins. However, ascertainment bias of epitopes would not be expected to favor positively selected codons or sub/lineage-defining sites across the whole genome. Further, the most overrepresented protein in the IEDB was E7, but this contained only five sub/lineage-defining sites and the lowest amino acid variation of any protein. Enrichment of IEDB records at positively selected codons also cannot be explained by preferential mapping of epitopes to variable (i.e., high *d*_N_) positions, because mapping only failed for one record. We limited our analyses to HLA class I, but class II also shows associations with HPV-related disease^32^ and deserves further attention. We made extensive effort to exclude multi-sub/lineage coinfections by eliminating samples with phylogenetic uncertainty, mosaic patterns, or substantial within-host variation, and by employing phylogeny-robust methods such as *π* and plausible tree sets, but cannot fully rule out this phenomenon. We also considered only a single consensus (major nucleotide) sequence for each sample and did not analyze within-host variation. However, it is known that such variation contributes to outcomes^8^, including non-silent variation in E7^10^ and viral integration^66^, and these effects warrant further study.

In conclusion, our results suggest that specific HPV16 mutations interact with host HLA alleles and influence infection outcomes. This may partly underlie the differential risks conferred by different HPV16 sub/lineages, as well as the disparate outcomes experienced by individuals infected by similar or identical viral genomes. These findings are relevant to the design of therapeutic vaccines and targeted immunotherapy, where the match between host genetics (e.g., HLA) and viral variation (e.g., CTL epitopes) can be critical for effectiveness. Our 56 positive selection candidates are important hotspots for viral protein variation that must be kept in mind when choosing viral targets. To this end, we assembled Supplementary Data 1 as a resource for the scientific community with abundant metadata on every codon in the HPV16 genome, including molecular variation, CTL epitopes, and comparisons to HPV31 and HPV35. Future studies that account for human variation, viral variation, and their interaction are needed for a full understanding of the genetic components of risk for HPV-associated disease.

## Methods

### Ethics statement

For the Kaiser Permanente Northern California (KPNC)-National Cancer Institute (NCI) HPV Persistence and Progression (PaP) cohort, the KPNC Institutional Review Board (IRB) approved use of the data, and the National Institutes of Health (NIH) Office of Human Subjects Research deemed this study exempt from IRB review. For the NCI Study to Understand Cervical Cancer Early Endpoints and Determinants (SUCCEED), written informed consent was obtained from all women enrolled in the study, and IRB approval was provided by the University of Oklahoma Health Sciences Centre (OUHSC) and the US NCI. For the International Agency for Research on Cancer (IARC) data, both local and IARC ethical committees approved all studies.

### Samples and genome sequence data

We obtained 6411 HPV16-positive cervical samples^9,10^ from women in three previously described studies: 2062 internationally collected by IARC; 3305 from the KPNC-NCI PaP cohort; and 1044 from SUCCEED. Controls were defined as women from the PaP cohort with baseline enrollment specimens that were HPV16 positive with no histologic evidence of CIN2 or worse at enrollment or during the follow-up study period, according to the electronic health records (402 with ≤CIN1 histology and 448 with normal or no histology during our study, i.e., biopsy not indicated). These controls underwent follow-up for a median of 4.3 years (interquartile range = 3.36; range = 7.85 years) with no subsequent detection of CIN2 or higher. Cases were defined as women that were HPV16 positive and diagnosed with precancer (CIN3 or adenocarcinoma in situ) or cancer (squamous cell carcinoma or adenocarcinoma). One sample per woman was analyzed, and the first available baseline HPV16-positive sample was used for sequencing.

Women included from the KPNC-NCI HPV PaP cohort underwent routine cervical cancer screening using the Hybrid Capture 2 assay (HC2; Qiagen Inc, Germantown, MD) to test for a pool of 13 carcinogenic HPV types along with cytology cotesting^67^. The HC2-positive samples were HPV genotyped using Onclarity (BD, Sparks, MD), Linear Array^®^ HPV Genotyping System (Roche Molecular Diagnostics, Pleasanton, CA), or MY09-MY11 PCR and type-specific dot-blot hybridization (Burk Laboratory, Bronx, NY)^68,69^. Women in the SUCCEED study were referred to colposcopy or treatment at the University of Oklahoma Dysplasia Clinic based at the OUHSC, after a recent abnormal Pap smear diagnosis or a biopsy diagnosis of CIN/cancer. SUCCEED sample HPV genotyping was done using the Linear Array® HPV Genotyping System (Roche Molecular Diagnostics)^70,71^. Hybridization of PCR products to linear arrays and subsequent signal detection were performed using the *Auto*-LiPA automated staining system (Innogenetics N.V., Belgium). Hybridization to both β-globin probes was required to report genotyping results. A hybridization signal was considered positive when an unambiguous, continuous band was observed on the array. Samples from women in the IARC biobank were collected as part of the IARC-coordinated cervical cancer case series, cervical cancer case–control studies, and population-based HPV prevalence surveys from 39 countries worldwide. IARC HPV genotyping was conducted using a using a GP5+/6+-based PCR system^72^ in one centralized laboratory (Department of Molecular Pathology, Vrije University, Amsterdam, the Netherlands).

A single consensus HPV16 genome sequence was generated for each sample using our Ion Torrent sequencing assay^73^, where each position in the viral genome was assigned the major (most common; variant allele fraction [VAF] > 50%) nucleotide if its mapping quality was ≥4 and sequencing coverage was ≥6; otherwise, the position was masked (N). Only samples with ≥70% genome coverage were included.

Protein-coding ORFs refer to the HPV16REF annotations at PaVE^74^ (https://pave.niaid.nih.gov/) for E6, E7, E1, E2, E1^E4, E5, L2, and L1. Our E4 positions exclude the first six codons that also belong to E1, such that codon 1 of E4 in this study corresponds to codon 7 of E1^E4 (GCA). The following sub/lineage reference genomes were used: A1 and A1/A2 = HPV16REF; A2 = AF536179; A3 = HQ644236; A4 = AF534061; B1 and B = AF536180; B4 = KU053914; C1 and C = AF472509; C2 = HQ644244; C3 = KU053920; C4 = KU053925; D1 = HQ644257; D2 = AY686579; D2/D3 and D3 = AF402678; and D4 = KU053933. Sequences were aligned and normalized (insertions removed) with respect to HPV16REF (Supplementary Data 9). Pairwise distances between aligned sequences were computed using MEGA11^75,76^.

### Filtering and exclusions

Of 6411 initial HPV16 whole genome sequences, we ultimately retained 4704 sequences for analysis after extensive filtering. We sequentially removed: samples with previously documented multi-sub/lineage coinfection, poor sequence quality, or incomplete metadata (359 excluded, leaving 6052); samples with <70% genome coverage after masking low-coverage (<10) sites (173 additional exclusions, leaving 5879); samples with high within-host polymorphism, considered to be intermediate VAF values of 40–60% at >1 genome position (1029 additional exclusions, leaving 4850); samples with lineage assignment confidence of <95% (105 additional exclusions, leaving 4745); and samples with a premature STOP codon in any ORF (41 additional exclusions, leaving 4704). We did not exclude samples co-infected with multiple HPV types, since the presence of other types would not affect our HPV16 sequence data or evolutionary analyses, and their exclusion would systematically deplete controls.

### Sequence masking

For the intermediate dataset of 5879 sequences, we constitutively masked 171 sites (2% of the genome), including the full 135 nucleotides of the NCR (4102–4236) and 36 additional sites with low sequencing quality or high within-host diversity across multiple samples (Supplementary Data 10). Remaining sites with intermediate VAFs were masked (N) in the sample in which they occurred.

### Phylogenetic plausible tree sets

Our initial set of 6052 HPV16 genome sequences exhibited high phylogenetic uncertainty (i.e., low signal) and correspondingly high difficulty of phylogenetic inference (Pythia difficulty score = 0.83, where 0 = easy and 1 = impossible; PyPythia^77^ version 1.1.2; https://github.com/tschuelia/PyPythia). To address this, all tree-based analyses were performed with multiple plausible trees of statistically indistinguishable likelihood, i.e., “plausible tree sets”^78^. Two hundred trees were used for determining lineage assignment confidence; five trees were used for each selection analysis.

### Lineage assignment confidence

Sequences were initially classified by sublineage using previous assignments from a single maximum likelihood tree^9,10^. We then quantified the confidence of each sequence’s assignment to one of the four HPV16 lineages (A, B, C, or D) using 200 plausible trees^78^ inferred from the intermediate dataset of 4850 sequences with RAxML-NG^79^ v. 1.0.0 (GTR + FO+G4m model; https://github.com/amkozlov/raxml-ng).

To compute lineage confidence, we devised an automated classification method that varied both the tree root and pruning order over a set of replicates (Supplementary Fig. S15 and Supplementary Information). For a given tree, the confidence of each sample’s lineage assignment was calculated as the proportion of replicates in which the sample was assigned to that lineage. The following lineage representatives were used: A = PAP266425 (A1), B = PAP3508 (B1), C = IRC200686 (C1), and D = PAP139245 (D3). Each sample’s lineage confidence score was calculated as the mean confidence score across 200 plausible trees. Only the 4745 samples with >95% confidence were retained (105 excluded) for further analysis (Supplementary Fig. S16). For all subsequent phylogenetic analyses, the HPV16 tree was rooted a priori on the branch separating lineages A vs. B/C/D, following the results of previous Bayesian analyses^6,7^. Algorithm performance was benchmarked using simulation (Supplementary Fig. S17 and Supplementary Information).

As noted above, samples with evidence of co-infection by multiple HPV16 sub/lineages were excluded.

### Sub/lineage-defining sites and convergence

Sub/lineage-defining sites (i.e., diagnostic sites) were determined by identifying genome positions at which the major nucleotide allele differed between lineages or sublineages. Major alleles were required to be nearly fixed, defined as exceeding an allele frequency cut-off determined using a method adapted from receiver operating characteristic (ROC) curves (Supplementary Information). The union of sites for lineages (required frequency >91.8%; *n* = 137) and sublineages (required frequency >94.3%; *n* = 156) was collectively referred to as sub/lineage-defining sites (*n* = 158) and used for all downstream analyses. Codons were considered sub/lineage-defining if they contained at least one sub/lineage-defining site. Convergence at the molecular level was determined as the median signal across the top five plausible whole-genome trees.

### Nucleotide diversity

Nucleotide diversity (*π*), the mean number of pairwise differences per nucleotide position in a population^80,81^, was estimated separately for nonsynonymous (*π*_N_) and synonymous (*π*_S_) viral genome sites^82^ using SNPGenie^83^ or OLGenie^84^. OLGenie was used for codons overlapping multiple ORFs in alternate reading frames, where *π*_N_ and *π*_S_ were estimated using *π*_NN_ and *π*_SN_, respectively. Differences between *π*_N_ and *π*_S_ were evaluated using Z tests by bootstrapping codons to estimate the standard error of mean *π*_N_ - *π*_S_ (1000 replicates, resampling alignment columns; null hypothesis: *π*_N_ - *π*_S_ = 0). Differences between *π*_N_/*π*_S_ ratios (i.e., *π*_N1_/*π*_S1_ vs. *π*_N2_/*π*_S2_) were evaluated using a custom bootstrap method where codons from each site class were resampled for each replicate (null hypothesis *π*_N1_/*π*_S1_ - *π*_N2_/*π*_S2_ = 0).

### Sequence composition and premature STOPs

To calculate normalized STOP rates for each ORF, the number of samples with a premature STOP was divided by the number of STOP-proximal codons for each ORF (HPV16REF sequence). These calculations refer to the penultimate dataset of 4745 samples, which had already been filtered for all other quality control criteria. Heterogeneity in STOP mutation rates across ORFs was tested using a Poisson GLM (predictor = ORF, offset = log-transformed STOP-proximal codon count, link = log).

### Predicted CTL epitope content using NetMHCpan

We predicted the epitope content of each HPV16 protein using the most common variant (haplotype) of each sublineage in our dataset (Supplementary Data 11). For each variant, NetMHCpan-4.1^85^ (https://services.healthtech.dtu.dk/services/NetMHCpan-4.1) was used to query all viral protein 9-mers (amino acid substrings of length nine) against representative HLA class I alleles. Only 9-mers were queried because this is the most common CTL epitope length and dominates the training data of NetMHCpan.

### Protein structure prediction and network centrality

To predict protein structures, ORFs were translated from each of the 16 sublineage representative genomes, yielding 81 distinct protein variants (some were identical). For E2, full-length E2 was modeled; for E4, the full E1^E4 structure was modelled. We then predicted the monomeric 3D protein structure for each protein variant using AlphaFold2^86^. The confidence of each residue in each structure was calculated as a predicted local distance difference test (pLDDT) score with AlphaPickle (https://github.com/mattarnoldbio/alphapickle).

Each amino acid’s network centrality in its predicted protein structure was estimated using second order degree centrality^87^ (SODC). Distinct second order contacts for each residue were tallied by identifying non-bonded interactions (hydrogen bonds, salt bridges, Van der Waals, hydrophobic, and cysteine-cysteine disulfide interactions) between each residue pair. A residue pair was classified as a contact if it participated in any interaction type, and an N-by-N contact matrix (N = number of residues in the protein) was created for each structure. Subsequent centrality analyses were only performed for positions with high confidence (pLDDT >70) (Supplementary Fig. S3 and Supplementary Data 12). Amino acid residues with high structural confidence were binned into low (bottom 30%; values < 20), intermediate (middle 40%; 20–32), or high SODC (top 30%; >32).

The predicted HPV16 E1 structure was superimposed onto the bovine papillomavirus E1 structure (PDB ID: 7APD, chain H) using the cealign^88^ command in PyMOL (The PyMOL Molecular Graphics System, Version 3.0, Schrödinger, LLC). DUET^46^ and DynaMut2^47^ were then used to predict the effects of observed amino acid variants on the protein’s thermodynamic stability using the statistic ∆∆G, the difference in Gibbs free energies of the mutant and wildtype proteins (kcal/mol; negative = destabilizing, positive = stabilizing). Following Capriotti et al.^89^, we considered |ΔΔG| > 1 to be functionally impactful.

### Protein alignments to HPV31 and HPV35

Sequencing reads for each HPV type were aligned to their reference genomes available at PaVE (HPV16/31/35). The cleaned protein sequences were aligned using MAFFT^90,91^ with default parameter settings. The aligned protein sequences were visually inspected with MEGA^76^ and AliView^92^ to ensure alignment quality, and one manual edit was made in HPV35 (S-YTP changed to SYT-P). Sequences were normalized with respect to HPV16REF.

### Amino acid physicochemical distance

We used the values of Miyata et al.^93^ to analyze physicochemical distances between amino acid pairs. Each pair’s distance was characterized as conservative (bottom 30% of distinct amino acid pairs; ≤1.720), moderate (middle 40%; 1.720–2.789), or radical (top 30%; >2.789) based on the distribution of all possible values, including those that require multiple nucleotide changes to their codons.

### Positively selected codons

We again employed plausible tree sets for our positive selection analyses: 20 ML tree searches were performed, from which the top five trees were used to perform five independent selection analyses for each method/ORF.

We inferred *d*_N_/*d*_S_ at individual codons with HyPhy (HYpothesis testing using PHYlogenies^94^; www.hyphy.org) using two methods: FEL v. 2.5 (Fixed Effects Likelihood; pervasive positive and purifying selection^95^) and MEME v. 4.0 (Mixed Effects Model of Evolution; episodic positive selection^96^). Ambiguous characters (N) were replaced with gaps (-) to force HyPhy to treat these sites as missing data. Significance was assessed using the likelihood ratio test (LRT) conducted by the software and using the suggested significance threshold^97^ of *P*_LRT_ < 0.10.

We considered only internal tree branches (lines of descent between two inferred ancestors) because these have necessarily undergone at least one round of successful transmission between hosts and experienced both within- and between-host selection^44^. Contrarily, leaves (lines of descent leading to observed samples) additionally include genetic changes that are recent (transient), occur within-host (no between-host selection), “dead end” variation (not onward transmitted, including nonviable variants), and/or sequencing error (Supplementary Fig. S5).

When referencing previous studies of positive selection in HPV16 (Fig. 4a), we refer to the codons detected by DeFilippis et al.^13^, Chen et al.^14^, Carvajal-Rodríguez^15^, and Pimenoff et al.^7^ (Supplementary Table S2). Pimenoff codons E5:48 and 65 were originally reported as codons 46 and 63 (i.e., both shifted -2); we inferred the former coordinates to be correct.

### Known CTL epitopes from IEDB

The Immune Epitope Database (IEDB^48^; www.iedb.org) was accessed February 6, 2024 (January 8, 2024 update). All 237 epitope records were downloaded that met the following criteria: Epitope = ANY; Epitope Source / Organism = ID 333760, HPV16; Host = Human; Assay = T Cell + MHC Ligand; Outcome = Positive; MHC Restriction = Class I; Disease = ANY. These represented 220 unique peptide sequences after removing chemical modifications.

Many IEDB records contained missing or inconsistent protein (antigen) names or coordinates. Thus, we de novo mapped each epitope sequence to the HPV16REF proteome using R::Biostrings^98^ and related libraries, resulting in 216 records with unique mapping locations, zero with multiple mappings, and four with no mappings. For those not successfully mapped, two were located manually (3184:AMSAARSSR, 111297:FYSRIREL), one was excluded because it precedes the E6 start site used in this study (41674:MHQKRTAMF), and one could not be located (244063:RRYPYPAR), leaving 218 unique epitope sequences (Supplementary Data 6).

Of the 218 unique epitopes, we retained 182 with a typical CTL epitope length of 7–11 amino acids^19,99^ and excluded the remaining 36 of length 15–36 amino acids for downstream analysis (Fig. 5a and Supplementary Data 7). Each IEDB epitope sequence was compared to all protein variants in our dataset to determine the mean p-distance from each sub/lineage.

### Computational and statistical analysis

This work utilized the computational resources of the NIH HPC Biowulf cluster (https://hpc.nih.gov). Data processing and statistical analyses were performed using R^100^ version 4.4.1 (2024-06-14) with base R, Biostrings, boot, BSGenome, corrplot, data.table, dplyr, feather, GenomicFeatures, GenomicRanges, ggrepel, ggseqlogo, gridExtra, jsonlite, patchwork, pwalign, RColorBrewer, rtracklayer, scales, seqinr, stringdist, and tidyverse^101^ libraries; with Microsoft Excel; and with custom Perl and Python scripts (see Code availability). All reported *P* values are based on two-sided statistical tests. LRT refers to likelihood ratio tests. GOF refers to Chi-square Goodness of Fit tests. Tests for enrichment among classes of sites or codons used Fisher’s exact tests. Visualizations were made using ggplot2^102^ and annotated using Microsoft PowerPoint.

### Reporting summary

Further information on research design is available in the Nature Portfolio Reporting Summary linked to this article.

## Supplementary information

Supplementary Information

Description of Additional Supplementary Files

Data 1

Data 2

Data 3

Data 4

Data 5

Data 6

Data 7

Data 8

Data 9

Data 10

Data 11

Data 12

Reporting Summary

Transparent Peer Review file

## Source data

Source Data

## Data Availability

Sequence data are publicly available and were obtained from the previous studies cited in the main text (Mirabello et al.^9^^,^^10^; GenBank accessions MG847621-MG850835). Source data are provided in the Supplementary Information of this paper.

## Code availability

Perl, Python, R, and command line code used for data analysis are available at https://github.com/chasewnelson/HPV16-molecular-evolution and archived under 10.5281/zenodo.19481521.
