# Supplementary material for: HPV16 genetic variation provides evidence of positive natural selection driven by HLA class I
Source: Nat Commun. 2026 Jun 2;17:7064. doi: 10.1038/s41467-026-73531-0 (PMC13392105; doi:10.1038/s41467-026-73531-0)
Supplement: Supplementary file 1 — Supplementary Information [file 41467_2026_73531_MOESM1_ESM.pdf]

Supplementary Information

HPV16 genetic variation provides evidence of positive natural selection driven by HLA class I

Chase W. Nelson[1,*], Sambit K. Mishra[1,2], Michael Dean[1], Colm Ohuigin[3,4], Robert D. Burk[5], Bin Zhu[1], Difei Wang[1,2], Laurie Burdett[1,2], Mathias Viard[3,4], Hyo Jung Lee[1,2], Aimee J. Koestler[1], Apurva Narechania[6], Zigui Chen[7], Nicolas Wentzensen[1], Mark Schiffman[1], Gary M. Clifford[8], Elizabeth Suh-Burgmann[9], Thomas Lorey[10], Mary Carrington[3,4,11], Meredith Yeager[1,2,12], Lisa Mirabello[1,*]

[1]Division of Cancer Epidemiology and Genetics, National Cancer Institute, National Institutes of Health, Rockville, MD 20850, USA; [2]Cancer Genomics Research Laboratory, Frederick National Laboratory for Cancer Research, Rockville, MD 20850, USA; [3]Basic Science Program, Frederick National Laboratory for Cancer Research, National Cancer Institute, Frederick, MD, USA; [4]Laboratory of Integrative Cancer Immunology, Center for Cancer Research, National Cancer Institute Bethesda, MD, USA; [5]Departments of Pediatrics, Epidemiology and Population Health, Microbiology and Immunology, and Obstetrics & Gynecology and Women's Health, Albert Einstein College of Medicine, NY 10461, USA; [6]Institute for Comparative Genomics, American Museum of Natural History, New York, NY 10024, USA; [7]Department of Microbiology, The Chinese University of Hong Kong, Hong Kong SAR, China; [8]Early Detection, Prevention and Infections Branch, International Agency for Research on Cancer, 150, Cours Albert Thomas, F-69372 Lyon cedex 08, France; [9]Division of Research, Kaiser Permanente Northern California, Pleasanton, CA 94588, USA; [10]Regional Laboratory and Women's Health Research Institute, Division of Research, Kaiser Permanente Northern California, Pleasanton, CA 94588, USA; [11]Ragon Institute of Massachusetts General Hospital, Massachusetts Institute of Technology and Harvard University, Cambridge, MA 02139, USA; [12]Department of Biology, Hood College, MD 21701, USA.

*Corresponding author(s): chase.nelson@nih.gov; mirabellol@mail.nih.gov

Contents:

1. Methods
2. Figures
3. Tables
4. Data file descriptions
5. References

1. Methods

Code and custom scripts

All custom scripts and accompanying documentation are freely available at https://github.com/chasewnelson/HPV16-molecular-evolution.

Filtering and exclusions

To determine read depth at each genome position, we first reduced BED files by summing coverage values in the overlap between the first and last amplicons using the custom script

`BED_reduce_cov_to_seqlength.py`. Sites with coverage < 10 were masked in the whole-genome alignment using the custom script `FASTA_mask_sites_by_coverage.py`.

Masking

VCF files were processed to split lines that reported more than one variant onto multiple lines, using the custom script `VCF_splitter.py`. Positions with high levels of within-sample polymorphism, termed 'intermediate VAF [variant allele fraction]', were defined as iSNVs (intrahost single nucleotide variants) with VAF values of 40–60%. Sites were globally masked for exhibiting high numbers of iSNVs, multiple samples with intermediate-VAF iSNVs, indels, or lack of an L- or U-shaped VAF distribution. The following 14 sites were identified and globally masked: 256, 257, 1055, 1297, 1936, 1939, 2949, 3193, 3313, 3798, 6471, 6474, 7895, and 7896. Masking was performed using the custom script `FASTA_mask_sites.py`. In total, 13 (7.6%) of these constitutively masked sites fell in protein-coding regions, covering 0.16% of the genome.

Sub/lineage-defining sites

Allele frequency thresholds for sub/lineage-defining sites were determined as the frequency (x axis value) which minimized the distance between the number of sites (y axis value) and the top right corner of the unit square (axes normalized to 1,1). This was carried out separately for lineages (137 sites; optimal frequency cut-off of 91.8%) and sublineages (156 sites; optimal frequency cut-off of 94.3%) and the combined set (union) was used (six phylogenetically distinct sub/lineages).

Phylogenetic uncertainty and lineage assignment

Lineage assignment confidence was determined as the average signal across a plausible tree set[1] of 200 maximum likelihood trees using the custom algorithm implemented in the script `clade_assignment.py`. Only parsimony starting trees were used, because in a preliminary run of 100 searches, random starting trees always yielded inferior likelihood values (non-overlapping distributions), consistent with Morel et al.[1].

Given the challenges of rooting the HPV16 phylogeny[2,3], our algorithm was designed to be root-agnostic. For a given tree, each replicate works as follows: (1) randomly root at one of the clade (lineage) representatives; (2) randomly choose one other representative; (3) step toward the root one internal node (inferred ancestor) at a time, until a node is reached from which more than one clade representative descends; (4) back up one node and prune the subtree (Suppl. Fig. S15). All sequences within the pruned subtree are then categorized as members of the step 2 representative's lineage. This process is repeated from step (2) until only two representatives are left (the root and one other representative) at which point the replicate is complete. Given n clades each with one representative, there are n possible rootings; once a root is chosen, there are $n - 1$ remaining representatives to be examined in random order. This implies $n(n - 1)!$ possible rootings and orderings. We therefore took a random rather than exhaustive approach and limited our analysis to the four HPV16 lineages (A, B, C, and D). Time required was approximately 10 minutes per tree. Lineage confidence calculations were limited to replicates in which a classification was made, i.e., replicates in which a sample was 'unclassified' were excluded. More computationally efficient solutions are possible (*in preparation*).

To select a representative sequence for each lineage, we identified the sequence in our dataset that had the smallest p-distance from the reference genome of the lineage's most

prevalent sublineage: A = PAP266425 (sublineage A1), B = PAP3508 (B1), C = IRC200686 (C1), and D = PAP139245 (D3).

Confidence scores clustered near 100% (high confidence) with outliers falling below 95% (low confidence) (Suppl. Fig. S16).

Algorithm performance was benchmarked using simulation to analyze a five-lineage tree (clades = a, b, c, d, e) after adding a single ambiguous sample (x) that does not belong to any existing clade (i.e., placed on a branch between two ancestors of different clades) (Suppl. Fig. S17a). Experimentation with 10, 100, 1000, and 10,000 replicates revealed that 1000 was sufficient to yield unambiguous results (**`execute_clade_assign_replicates.py`**) (Suppl. Fig. S17b,c). Analyses were carried out with **`clade-conf.R`**.

Trees were visualized using FigTree v1.4.4 (http://tree.bio.ed.ac.uk/software/figtree/).

Molecular convergence

Molecular convergence (i.e., evolutionary homoplasy) was quantified as the average signal across a plausible tree set of the top five maximum likelihood trees using the custom script **`homoplasy_identifier.py`**.

For each tree, samples with ambiguous characters were removed using ETE 3[4] (https://etetoolkit.org/) and the number of remaining nucleotide alleles exhibiting non-monophyly was counted (i.e., number of nucleotides that arose more than once in the evolutionary tree). A site was considered to have convergence if it had ≥2 nucleotides with non-monophyly (i.e., the major allele at a polymorphic site is always non-monophyletic and was excluded).

NetMHCpan-4.1

NetMHCpan-4.1 was used to predict MHC class I binding for all nonamers (9 amino acid substrings) of each protein, using the most common amino acid haplotype of each sublineage, limiting to those with the minimum number of masked (X) positions (Suppl. Data 11).

Nonamers were queried against 27 representative HLA class I alleles, determined as the union of supertype representatives provided by NetMHCpan[5] and those listed by Grifoni et al.[6]: HLA-A*01:01, A*02:01, A*03:01, A*11:01, A*23:01, A*24:02, A*26:01, B*07:02, B*08:01, B*15:01, B*27:05, B*35:01, B*39:01, B*40:01, B*44:02, B*44:03, B*58:01, C*03:03, C*04:01, C*05:01, C*06:02, C*07:01, C*07:02, C*08:02, C*12:03, C*14:02, and C*15:02.

Protein structure prediction

We predicted protein structure for reference protein variants of each of the 16 sublineages: A1 (HPV16REF), A2 (AF536179), A3 (HQ644236), A4 (AF534061), B1 (AF536180), B4 (KU053914), C1 (AF472509), C2 (HQ644244), C3 (KU053920), C4 (KU053925), D1 (HQ644257), D2 (AY686579), D3 (AF402678), and D4 (KU053933). For estimating second order degree centrality, only high-confidence (pLDDT >70) residues were considered, which was true for 68% (1638 of 2422) of all amino acid residue positions and 64% (88 of 137) of sub/lineage-defining positions.

E6* is sometimes expressed but is labile, quickly degraded, and has unknown function; therefore it was not modelled[7]. E4 is expressed as E1^E4[7,8] and was modelled as such; codons were subsequently renumbered such that E4:1 corresponds to the first full codon in the E4 portion, eliminating any redundancy with E1. E2 was modelled as full-length E2 rather than E8^E2. Both versions of E2 are highly expressed and have known functions, but E8^E2 omits

the N-terminal half of E2 and the remaining portion (shared with full-length E2) does not differ substantially in predicted structure between the two.

The Immune Epitope Database (IEDB)

Files were downloaded from the IEDB for antigens, epitopes, receptors, references, assays/T cell, and assays/MHC in TSV format. We mapped the IEDB epitopes to the HPV16 proteome as follows: in R[9], a BSGenome[10] object was created for HPV16REF; a Biostrings[11] DNAStringSet was created for the ORFs (Suppl. Table S1); a reference proteome was created by translating the ORFs into a AAStringSet; raw epitope peptides were converted into a AAStringSet; each epitope was queried against the reference proteome using vMatchPattern() with the "auto" algorithm and a maximum mismatch of 30% epitope length; and Levenshtein distance (accommodates indels) from the reference protein was computed using pwalign[12] stringDist().

Each codon was characterized with respect to the mean number of differences between the sub/lineage's encoded amino acid and each overlapping epitope of record. For determining such distances, seven epitopes mapped to one or more sites constitutively masked during upstream QC: E6:51-52 (15173:FAFRDLCIV, 110575:FAFRDLCIVY, 2004520:AFRDLCIVY, 110226:FRDLCIVY, 29519:IVYRDGNPY), E1:358-359 (2150441:FELSQMVQW), and E2:65 (911791:LAVSKNKAL). Thus, these records were excluded from such calculations, i.e., only 174 of 181 epitopes could be characterized with respect to sub/lineage distances and frequencies.

Three pairs of epitopes mapped to identical positions but differed in sequence (111055:VYDFAFQDL and 71988:VYDFAFRDL; 111184:CYSVYGTTL and 7439: CYSLYGTTL; and 64817,64818:TLGIVCPI and 79659:TLIDVCPI).

2. Figures

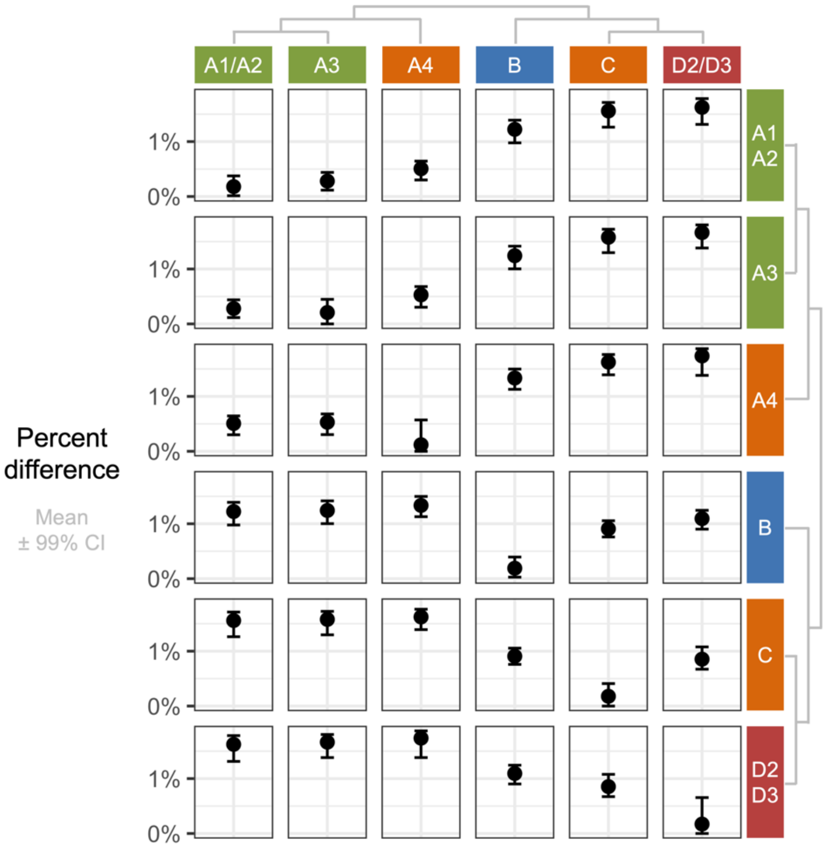

Supplementary Figure S1. Mean pairwise differences between HPV16 whole genome sequences of six sub/lineages. Bars show the central 99% of p-distance values. The number (n) of sequences for each sub/lineage were: A1/A2 = 3559, A3 = 104, A4 = 220, B = 136, C = 201, D2/D3 = 328. Phylogenetic tree branch lengths (grey) are not to scale. Plots are symmetric about the diagonal. The maximum number of differences observed for any one pair occurs between IRC200242 (A4) and PAP3144 (D2): 143 single nucleotide differences, for a p-distance of 1.86%. All ambiguous positions were removed for each sequence pair (pairwise deletion option) in MEGA11[13,14].

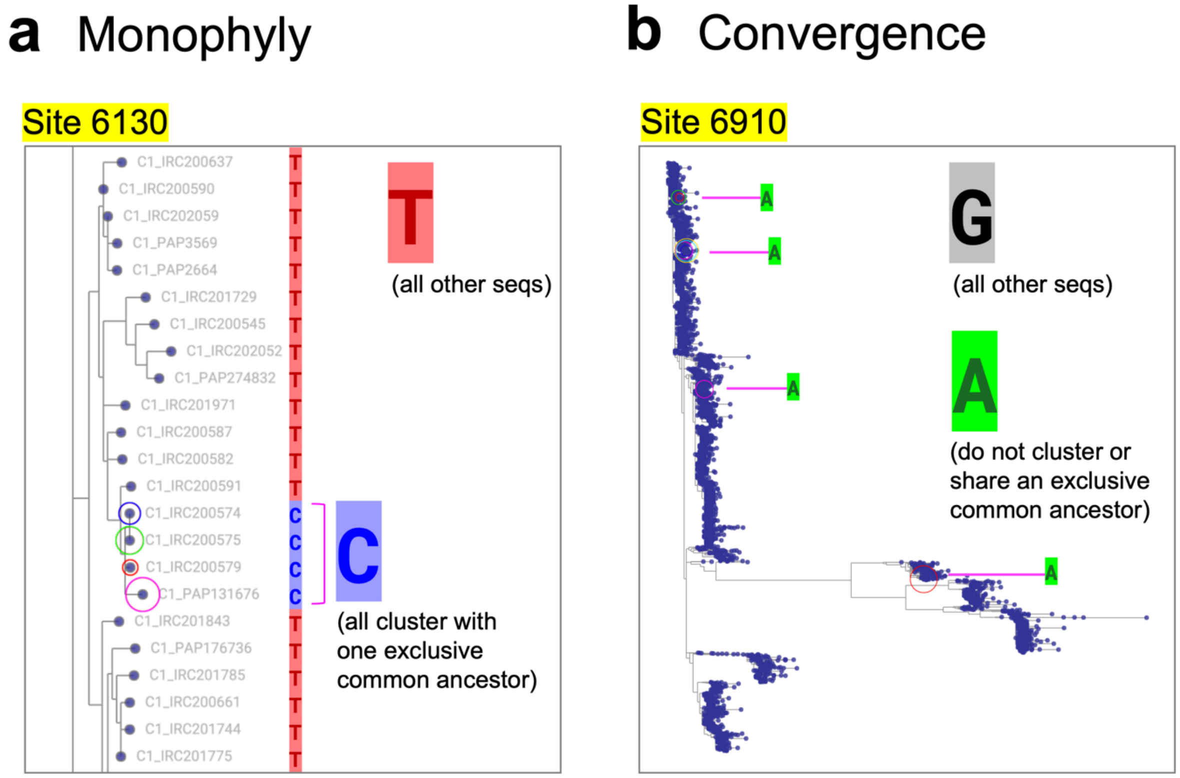

Supplementary Figure S2. Examples of monophyly and convergence (homoplasy) in the HPV16 phylogenetic tree. a Site 6130 exhibits monophyly because all samples with the minor allele (C) form a cluster descended from a single common ancestor, this cluster contains no other alleles, and C occurs nowhere else in the tree. **b** Site 6910 exhibits convergence because samples with the minor allele (A) have evolved multiple times independently and do not share a common ancestor from which no other alleles are descended. Samples are shown using the single tree with the best maximum likelihood (bestTree) and visualized using Taxonium[15].

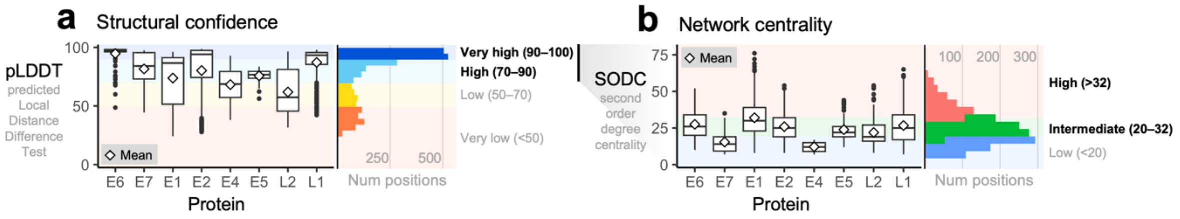

Supplementary Figure S3. Predicted protein structure confidence and network centrality. Boxplots show distributions of values for individual amino acid positions in each protein, where numbers (n) of residues are: E6 = 151, E7 = 98, E1 = 649, E2 = 365, E4 = 86 (3′-spliced portion), E5 = 83, L2 = 473, and L1 = 505 amino acids. **a** Monomeric protein structures were modeled for each viral protein using AlphaFold2[16]. Positions with predicted Local Distance Difference Test (pLDDT) values of >70 were considered to have high structural confidence and estimable second order degree centrality (SODC) and were retained for subsequent analyses. **b** SODC values were categorized as low (bottom 30%; <20), intermediate (middle 40%; 20–32), or high (top 30%; >32). Values shown are for the A1 sublineage reference (HPV16REF) variant of each protein.

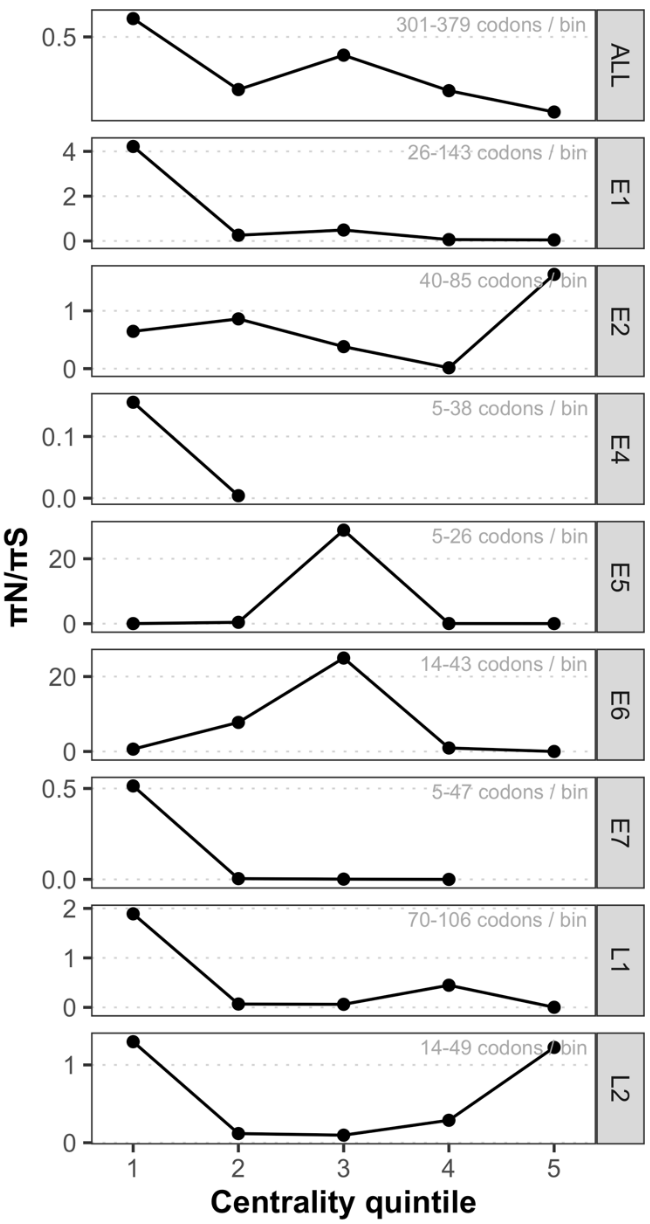

Supplementary Figure S4. π_N/π_S as a function of second order degree centrality (SODC) by viral ORF/protein. Only positions with high structural confidence (pLDDT >70) were considered. π_N/π_S (variable y axis) was estimated using all samples/clades. SODC quintiles were defined based on whole-proteome decile values: 6–16, 19–21, 23–28, 29–36, and 37–78. Bins with fewer than 5 codons were excluded. Quintiles were used because deciles were often of insufficient size for individual ORFs.

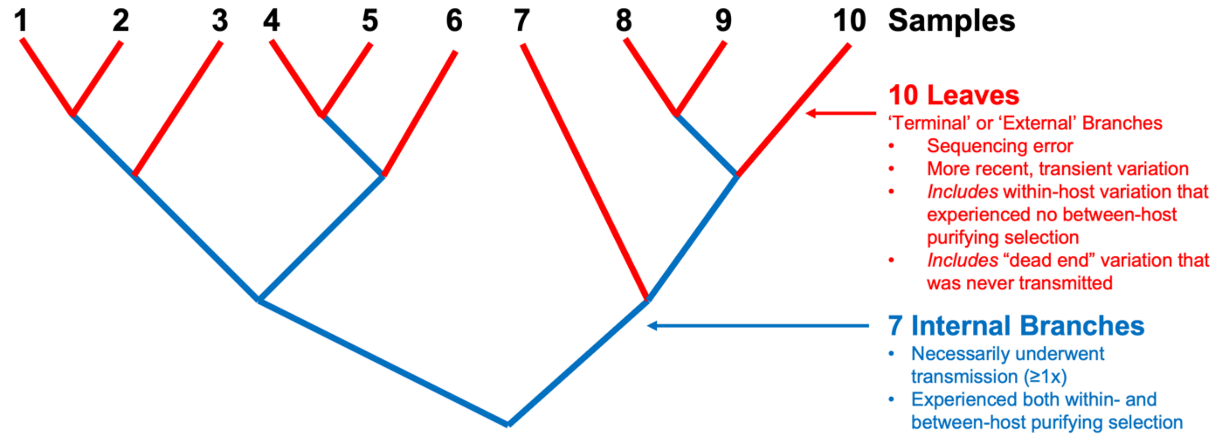

Supplementary Figure S5. Illustration of leaves vs. internal branches in a phylogenetic tree. The phylogenetic tree shows inferred lines of descent for ten samples. Leaves (red) denote the external (terminal) branches in the tree, i.e., the evolutionary lineages leading directly to an observed sample from its most recent common ancestor with another sample. By contrast, internal branches (blue) denote the evolutionary lineages which lead from one inferred ancestor to another inferred ancestor. Whereas leaves necessarily include transient, recent, within-host, and any "dead end" variation, internal branches must necessarily have undergone at least one round of successful transmission[17].

a

sample	status	seq	N samples w sequence
PAP177337	CIN3+	KQGLFXLN	1
PAP228214	control	NEELFXLN	3
PAP222082	CIN2		
PAP2958	CIN3+		
PAP108218	CIN2	NQELFXLN	5
PAP1335	CIN3+		
PAP164548	CIN3+		
PAP254496	CIN3+		
PAP2813	CIN3+		
PAP1797	CIN3+	NQVLFXLN	1
PAP286382	CIN3+	NQGVFXLN	1
PAP200626	CIN2	NQGLFXMN	6
PAP205830	CIN2		
PAP293019	CIN2		
PAP195083	CIN3+		
PAP222030	CIN3+		
PAP2930	CIN3+		

b

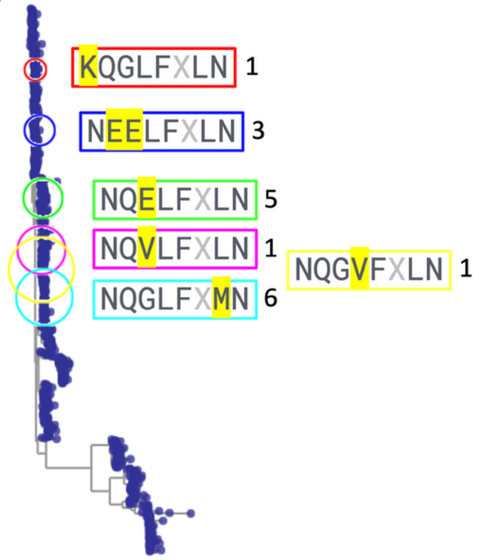

Supplementary Figure S6. Rare variation in HPV16 A1/A2 at positively selected positions in E1. Rare (minor allele frequency [MAF] <5%) variants were analyzed at the eight positively selected positions in E1: 38, 78, 100, 208, 219, 220, 294, and 597 (major haplotype of these concatenated positions = NQGLFXLN). Positions 78, 100, 208, 220, and 294 are sub/lineage-defining. Only variants in A1/A2 (of 2243 samples) are shown; of these, all rare variants occurred in A1 (none in A2). Position 220 was masked (X) for this analysis because this position had a minor allele (T) that is not rare, with MAF > 5% (S = 73.4%, T = 26.4%, L = 0.2%). **a** Status and alleles of samples with rare variation at positively selected E1 positions. These included 11 cases, 5 CIN2, and 1 control. **b** Phylogenetic tree representing all unique E1 sequences. Colored boxes show evolutionary clades with rare variants (number of samples on the right). Tree generated using Taxonium[15].

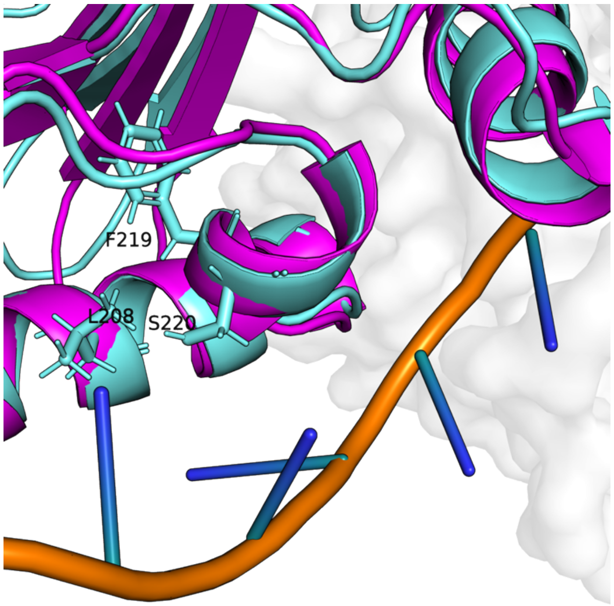

Supplementary Figure S7. Predicted HPV16 monomeric E1 structure superimposed onto the experimentally-determined bovine papillomavirus hexameric E1–DNA complex. Of the E1 residues showing evidence for positive selection, we highlight three residues (L208, F219, and S220) in the monomeric HPV16 E1 structure (cyan) superimposed onto the hexameric BPV E1 structure (PDB ID: 7APD, chain H) in complex with DNA (orange). These three residues are in proximity of the bound DNA molecule, suggesting their possible role in DNA binding. The structures were aligned using the cealign command in PyMOL (The PyMOL Molecular Graphics System, Version 3.0 Schrödinger, LLC).

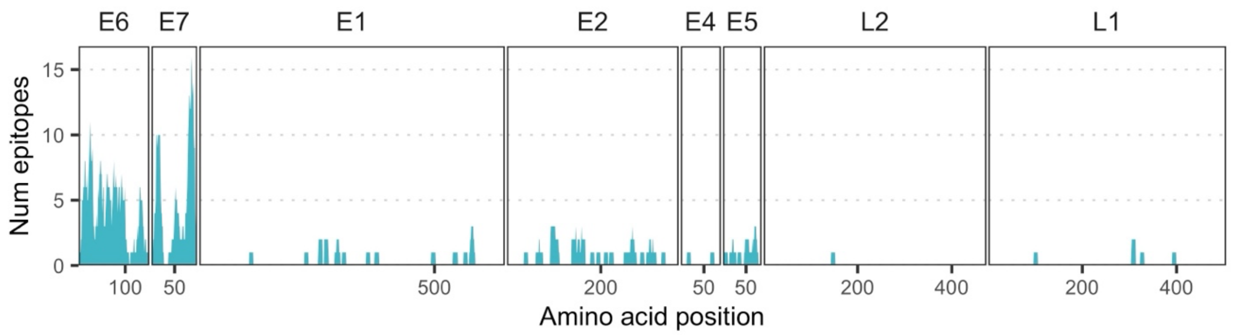

Supplementary Figure S8. HPV16 IEDB CTL epitope coverage by protein. Bar height indicates the number of distinct IEDB[18] (www.iedb.org) epitope records that overlap each amino acid residue position in each protein. Oncoprotein residues with no epitopes mapped: E6:109–110 and E7:28–36.

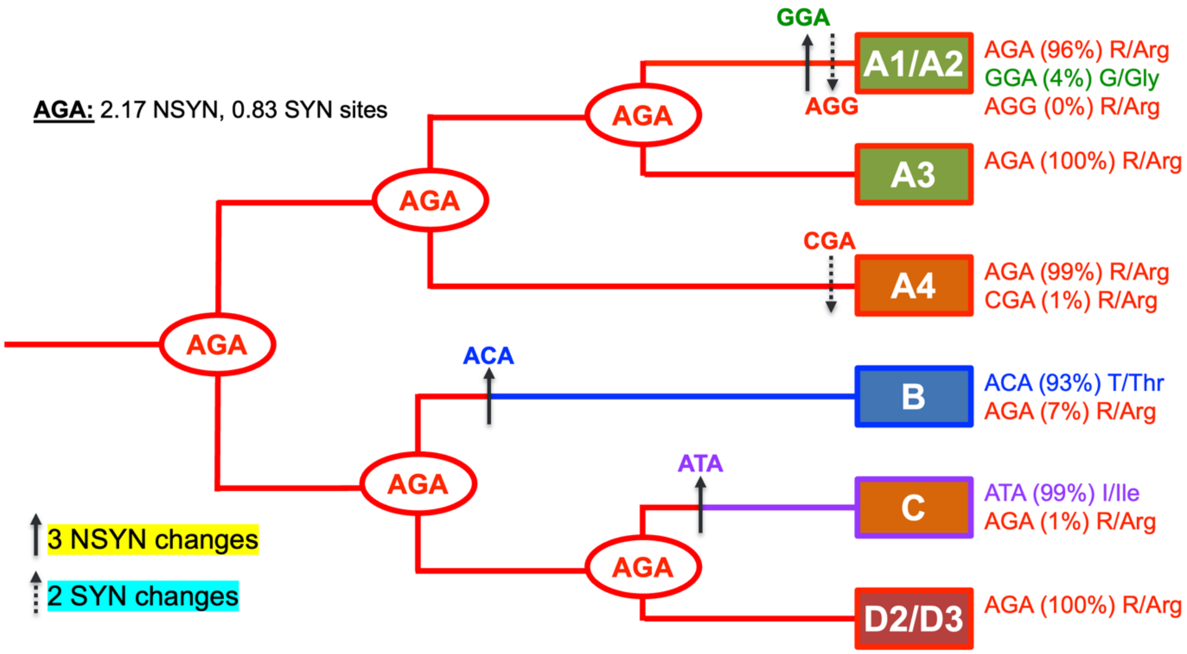

Supplementary Figure S9. HPV16 E6:10 codon evolution and frequencies. Branch lengths are not to scale. AGA contains 2.17 nonsynonymous (NSYN) sites and 0.83 synonymous (SYN) sites.

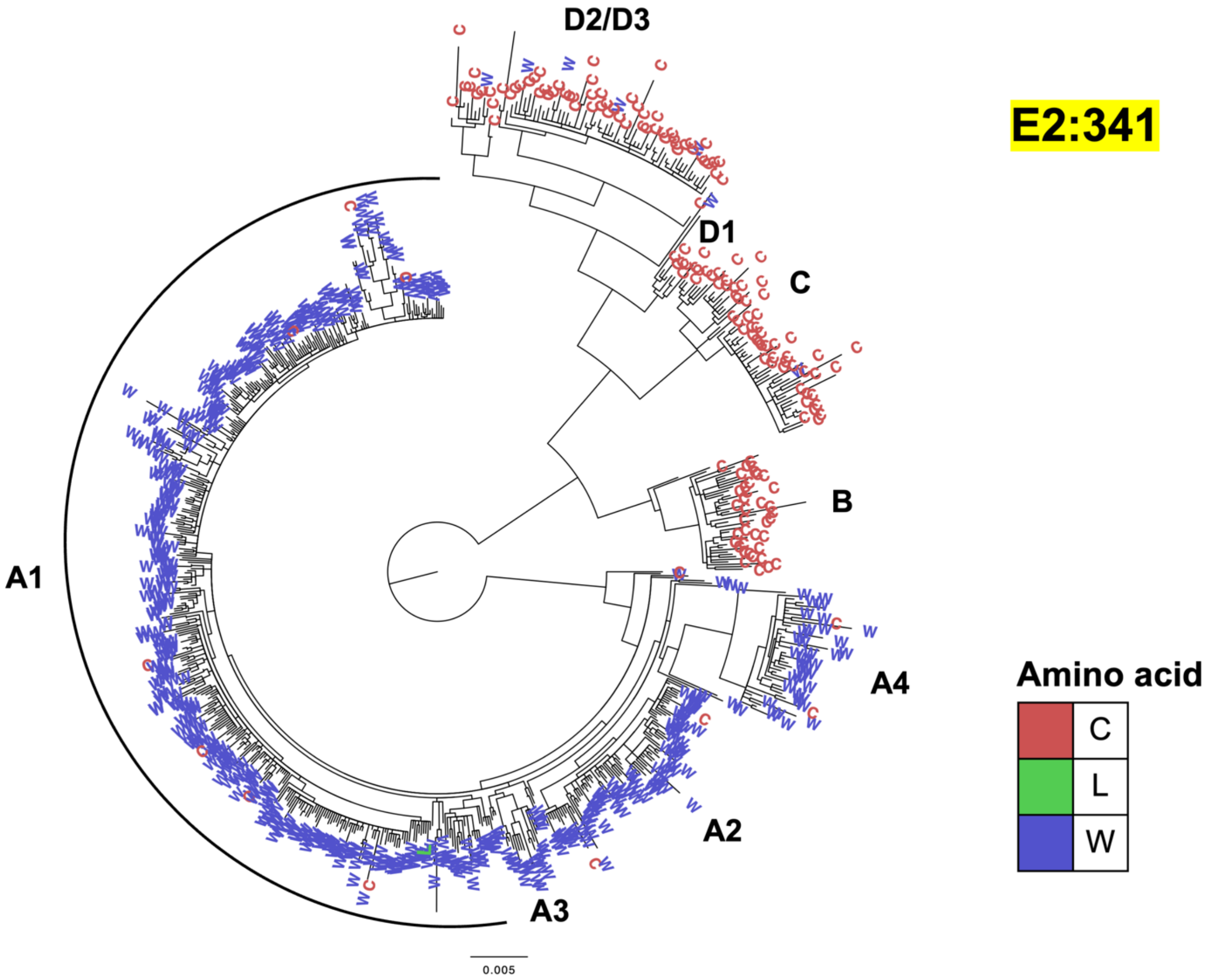

Supplementary Figure S10. HPV16 E2:341 amino acid variation and convergence. Amino acid alleles at this protein position are overlaid on the maximum likelihood phylogenetic tree inferred from the whole genome (nucleotide) by RAxML-NG (the best likelihood from 20 starting trees searched). Only samples (leaves) with unique E6 nucleotide sequences are shown, determined using the remove-duplicates script of HyPhy (https://github.com/veg/hyphy-vision) to simultaneously detect and prune duplicate samples. Bold labels indicate viral sub/lineage (clade). Colors and leaf labels denote amino acid.

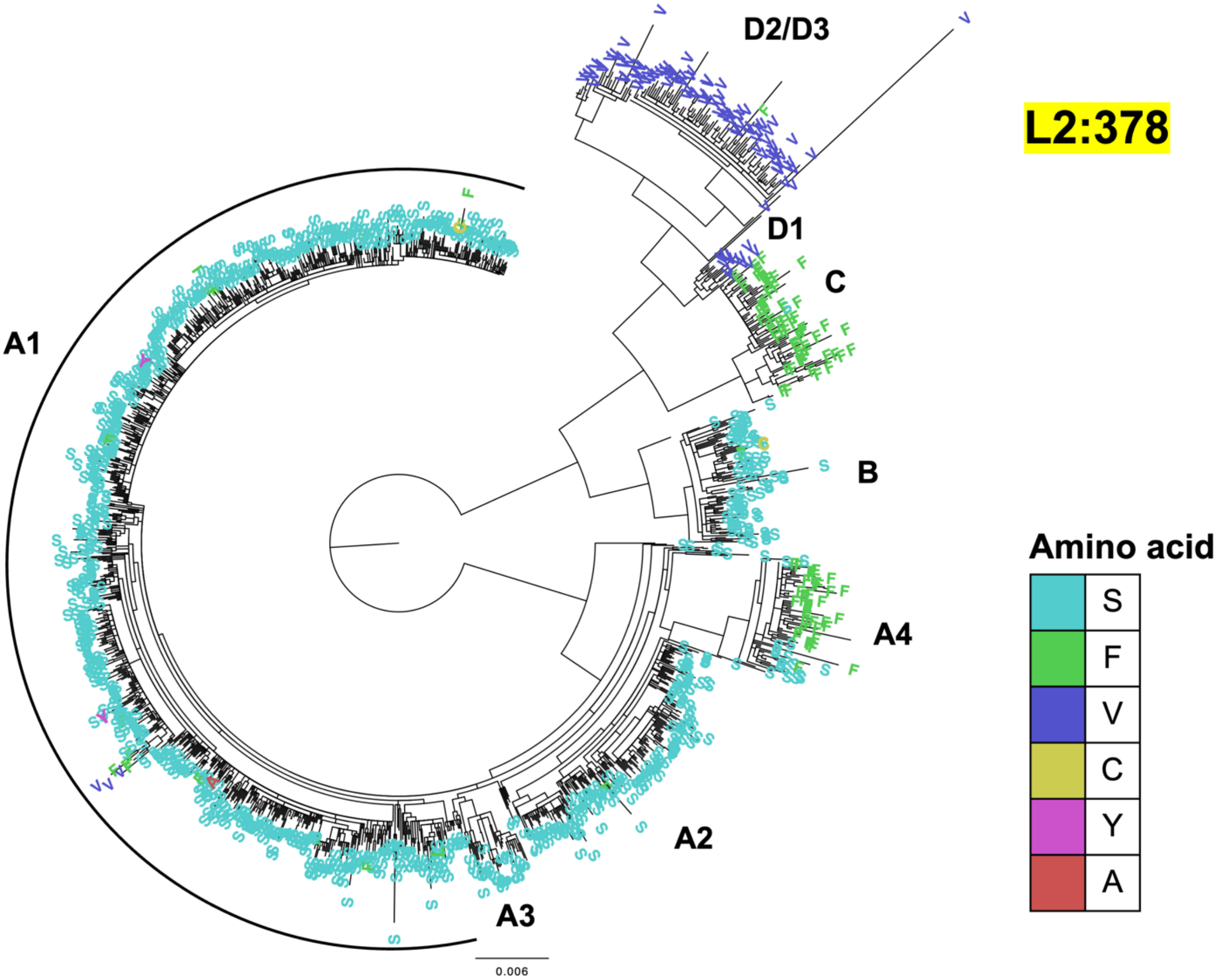

Supplementary Figure S11. HPV16 L2:378 amino acid variation and convergence. All other details as in Suppl. Fig. S10.

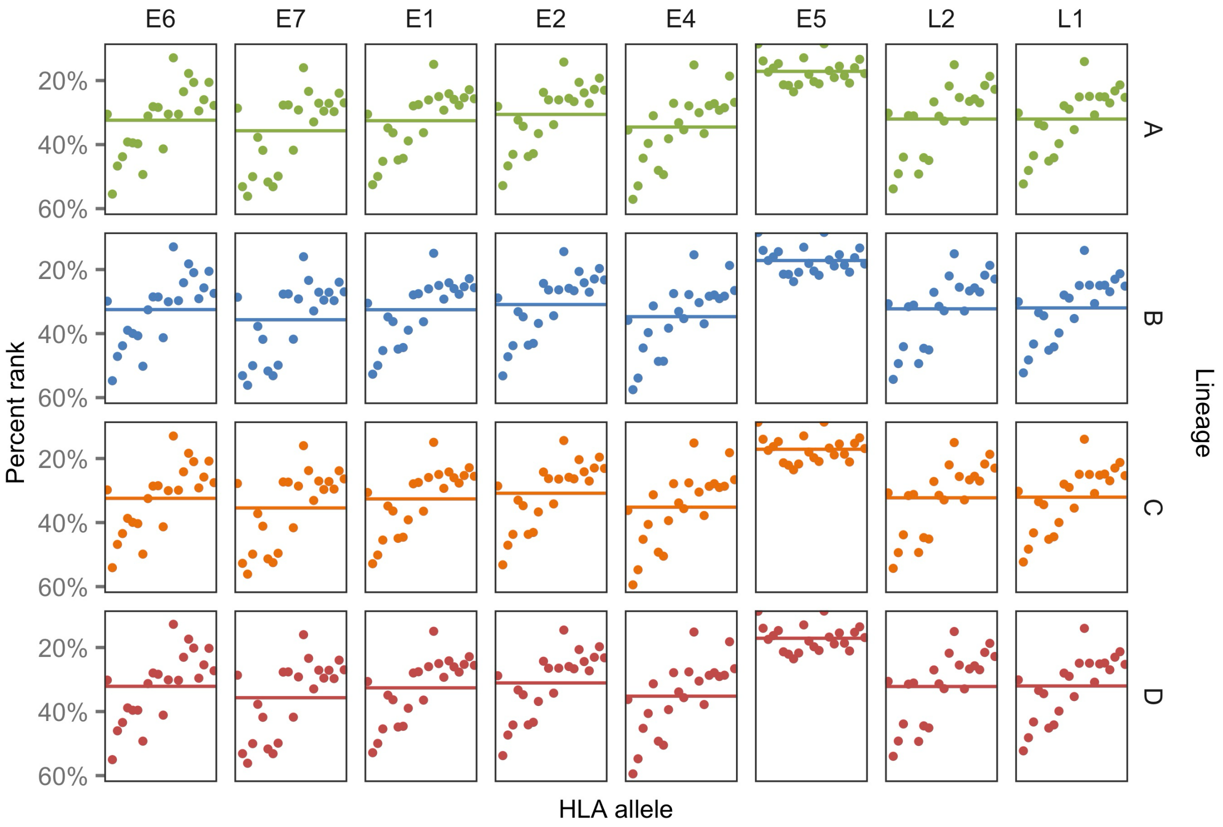

Supplementary Figure S12. Predicted MHC class I binding strength for HPV16 proteins. All nonamers (9-mer amino acid substrings) of the most common protein variant of each HPV16 lineage (A, B, C, and D) were queried using NetMHCpan-4.1[5]. Each dot represents one of 22 common HLA alleles, ordered alphanumerically (x axis): HLA-A*01:01, A*02:01, A*03:01, A*24:02, A*26:01, B*07:02, B*08:01, B*15:01, B*27:05, B*39:01, B*40:01, B*58:01, C*03:03, C*04:01, C*05:01, C*06:02, C*07:01, C*07:02, C*08:02, C*12:03, C*14:02, and C*15:02. Percent rank (%rank) corresponds to mean predicted binding strength for all 9-mers of a given protein against one HLA allele (one dot, one HLA allele), with lower values indicating stronger predicted binding (inverted y axis; higher is stronger binding). Horizontal lines show the mean percent rank across all alleles for a given protein/lineage.

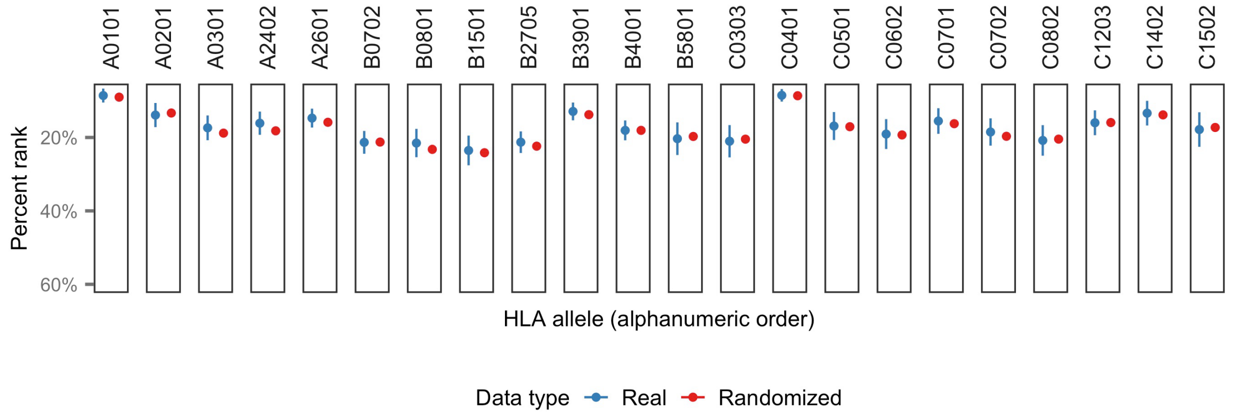

Supplementary Figure S13. Observed and expected MHC class I predicted binding strength for the HPV16 E5 protein as a function of HLA class I allele. Observed values (blue) refer to the HPV16REF (A1) variant of E5; expected (red) refer to 1000 randomized E5 nonamers (9-mer amino acid strings, formed by drawing individual amino acids from E5 with replacement).

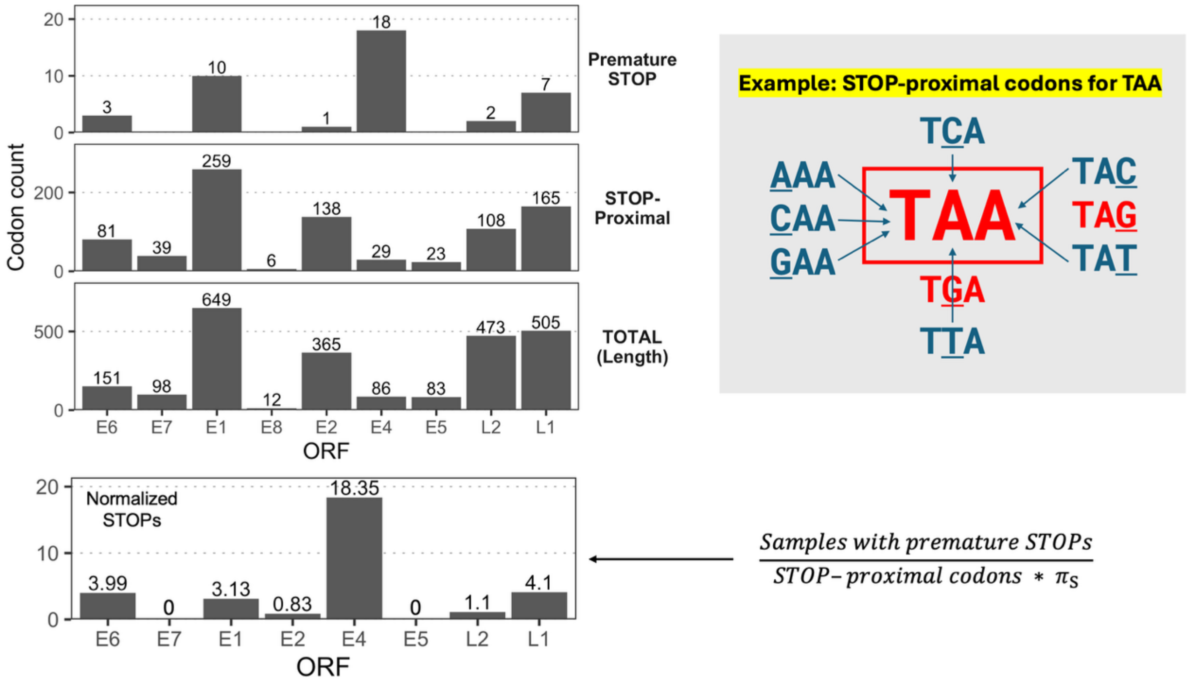

Supplementary Figure S14. E4 is enriched for premature STOP mutations. Of 4745 quality control-filtered samples, 41 have a premature STOP mutation in exactly one ORF (none have a premature STOP in multiple ORFs). STOP rates varied significantly across ORFs ($P < 0.001$; Poisson GLM, likelihood ratio test, omnibus). Normalizing further by E4's π_S (0.0338) yields a value of 18.35 (bottom).

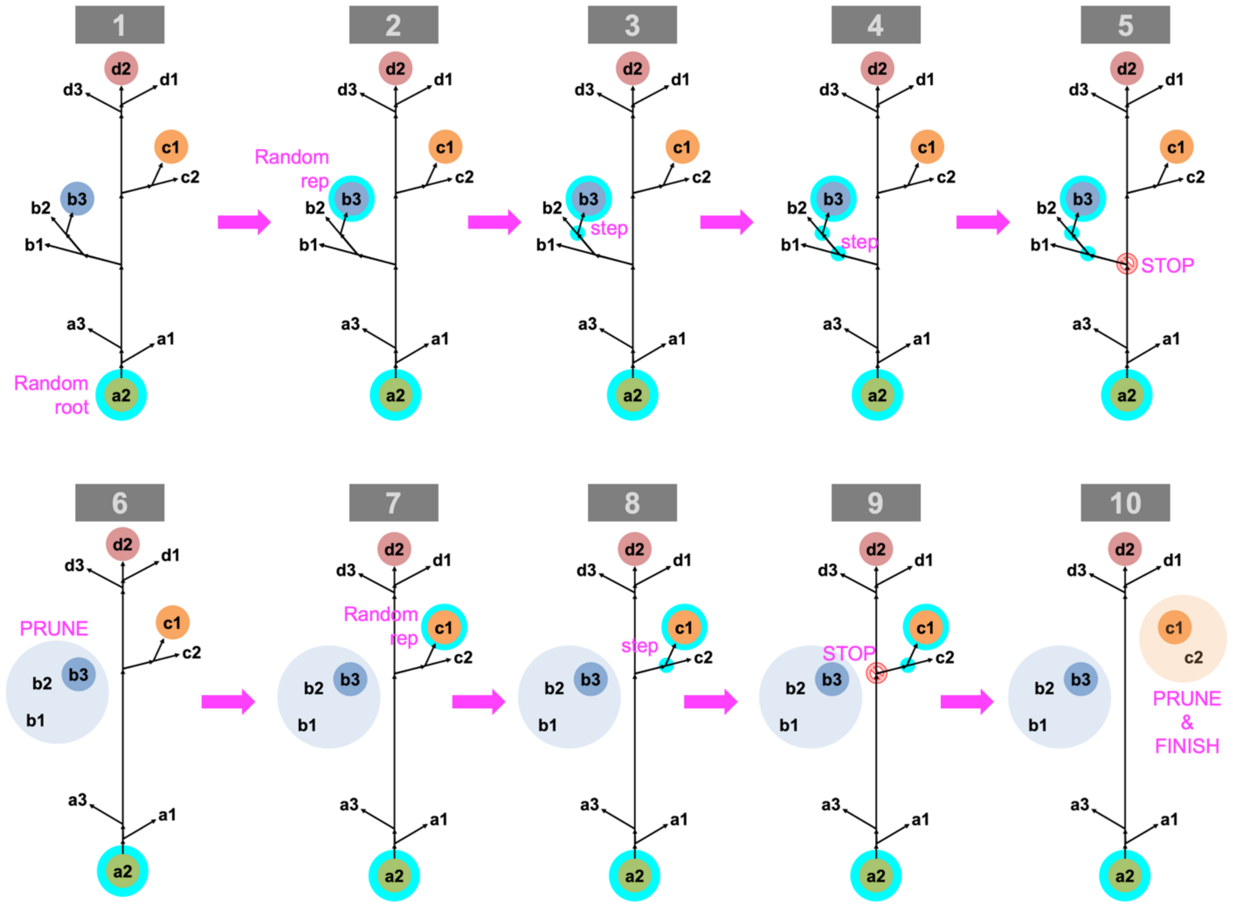

Supplementary Figure S15. One full replicate of the clade assignment algorithm. Clade representatives are provided by the user, shown here as tree leaves with color-filled circles. The algorithm begins by randomly selecting a representative at which to root, here leaf a2 (step 1). Second, it randomly selects another representative (step 2) and steps node-by-node towards the root (steps 3–4) until a node is reached from which more than one representative descends (step 5). At this point one step backward is taken, the subtree is pruned, and all descendants thereof are classified as members of that representative's clade (step 6; members of B are classified). This process is repeated until only two representatives remain (steps 7–10). Note that the number of steps will vary widely depending on the number of leaves in the tree and which random selections are made.

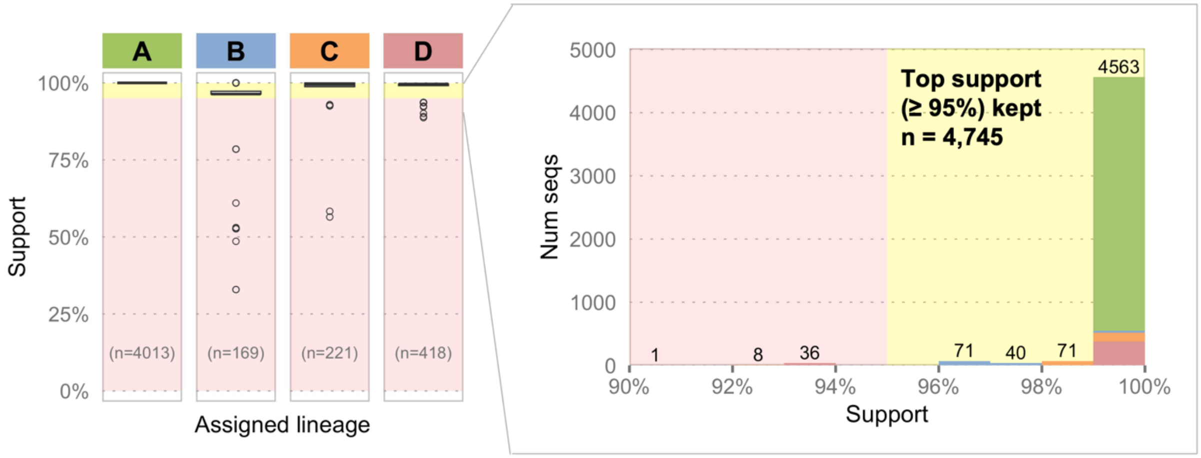

Supplementary Figure S16. Lineage assignment confidence. Numbers (n) of whole-genome sequences preliminarily assigned to each lineage were: A = 4013, B = 169, C = 221, and D = 418. Most sequences have high lineage support, with all outliers falling below 95% confidence. The 4745 sequences with high confidence (>95%) were retained for further analysis.

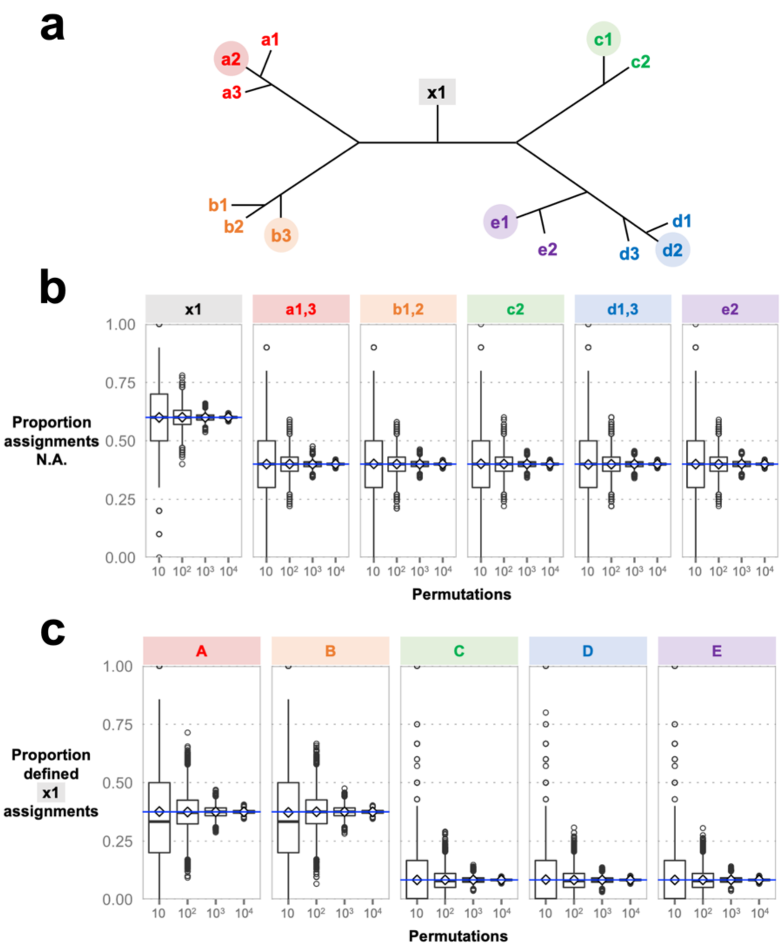

Supplementary Figure S17. Clade assignment algorithm: benchmarking with simulation. Simulations classifying each sequence for five clades were carried out with 10, 100, 1,000, and 10,000 replicates for the sample tree. **a** Sample tree with five clades (a, b, c, d, e) and a single sample (x1) not belonging to any clade. **b** Proportion of replicates in which a sample was not assigned (NA) for members of each clade. Trials revealed that 1000 replicates were sufficient to yield unambiguous results: samples that truly do not belong to any clade (sample x1) were always assigned as 'unclassified' in >50% of replicates, whereas true members were always assigned as 'unclassified' in <50% of replicates. **c** Proportion of replicates in which a true non-member sequence (x1) is classified as belonging to a clade. Non-members always yielded <50% assignment confidence, whereas true members always yielded 100% confidence. See supplementary script `execute_clade_assign_replicates.py`.

3. Tables

Supplementary Table S1. Protein-coding open reading frames of HPV16. Genome coordinates of all protein-coding ORFs in GTF format, adapted from PaVE.

<seqname>	<source>	<feature>	<start>	<end>	<score>	<strand>	<frame>	[attributes][a]
HPV16REF	PaVE	CDS	104	559	.	+	2	gene_id "E6";
HPV16REF	PaVE	CDS	562	858	.	+	1	gene_id "E7";
HPV16REF	PaVE	CDS	865	2814	.	+	1	gene_id "E1";
HPV16REF	PaVE	CDS	1265	1300	.	+	2	gene_id "E8";
HPV16REF	PaVE	CDS	2756	3853	.	+	2	gene_id "E2";
HPV16REF	PaVE	CDS	3360	3620	.	+	3	gene_id "E4";
HPV16REF	PaVE	CDS	3850	4101	.	+	1	gene_id "E5";
HPV16REF	PaVE	CDS	4237	5658	.	+	1	gene_id "L2";
HPV16REF	PaVE	CDS	5639	7156	.	+	2	gene_id "L1";

[a]E8 and E4 are spliced mid-codon, and the incomplete codon was removed to yield the largest possible number of complete codons. These records are nonredundant in that each distinct codon was included just once.

Supplementary Table S2. HPV16 codons for which evidence of positive selection has been reported in a previous study.

ORF	codon	DeFilippis et al. 2002[19]	Chen et al. 2005[20]	Carvajal-Rodríguez 2008[21]	Pimenoff et al. 2016[2]
E6	10	YES	YES	YES	YES
E6	14	YES	YES	YES	YES
E6	24	no	no	no	YES
E6	25	no	no	no	YES
E6	27	YES	no	no	YES
E6	78	no	no	no	YES
E6	83[a]	YES	YES	YES	YES
E6	113	no	no	no	YES
E7	4	no	no	no	YES
E7	28	no	no	no	YES
E7	29	no	no	no	YES
E7	30	no	no	no	YES
E1	35	no	no	no	YES
E1	120	no	no	no	YES
E1	131	no	no	no	YES
E1	144	no	no	no	YES
E1	209	no	no	no	YES
E1	241	no	no	no	YES
E1	469	no	no	no	YES
E1	483	no	no	no	YES
E1	512	no	no	no	YES
E5	48[b]	no	YES	no	YES
E5	65[b]	no	YES	no	YES
L2	269	no	no	no	YES
L2	378	no	no	YES	no
L1	174	no	no	no	YES

[a]T350C; often reported as E6 position 90. [b]E5 positions were inferred to be off by -2 in Pimenoff et al. 2016[2].

4. Data file descriptions

Supplementary Data 1. HPV16 codon metadata. HPV16 codon metadata by sub/lineage.

Supplementary Data 2. Sub/lineage-defining-sites. The major (consensus; most common) nucleotide at each of the 158 sub/lineage-defining sites for the six HPV16 sub/lineages.

Supplementary Data 3. Molecular convergence. All 7906 sites in the HPV16 genome, whether they exhibit homoplasy (any allele frequency) in the majority of five plausible trees, and whether they are sub/lineage-defining.

Supplementary Data 4. Positively selected codons. Information about each of the 56 codons with evidence for positive selection; P values refer to likelihood ratio tests (LRTs) from HyPhy-FEL (two-sided) and -MEME (one-sided) with no correction for multiple comparisons, the minimum taken from the median of 5 replicates each.

Supplementary Data 5. E1 variants and protein structure. Information about the effects on protein structure of amino acid variants at the positively selected positions in E1; P values refer to Fisher's exact tests (two-sided).

Supplementary Data 6. IEDB epitopes: initial set. Initial set of 218 unique CTL HPV16 epitope peptides from the Immune Epitope Database (IEDB) after processing, joining, and mapping of peptides to the HPV16REF protein-coding coordinates (see Suppl. Table S1).

Supplementary Data 7. IEDB epitopes: final set. Final set of 181 unique CTL HPV16 epitope peptides from the IEDB, after length and indel filtering, and including distance measures between each peptide epitope and the protein variants encoded by each sublineage.

Supplementary Data 8. IEDB selection associations. Associations between IEDB epitopes and positively selected codons by restricting HLA class I allele/serotype.

Supplementary Data 9. Sublineage reference genomes. Reference genomes used for the 16 sublineages of HPV16.

Supplementary Data 10. Masked sites. Single-column list of HPV16 genome sites that are constitutively masked our dataset alignment.

Supplementary Data 11. Top protein-variant haplotypes. The most common protein-variant haplotype observed in each of the sublineages, limiting to those with the lowest X (undefined/masked) content.

Supplementary Data 12. Centrality. Second order degree centrality (SODC) and predicted Local Distance Difference Test (pLDDT) for each amino of the HPV16REF (A1) protein variant structure.

5. References

1. Morel, B. *et al.* Phylogenetic Analysis of SARS-CoV-2 Data Is Difficult. *Molecular Biology and Evolution* **38**, 1777–1791 (2021).
2. Pimenoff, V. N., de Oliveira, C. M. & Bravo, I. G. Transmission between Archaic and Modern Human Ancestors during the Evolution of the Oncogenic Human Papillomavirus 16. *Molecular Biology and Evolution* **34**, 4–19 (2017).
3. Chen, Z. *et al.* Niche adaptation and viral transmission of human papillomaviruses from archaic hominins to modern humans. *PLoS Pathog* **14**, e1007352 (2018).
4. Huerta-Cepas, J., Serra, F. & Bork, P. ETE 3: Reconstruction, Analysis, and Visualization of Phylogenomic Data. *Mol Biol Evol* **33**, 1635–1638 (2016).
5. Reynisson, B., Alvarez, B., Paul, S., Peters, B. & Nielsen, M. NetMHCpan-4.1 and NetMHCIIpan-4.0: improved predictions of MHC antigen presentation by concurrent motif deconvolution and integration of MS MHC eluted ligand data. *Nucleic Acids Research* **48**, W449–W454 (2020).
6. Grifoni, A. *et al.* A Sequence Homology and Bioinformatic Approach Can Predict Candidate Targets for Immune Responses to SARS-CoV-2. *Cell Host & Microbe* **27**, 671-680.e2 (2020).
7. Yu, L., Majerciak, V. & Zheng, Z.-M. HPV16 and HPV18 Genome Structure, Expression, and Post-Transcriptional Regulation. *IJMS* **23**, 4943 (2022).
8. Doorbar, J. The E4 protein; structure, function and patterns of expression. *Virology* **445**, 80–98 (2013).
9. R Core Team. R: A Language and Environment for Statistical Computing. R Foundation for Statistical Computing (2024).
10. Pagès, H. BSgenome. Bioconductor, https://doi.org/10.18129/B9.BIOC.BSGENOME (2017).
11. Pagès, H. & Aboyoun, P. Biostrings. Bioconductor, https://doi.org/10.18129/B9.BIOC.BIOSTRINGS (2017).
12. Aboyoun, P. & Gentleman, R. pwalign: Perform pairwise sequence alignments. Bioconductor, https://doi.org/10.18129/B9.bioc.pwalign (2024).
13. Tamura, K., Stecher, G. & Kumar, S. MEGA11: Molecular Evolutionary Genetics Analysis Version 11. *Molecular Biology and Evolution* **38**, 3022–3027 (2021).
14. Stecher, G., Tamura, K. & Kumar, S. Molecular Evolutionary Genetics Analysis (MEGA) for macOS. *Molecular Biology and Evolution* **37**, 1237–1239 (2020).
15. Sanderson, T. Taxonium, a web-based tool for exploring large phylogenetic trees. *eLife* **11**, e82392 (2022).
16. Jumper, J. *et al.* Highly accurate protein structure prediction with AlphaFold. *Nature* **596**, 583–589 (2021).
17. Martin, D. P. *et al.* The emergence and ongoing convergent evolution of the SARS-CoV-2 N501Y lineages. *Cell* **184**, 5189-5200.e7 (2021).
18. Vita, R. *et al.* The Immune Epitope Database (IEDB): 2018 update. *Nucleic Acids Research* **47**, D339–D343 (2019).
19. DeFilippis, V. R., Ayala, F. J. & Villarreal, L. P. Evidence of Diversifying Selection in Human Papillomavirus Type 16 E6 But Not E7 Oncogenes. *Journal of Molecular Evolution* **55**, 491–499 (2002).

20. Chen, Z. *et al.* Diversifying Selection in Human Papillomavirus Type 16 Lineages Based on Complete Genome Analyses. *J Virol* **79**, 7014–7023 (2005).
21. Carvajal-Rodríguez, A. Detecting recombination and diversifying selection in human alpha-papillomavirus. *Infection, Genetics and Evolution* **8**, 689–692 (2008).
