# Supplementary material for: HPV16 genetic variation provides evidence of positive natural selection driven by HLA class I
Source: Nat Commun. 2026 Jun 2;17:7064. doi: 10.1038/s41467-026-73531-0 (PMC13392105; doi:10.1038/s41467-026-73531-0)
Supplement: Supplementary file 2 — Description of Additional Supplementary Files [file 41467_2026_73531_MOESM2_ESM.pdf]

Title: Supplementary Data 1
Description: HPV16 codon metadata. HPV16 codon metadata by sub/lineage.

Title: Supplementary Data 2
Description: Sub/lineage-defining-sites. The major (consensus; most common) nucleotide at each of the 158 sub/lineage-defining sites for the six HPV16 sub/lineages.

Title: Supplementary Data 3
Description: Molecular convergence. All 7906 sites in the HPV16 genome, whether they exhibit homoplasy (any allele frequency) in the majority of five plausible trees, and whether they are sub/lineage-defining.

Title: Supplementary Data 4
Description: Positively selected codons. Information about each of the 56 codons with evidence for positive selection; P values refer to likelihood ratio tests (LRTs) from HyPhy FEL (two-sided) and -MEME (one-sided) with no correction for multiple comparisons, the minimum taken from the median of 5 replicates each.

Title: Supplementary Data 5
Description: E1 variants and protein structure. Information about the effects on protein structure of amino acid variants at the positively selected positions in E1; P values refer to Fisher's exact tests (two-sided).

Title: Supplementary Data 6
Description: IEDB epitopes: initial set. Initial set of 218 unique CTL HPV16 epitope peptides from the Immune Epitope Database (IEDB) after processing, joining, and mapping of peptides to the HPV16REF protein-coding coordinates (see Suppl. Table S1).

Title: Supplementary Data 7
Description: IEDB epitopes: final set. Final set of 181 unique CTL HPV16 epitope peptides from the IEDB, after length and indel filtering, and including distance measures between each peptide epitope and the protein variants encoded by each sublineage.

Title: Supplementary Data 8
Description: IEDB selection associations. Associations between IEDB epitopes and positively selected codons by restricting HLA class I allele/serotype.

Title: Supplementary Data 9
Description: Sublineage reference genomes. Reference genomes used for the 16 sublineages of HPV16.

Title: Supplementary Data 10
Description: Masked sites. Single-column list of HPV16 genome sites that are constitutively masked our dataset alignment.

Title: Supplementary Data 11
Description: Top protein-variant haplotypes. The most common protein-variant haplotype observed in each of the sublineages, limiting to those with the lowest X (undefined/masked) content.

Title: Supplementary Data 12
Description: Centrality. Second order degree centrality (SODC) and predicted Local Distance Difference Test (pLDDT) for each amino of the HPV16REF (A1) protein variant structure.
