# Supplementary material for: HPV16 genetic variation provides evidence of positive natural selection driven by HLA class I
Source: Nat Commun. 2026 Jun 2;17:7064. doi: 10.1038/s41467-026-73531-0 (PMC13392105; doi:10.1038/s41467-026-73531-0)
Supplement: Supplementary file 15 — Reporting Summary [file 41467_2026_73531_MOESM15_ESM.pdf]

nature portfolio

Corresponding author(s): Chase W. Nelson, Lisa Mirabello

Last updated by author(s): Mar 18, 2026

Reporting Summary

Nature Portfolio wishes to improve the reproducibility of the work that we publish. This form provides structure for consistency and transparency in reporting. For further information on Nature Portfolio policies, see our Editorial Policies and the Editorial Policy Checklist.

Statistics

For all statistical analyses, confirm that the following items are present in the figure legend, table legend, main text, or Methods section.

n/a	Confirmed	
☐	☒	The exact sample size (n) for each experimental group/condition, given as a discrete number and unit of measurement
☐	☒	A statement on whether measurements were taken from distinct samples or whether the same sample was measured repeatedly
☐	☒	The statistical test(s) used AND whether they are one- or two-sided<br>*Only common tests should be described solely by name; describe more complex techniques in the Methods section.*
☐	☒	A description of all covariates tested
☐	☒	A description of any assumptions or corrections, such as tests of normality and adjustment for multiple comparisons
☐	☒	A full description of the statistical parameters including central tendency (e.g. means) or other basic estimates (e.g. regression coefficient) AND variation (e.g. standard deviation) or associated estimates of uncertainty (e.g. confidence intervals)
☐	☒	For null hypothesis testing, the test statistic (e.g. F, t, r) with confidence intervals, effect sizes, degrees of freedom and P value noted<br>*Give P values as exact values whenever suitable.*
☒	☐	For Bayesian analysis, information on the choice of priors and Markov chain Monte Carlo settings
☒	☐	For hierarchical and complex designs, identification of the appropriate level for tests and full reporting of outcomes
☐	☒	Estimates of effect sizes (e.g. Cohen's d, Pearson's r), indicating how they were calculated

Our web collection on statistics for biologists contains articles on many of the points above.

Software and code

Policy information about availability of computer code

Data collection	Data were obtained using Unix wget and manual download using Google Chrome.
Data analysis	Thermo Fisher Life Sciences' Ion Torrent Proton was used to amplify the entire 7906 bp HPV16 genome with a custom HPV16 Ion Ampliseq panel of 47 multiplexed primer sets (Cullen et al., 2015). Torrent Suite software (Thermo Fisher Scientific, Waltham, MA, USA) was used for sequence read quality assessment and trimming, followed by mapping to the HPV16 reference genome using the Torrent Mapping Alignment Program v. 5.0.13. Torrent Variant Caller v. 5.0.3 (Thermo Fisher Scientific) was used to call within- and between-host single nucleotide variants, and variants were annotated with SnpEff v. 3.6c and SNPGenie v. 1.0. BBEdit v. 15.5.5 was used to inspect and edit all text files. MAFFT v. 7.511 and 7.520 were used to align nucleotide sequences. MEGA 11 and AliView v. 1.28 were used to inspect and edit alignments. Pythia v. 1.1.2 was used to quantify phylogenetic signal. RAxML-NG v. 1.0.0 was used to infer maximum likelihood phylogenetic trees. FigTree v. 1.4.4 and Taxonium v. 2 were used to visualize phylogenetic trees. SNPGenie v. 1.0 was used to estimate nucleotide diversity of all genome positions. OLGenie v. 1.0.0 was used to estimate nucleotide diversity of overlapping reading frame positions. HyPhy models FEL v. 2.5 and MEME v. 4.0 were used to estimate dN/dS at individual codons. NetMHCpan-4.1 was used to predict the 9-mer cytotoxic-T-lymphocyte epitope content of all protein variants. AlphaFold2 was used to predict monomeric three-dimensional protein structures. AlphaPickle v. 1.4.1 was used to assess residue-level confidence of protein structure predictions. PyMOL was used to superimpose protein structures. DUET and DynaMut2 were used to predict the protein-stability-changing effects of amino acid variants. All subsequent data analyses and statistics were performed using Microsoft Excel, Perl v. 3.12.2, Python v. 3.12.2 / PyCharm CE v. 17.0.7, and R version 4.4.1 / RStudio v. 2025.05.1 (base R, Biostrings, boot, BSGenome, corrplot, data.table, dplyr, feather, GenomicFeatures, GenomicRanges, ggrepel, ggseqlogo, gridExtra, jsonlite, patchwork, pwalign, RColorBrewer, rtracklayer, scales, seqinr, stringdist, and tidyverse). All custom code is available at GitHub (https://github.com/chasewnelson/HPV16-molecular-evolution).

For manuscripts utilizing custom algorithms or software that are central to the research but not yet described in published literature, software must be made available to editors and reviewers. We strongly encourage code deposition in a community repository (e.g. GitHub). See the Nature Portfolio guidelines for submitting code & software for further information.

Data

Policy information about availability of data

All manuscripts must include a data availability statement. This statement should provide the following information, where applicable:

- Accession codes, unique identifiers, or web links for publicly available datasets
- A description of any restrictions on data availability
- For clinical datasets or third party data, please ensure that the statement adheres to our policy

Sequence data are publicly available and were obtained from the previous studies cited in the main text (Mirabello et al. 2016, 2017; GenBank accessions MG847621-MG850835). Source data are provided in the Supplementary Information of this paper.

Research involving human participants, their data, or biological material

Policy information about studies with human participants or human data. See also policy information about sex, gender (identity/presentation), and sexual orientation and race, ethnicity and racism.

Reporting on sex and gender	Our study included only females (cervical samples).
Reporting on race, ethnicity, or other socially relevant groupings	Samples from the Kaiser Permanente Northern California (KPNC)-National Cancer Institute (NCI) HPV Persistence and Progression (PaP) cohort and the NCI Study to Understand Cervical Cancer Early Endpoints and Determinants (SUCCEED) included self-reported race and ethnicity from electronic health records; samples from the International Agency for Research on Cancer (IARC) included region of origin. For PaP, participants self-reported as White (52%), Hispanic (20%), Asian/Pacific Islander (13%), African American (6%), Multi-racial/Other (<1%), or Unknown/NA (9%). For SUCCEED, participants self-reported as White (44%), Indian (3%), Black (3%), Multiracial (2%), Hispanic (2%), Asian (<1%), or Unknown/NA (45%). For IARC, participants were located in E Asia (24%), S Asia (20%), Latin America (18%), Africa (13%), Europe (11%), N Africa (9%), Oceania (3%), or North America (2%). Note that these strata were not used for analyses; our study focused on viral rather than host evolution and benefited from the inclusion of maximum diversity, therefore all data were pooled and instead stratified by viral sub/lineages, which differ in prevalence among human populations in different geographic regions.
Population characteristics	A total of 4704 women were included in our analyses: 2773 from PaP, 589 from SUCCEED, and 1342 from IARC. For PaP (dataset used for case/control analyses), there were 850 controls (cervical intraepithelial neoplasia [CIN] grade 1 or lower), 782 CIN grade 2 (CIN2), 1047 precancers including CIN grade 3 (CIN3) and adenocarcinoma in situ (AIS), and 94 cancers; mean age was 37 years (SD 13). For SUCCEED, there were 149 <CIN2, 134 CIN2, 182 CIN3, and 57 cancer samples; mean age was 30 (SD 11). IARC included 252 non-cancers, 535 cancers, and 7 unknown status samples; mean age was 44 (SD 14). See "Reporting on race..." above for self-reported race/ethnicity and region of origin information.
Recruitment	The PaP cohort includes approximately 55,000 out of approximately 1 million women who underwent routine cervical cancer screening between December 2006 and January 2011 at KPNC. Participants could opt-out of retaining residual cervical specimens from Pap smears and those samples were discarded (~8% of women opted out). Women were followed over time and we obtained coded information on subsequent cervical cancer screening test results and histology results from electronic health records through 2019. Cervical cell samples were collected from women positive with HPV16, including all available precancer/cancer cases and approximately 1 control per case randomly selected for comparison. For SUCCEED, women were referred to colposcopy or treatment at the University of Oklahoma Dysplasia Clinic based at the University of Oklahoma Health Sciences Centre (OUHSC), after a recent abnormal Pap smear diagnosis or a biopsy diagnosis of CIN/cancer. For IARC, samples from women in the IARC biobank were collected as part of the IARC-coordinated cervical cancer case series, cervical cancer case/control studies, and population-based HPV prevalence surveys from 39 countries worldwide.
Ethics oversight	For PaP, the KPNC Institutional Review Board (IRB) approved use of the data, and the National Institutes of Health (NIH) Office of Human Subjects Research deemed this study exempt from IRB review. For SUCCEED, written informed consent was obtained from all women enrolled in the study and IRB approval was provided by OUHSC and the US National Cancer Institute. For IARC, both local and IARC ethical committees approved all studies.

Note that full information on the approval of the study protocol must also be provided in the manuscript.

Field-specific reporting

Please select the one below that is the best fit for your research. If you are not sure, read the appropriate sections before making your selection.

☒ Life sciences ☐ Behavioural & social sciences ☐ Ecological, evolutionary & environmental sciences

For a reference copy of the document with all sections, see nature.com/documents/nr-reporting-summary-flat.pdf

Life sciences study design

All studies must disclose on these points even when the disclosure is negative.

Sample size	A total of 4704 women were included in our study: 2773 from PaP, 589 from SUCCEED; and 1342 from IARC (see "Population characteristics" above). We included all available HPV16-positive precancer (CIN3, AIS) and cancer (SCC, ADC, cancer) samples from these three large projects because these are rare outcomes of HPV infection, and the available matched non-cancer samples. For our case/control analysis using the PaP cohort, approximately one control was randomly selected per precancer/cancer case. Only one sample per woman was included. We analyzed

only the HPV16 whole genome sequences that met our strict quality-control criteria (see "Data exclusions" below).

Data exclusions	We only included samples from women that were HPV16-positive with the infection outcomes as noted per study. From an initial 6411 initial HPV16 whole genome sequences, we sequentially excluded: samples with previously-documented within-HPV16 coinfection, poor sequence quality, or incomplete metadata (359 excluded, leaving 6052); samples with <70% genome coverage after masking low-coverage (<10) sites (173 additional exclusions, leaving 5879); samples with high within-host polymorphism, considered to be intermediate VAF (variant allele fraction) values of 40–60% at >1 genome position (1029 additional exclusions, leaving 4850); samples with lineage assignment confidence of <95% (105 additional exclusions, leaving 4745); and samples with a premature STOP codon in any ORF (41 additional exclusions, leaving 4704 for final analysis). We did not exclude samples co-infected with other HPV types, since the presence of other types would not affect our HPV16 sequence data or evolutionary analyses.
Replication	Lineage assignment of all viral genomes was replicated 200 times (200 statistically indistinguishable maximum-likelihood phylogenetic trees); only those assignments with >95% support were kept (105 excluded). HyPhy selection analyses were each replicated five times (five statistically indistinguishable trees); only the median result (1 of 5) was kept.
Randomization	We included all available precancer and cancer cases, and randomly selected approximately one control per precancer or cancer case.
Blinding	The sequencing laboratory was blinded to case/control status.

Reporting for specific materials, systems and methods

We require information from authors about some types of materials, experimental systems and methods used in many studies. Here, indicate whether each material, system or method listed is relevant to your study. If you are not sure if a list item applies to your research, read the appropriate section before selecting a response.

Materials & experimental systems

n/a	Involved in the study	
☒	☐	Antibodies
☒	☐	Eukaryotic cell lines
☒	☐	Palaeontology and archaeology
☒	☐	Animals and other organisms
☒	☐	Clinical data
☒	☐	Dual use research of concern
☒	☐	Plants

Methods

n/a	Involved in the study	
☒	☐	ChIP-seq
☒	☐	Flow cytometry
☒	☐	MRI-based neuroimaging

Plants

Seed stocks	n/a
Novel plant genotypes	n/a
Authentication	n/a
