# Supplementary material for: HPV16 genetic variation provides evidence of positive natural selection driven by HLA class I
Source: Nat Commun. 2026 Jun 2;17:7064. doi: 10.1038/s41467-026-73531-0 (PMC13392105; doi:10.1038/s41467-026-73531-0)
Supplement: Supplementary file 16 — Transparent Peer Review file [file 41467_2026_73531_MOESM16_ESM.pdf]

nature portfolio

Peer Review File

HPV16 genetic variation provides evidence of positive natural selection driven by HLA class I

Corresponding Author: Dr Chase Nelson

This file contains all reviewer reports in order by version, followed by all author rebuttals in order by version.

Version 0:

Reviewer comments:

Reviewer #1

(Remarks to the Author)
Nelson et al. examine 4704 HPV16 genomes to identify viral codons under selection pressure that may result from immune recognition by the host. The manuscript is clear and very well written.

Major comments:
"Controls were defined as women with HPV16-positive samples that subsequently cleared infection or had benign infection defined as <CIN2 that did not progress to CIN2 or higher throughout the study. Cases were defined as women who were HPV16-positive with CIN3, precancer, or cancer."
"We compared 718 controls (less than cervical intraepithelial neoplasia [CIN] grade 2 [<CIN2]) and 854 cervical precancer/cancer cases (CIN grade 3 and cancer [CIN3+]) that had viruses within the A1/A2 sublineages from the PaP study".

1. After which time point were the samples determined to be consistently HPV positive/negative? Were there multiple samples from the same patient in this cohort? Were consecutively lesion-positive samples considered to be cases? (see next point).

2. From these controls, how many were without dysplasia? I find the combination of lesion-negative and mildly dysplastic samples (CIN1, CIN2) slightly problematic in this comparison, since you may "dilute" your control set, with the hypothesis that in lesion-free samples, the HPV did not overcome the selection pressure, in contrast to longer-term dysplastic lesions.

3. Did each sample/patient have only 1 HPV sub-lineage prevalent, or how were the differences taken into consideration while generating the consensus per patient?

4. Were consecutive samples from the same patient analysed to determine changes in the codons?

"Limiting the analysis to positively selected codons, cases were more likely than controls to have rare (minor allele frequency <5%) nonsynonymous variants in E1 (OR = 9.34, 95%CI = 1.4–402.5; P_{Fisher} = 0.0084) (Fig. 4b; Suppl. Fig. S6), suggesting amino acid changes at these positions were associated with oncogenesis."

5. Why is E1 picked here as an example when you have other variants with much more significant p-values that are not in E1 in this figure? Figures 4a and 4b are visually appealing but very hard to read. Please provide a (supplementary) table with detailed p-values, frequency (sample counts), ORs, and 95% CIs per variant per gene.

6. Lines 200-202. Out of 854 CIN3/cancers, how many samples had this rare NS variant? Or are these multiple rare variants? How many common variants were detected, and what is their association outcome?

7. An association with "oncogenesis" is slightly overreaching; perhaps it may simply improve viral persistence? Since the same patient may have multiple variants of the HPV strain, it is challenging to pinpoint which (or all) variants are associated. In any case, higher sample numbers would be needed for rarer variants, or multiple samples from the same patient, or a validation cohort. I would suggest toning down this statement.

8. "In contrast, no association was observed for the known protective allele B*15:01 (Fig. 5e), which had five epitopes." Is B*15:01 the only known protective allele for cervical cancer? Was HLA-C not investigated? (Please check Leo et al. (citation 35), Figure 3) or Bowden et al. (citation 36).

9. In the discussion, you state that you did not analyse within-host variation per sample- this is a major drawback of this study. Were the hosts only infected with HPV16? How were other HPV types excluded?

Minor comments:
1. Lines 94-96: "the most consistently identified locus in genome-wide association studies (GWAS) of cervical precancer/cancer is the HLA region 29–36" -> only citations 32-36 are GWASs.
2. Does the ethnicity of the human samples make a difference in the HPV lineages studied here?
3. A note on how the samples were genotyped for HPV would be good to include.
4. Using NetMHCpan, why were only viral 9-mers queried?
5. "To calculate normalized STOP rates for each ORF, the number of samples with a premature STOP was divided by the number of STOP-proximal codons for each ORF (HPV16REF sequence)." Is there a citation or precedent for this?

(Remarks on code availability)

Reviewer #2

(Remarks to the Author)
The manuscript by Nelson et al. reports a comprehensive sequence analysis of HPV16 intra-type variant genomes, focusing on the presence of codons under positive selection across the entire viral genome. Although negatively selected codons predominate, 56 codons under positive selection are identified, and these are enriched for lineage-defining sites. Among the positively selected codons, E1 rare variants show a statistically stronger association with cervical precancer/cancer cases. The positively selected codons are also found located at the HPV16 CTL epitopes reported in the database.
Overall, this study provides a valuable resource for the HPV research community, but warrants further functional studies for validation.

Major comments:
(1) Regarding the set of HPV16 genome sequences analyzed in this study, the IARC collection comprises sequences gathered from around the world. As shown in Table 1, the IARC sequence data differs significantly from the other two datasets in the distribution of HPV16 variants. Thus, from the perspective of viral evolution, particularly when considering the selection process, it is not appropriate to analyze viral sequences from the United States alongside sequences from around the world.
(2) In this study, does the term "lineage-defining sites" mean "sublineage-defining sites"? Some of them seem to be found only in the A4 sublineage as shown in Figure 1. Do the authors strictly distinguish between lineage and sublineage throughout the text?
(3) Line 145: "Thus, the codons at which sublineages differed also had elevated $\pi N/\pi S$ and tended to be the most variable positions within each sublineage." The codons at which sublineages differed (sublineage-defining sites) should be conserved within each sublineage?
(4) The genetic differences between A3 and A1/2 variants are not particularly large, and the presence of A3 sublineage is ambiguous, as evidenced by the very low number of A3 variants in the PaP and SUCCEED cohorts. Therefore, including the A3 variants separately in the analysis may not be particularly meaningful.
(5) Do the E1 residues (38, 78, 100, 208, 219, 220, 294, 597) that are predicted to undergo positive selection play any functional role in the protein? How is the functional change in E1 associated with the development of cervical cancer?
(6) Regarding HLA haplotype, what is the frequency of B*07:02 within the general population in the US? Does that fit with the authors' scenario of virus escape from the host adaptive immunity?

Minor issues:
(1) Line 52: cervical cancer is the fourth most common cancer among women worldwide, not the third. Please check a recent WHO estimate: https://www.who.int/news-room/fact-sheets/detail/cervical-cancer
(2) Line 143: "These results support previous observations that the average HPV16 codon is under purifying selection." The reference papers should be cited here.

(Remarks on code availability)

Version 1:

Reviewer comments:

Reviewer #1

(Remarks to the Author)
The authors have addressed most of my concerns and substantially improved the manuscript by adding additional analyses

and supplementary information.

I have only a few minor suggestions for clarification. First, the authors state that one HPV16-positive sample per woman was analyzed and that outcomes were determined using the full follow-up period. It would be helpful to specify which time point the sequenced HPV16 genome corresponds to (e.g., baseline sample, sample closest to diagnosis, or another defined time point) and to clarify how viral clearance was defined (e.g., a single negative HPV test versus consecutive negatives).

Second, given that approximately half of the control group includes women with CIN1, it would be useful to indicate whether the key analyses are robust when restricted to lesion-free controls (normal cytology/histology), or to briefly comment on the potential impact of including CIN1 samples in the control group.

Third, the association involving rare nonsynonymous variants in E1 appears to be driven by a very small number of samples, resulting in wide confidence intervals. A brief discussion of the uncertainty associated with these rare variants, including the possibility that they may represent passenger mutations arising during prolonged infection rather than drivers of persistence or progression, would help contextualize this finding.

Finally, given the strong phylogenetic structure of HPV16 populations, it would be helpful if the authors could comment on whether the rare E1 variants observed in cases cluster within specific viral subclades. This would help clarify whether the observed association reflects independent occurrences or variation linked to underlying phylogenetic structure.

Overall, the manuscript has been substantially improved after revision, and I believe the remaining points can be addressed with minor clarification. The study will likely be of interest to readers studying HPV evolution and host–virus interactions.

(Remarks on code availability)

Reviewer #2

(Remarks to the Author)
The authors addressed all my comments and the manuscript was significantly improved. Thank you for giving me the opportunity to review this important paper.

(Remarks on code availability)

Open Access This Peer Review File is licensed under a Creative Commons Attribution 4.0 International License, which permits use, sharing, adaptation, distribution and reproduction in any medium or format, as long as you give appropriate credit to the original author(s) and the source, provide a link to the Creative Commons license, and indicate if changes were made.
In cases where reviewers are anonymous, credit should be given to 'Anonymous Referee' and the source.
The images or other third party material in this Peer Review File are included in the article’s Creative Commons license,

unless indicated otherwise in a credit line to the material. If material is not included in the article’s Creative Commons license and your intended use is not permitted by statutory regulation or exceeds the permitted use, you will need to obtain permission directly from the copyright holder.
To view a copy of this license, visit https://creativecommons.org/licenses/by/4.0/

REVIEWER COMMENTS:

Reviewer #1 (Remarks to the Author):
Nelson et al. examine 4704 HPV16 genomes to identify viral codons under selection pressure that may result from immune recognition by the host. The manuscript is clear and very well written.

> ***RESPONSE:*** *We thank the reviewer for the positive assessment and ideas to improve our study. We have revised our manuscript, highlighted all changes in yellow, and provided point-by-point responses to all comments below. This includes an exhaustive cervical precancer/cancer case vs. control analysis of all individual polymorphic amino acid positions in our HPV16 dataset, several new supporting analyses, the addition of Suppl. Tables S6 and S7, and a new Suppl. Fig. S7.*

Major comments:
"Controls were defined as women with HPV16-positive samples that subsequently cleared infection or had benign infection defined as <CIN2 that did not progress to CIN2 or higher throughout the study. Cases were defined as women who were HPV16-positive with CIN3, precancer, or cancer."
"We compared 718 controls (less than cervical intraepithelial neoplasia [CIN] grade 2 [<CIN2]) and 854 cervical precancer/cancer cases (CIN grade 3 and cancer [CIN3+]) that had viruses within the A1/A2 sublineages from the PaP study".

1. After which time point were the samples determined to be consistently HPV positive/negative? Were there multiple samples from the same patient in this cohort? Were consecutively lesion-positive samples considered to be cases? (see next point).

> ***RESPONSE:***
> *Our analysis was limited to one HPV16-positive sample per woman. We focused our analysis on long-term evolutionary change rather than short-term within-host variation. The entire follow-up period was used to determine viral clearance (i.e., a subsequent type-specific HPV negative genotyping test) or worst histologic outcome. Specifically, the Kaiser Permanente Northern California (KPNC)-NCI HPV Persistence and Progression (PaP) cohort is a longitudital study of women undergoing routine cervical cancer screening at KPNC. These individuals had multiple screening visits and HPV genotyping at each visit (details below). They were followed prospectively from study enrollment, between December 2006–January 2011, using coded information for subsequent cervical cancer screening test results, including HPV genotyping and histology results, with electronic health records. The women included in our study were followed for a median of 4.3 years (interquartile range = 3.36; range = 7.85). Several subjects were followed for 7–8 years and follow-up is still ongoing, with the last electronic health records update in 2019. This follow-up period helped ensure these were true controls, i.e., 80–90% of HPV16 infections clear and progression is a rare outcome. The HPV16-positive precancer (CIN3 or AIS) and cancer cases were diagnosed at enrollment or during the study follow-up period, and individuals were censored at a CIN2 or worse (CIN2+) diagnosis and treatment. Samples were not consecutively CIN2+ lesion-positive due to this censoring, although we expect most samples to have been consecutively HPV16 positive for multiple screening visits prior to the CIN2+ diagnosis (i.e., HPV16 persistence is an established step in progression to cervical cancer). The controls were defined*

as women having baseline specimens that were HPV16 positive and no histologic evidence of CIN2+ at enrollment or during follow-up according to the electronic health records.

SUCCEED and IARC studies were not longitudinal studies and only one patient sample was collected and evaluated per woman. For these samples, the biopsy or cervical cell sample was evaluated and determined to be HPV16 positive (see Methods below). For these precancer and cancer cases, they were diagnosed based on the tested sample and received treatment for any detected CIN2+ lesion.

To provide this and related methodological information in the main text, we added the following to the first two paragraphs of the Methods (lines 429–456): "Controls were defined as women from the PaP cohort with baseline enrollment specimens that were HPV16 positive with no histologic evidence of CIN2 or worse at enrollment or during the follow-up study period, according to the electronic health records (402 with ≤CIN1 histology and 448 with normal or no histology during our study, i.e., biopsy not indicated). These controls underwent follow-up for a median of 4.3 years (interquartile range = 3.36; range = 7.85 years) with no subsequent detection of CIN2 or higher. Cases were defined as women that were HPV16 positive and diagnosed with precancer (CIN3 or adenocarcinoma in situ*) or cancer (squamous cell carcinoma or adenocarcinoma). One sample per woman was analyzed.*

Women included from the KPNC-NCI HPV PaP cohort underwent routine cervical cancer screening using the Hybrid Capture 2 assay (HC2; Qiagen Inc, Germantown, MD) to test for a pool of 13 carcinogenic HPV types along with cytology cotesting[67]. The HC2-positive samples were HPV genotyped using Onclarity (BD, Sparks, MD), Linear Array® HPV Genotyping System (Roche Molecular Diagnostics, Pleasanton, CA), or MY09-MY11 PCR and type-specific dot-blot hybridization (Burk Laboratory, Bronx, NY)[68,69]. Women in the SUCCEED study were referred to colposcopy or treatment at the University of Oklahoma Dysplasia Clinic based at the University of Oklahoma Health Sciences Centre (OUHSC), after a recent abnormal Pap smear diagnosis or a biopsy diagnosis of CIN/cancer. SUCCEED sample HPV genotyping was done using the Linear Array® HPV Genotyping System (Roche Molecular Diagnostics)[70,71]. Hybridization of PCR products to linear arrays and subsequent signal detection were performed using the Auto-*LiPA automated staining system (Innogenetics N.V., Belgium). Hybridization to both β-globin probes was required to report genotyping results. A hybridization signal was considered positive when an unambiguous, continuous band was observed on the array. Samples from women in the IARC biobank were collected as part of the IARC-coordinated cervical cancer case series, cervical cancer case–control studies, and population-based HPV prevalence surveys from 39 countries worldwide. IARC HPV genotyping was conducted using a using a GP5+/6+-based PCR system[72] in one centralized laboratory (Department of Molecular Pathology, Vrije University, Amsterdam, The Netherlands).*

Also see our response to minor comment #3.

2. From these controls, how many were without dysplasia? I find the combination of lesion-negative and mildly dysplastic samples (CIN1, CIN2) slightly problematic in this comparison, since you may "dilute" your control set, with the hypothesis that in lesion-free samples, the HPV did not overcome the selection pressure, in contrast to longer-term dysplastic lesions.

RESPONSE:
In our study, controls are defined as women having baseline enrollment specimens that were HPV16 positive and no histologic evidence of CIN2 or worse at enrollment or during the follow-up study period according to the electronic health records. We excluded women with CIN2 from the case/control comparisons as this is an equivocal precancer lesion. Approximately half of the controls (n=448; 52.7%) had normal cytology and histology (within normal limits) or no histology (i.e., biopsy not indicated in these women) at enrollment or during follow-up, which would be the most lesion-free samples. However, the women with mild dysplasia only displayed evidence of infection or low-grade abnormal cells: 24 had ASCUS (atypical squamous cells of undetermined significance) and 378 had CIN grade 1. In addition, these women were followed for up to 8 years and none had subsequent progression to CIN2 or worse during this period, so we felt confident including them in the control group.

Additional details about the controls were added to the Methods (lines 429–456); see previous point #2.

3. Did each sample/patient have only 1 HPV sub-lineage prevalent, or how were the differences taken into consideration while generating the consensus per patient?

RESPONSE: *This is a critical point, and we limited our analyses to samples with a single sublineage of HPV16, as described in the original Methods (e.g., "We sequentially removed: samples with previously documented co-infection...", lines 475–476). Our exclusion of samples that had within-host polymorphism or ambiguous lineage assignment were further steps meant to ensure such co-infections were not present in any of our analyses.*

To draw more attention to this, we have added the following to the "Lineage assignment confidence" section of the methods (lines 518–519): "As noted above, samples with evidence of co-infection by multiple HPV16 sub/lineages were excluded".

To our knowledge, ours are the strictest criteria ever employed for excluding multi-sublineage coinfection in a study of any HPV type. Furthermore, in those samples where a single highly variable position did occur (i.e., variant allele fraction of 40–60%), we masked the position to ensure it did not contribute to the evolutionary signal of positive selection. This also served to make our detection of positive selection conservative.

4. Were consecutive samples from the same patient analysed to determine changes in the codons?

RESPONSE: *Consecutive samples from the same patient were not analyzed. Temporal/longitudinal analysis is an excellent idea that addresses the (short-term) evolution of HPV within a single patient, and is the subject of a subsequent study by our group. However, we were careful not to analyze such within-host change in the present study, because much of the viral genetic change that accumulates in a single individual over time occurs after productive infection and viral transmission has ceased. Instead, we intentionally limited our scope to the historical (long-term) evolution of the virus in humans. This was accomplished by analyzing only one representative sample per patient, and further by*

analyzing only viral genetic variants shared by two or more related samples (i.e., only those occurring on internal phylogenetic tree branches), as described in the manuscript.

To better clarify this point, we have added the following to the Methods: "One sample per woman was analyzed" (lines 436–437). The Results section as originally written also includes the point about internal phylogenetic tree branches—"Only internal tree branches were examined (Suppl. Fig. S5), thereby limiting to viral mutations that have undergone at least one round of successful transmission[44] and are likely 'circulating' in the population" (lines 186–188). This point is also further elaborated in the Methods (lines 608–614) and Supplement.

"Limiting the analysis to positively selected codons, cases were more likely than controls to have rare (minor allele frequency <5%) nonsynonymous variants in E1 (OR = 9.34, 95%CI = 1.4–402.5; PFisher = 0.0084) (Fig. 4b; Suppl. Fig. S6), suggesting amino acid changes at these positions were associated with oncogenesis."

5. Why is E1 picked here as an example when you have other variants with much more significant p-values that are not in E1 in this figure? Figures 4a and 4b are visually appealing but very hard to read. Please provide a (supplementary) table with detailed p-values, frequency (sample counts), ORs, and 95% CIs per variant per gene.

RESPONSE: *To address these questions, we have conducted one additional analysis and provided two new supplementary tables (Suppl. Tables S5 and S6).*

As an initial clarification, E1 was not an arbitrarily chosen example but rather a result of the analysis: E1 was the only ORF for which a case vs. control difference was significant at positively selected codons (even though other codons had stronger signals of positive selection, independently of status). Specifically, a ~9-fold higher proportion of cases than controls had rare nonsynonymous variants at positively selected codons in E1.

Thus, there are actually two questions being addressed: evidence of positive selection (an analysis yielding 56 individual codons) and evidence of a case vs. control difference (an analysis of 8 ORFs, collectively considering the positively selected codons within each). For the second, each ORF was classified into two sets of codons, positively selected vs. the remainder, and a case vs. control difference for rare nonsynonymous variation was evaluated specifically at the positively selected codons, of which there are eight in E1 (positions 38, 78, 100, 208, 219, 220, 294, and 597). To clarify this, we have added the words "considering positively selected codons collectively" (line 206).

First, to provide the requested details about each of the 56 positively selected codons, we now include Suppl. Table S5, which contains codon-based information on dN/dS p-values, counts of amino acids encoded, network centrality, IEDB epitopes mapped, and more. Note that these data were all previously available in Suppl. File 1, but we agree with the reviewer that a more succinct format focusing on the positively selected codons is useful. We cite this new table in the following sentence of the Results (lines 193–194): "Information about each of the 56 codons with evidence for positive selection is given in Suppl. Table S5".

Second, Figure 4b already provided the requested ORs, 95% CIs, and p values of our previous ORF-based case/control analysis. Thus, we interpreted the reviewer's comment as a request for a new case/control analysis of all amino acid positions in the HPV16 A1/A2 proteome. Note that a case/control analysis of individual SNPs had already been performed using our PaP data in a previous study (Mirabello et al. 2017, our ref. 10); this identified two SNPs in the URR (upstream regulatory region) that are associated with reduced risk of precancer/cancer (ORs ≤0.06), but no positions in protein-coding regions.

*Similar to the results of Mirabello et al. 2017, our new case/control analysis of HPV16 A1/A2 amino acid variants does not yield any significant results: there are five amino acid positions where a case/control difference has P <0.05, but none survives false-discovery rate correction (Benjamini-Hochberg) and none has evidence for positive selection (see the **Table** and **Figure** immediately below). Nevertheless, it is interesting that all of these positions have more variation in cases than controls, consistent with the signal from positively selected codons. (Note that E5:44 is shown here for completeness, but this position would have been excluded from the original analysis because it has a common variant, i.e., >5% overall frequency).*

Table: Case vs. control analysis of variation at all polymorphic amino acid positions in HPV16 sub/lineage A1/A2: top results.

Amino acid	Cases with variants	Controls with variants	Case vs. control OR (95% CI; P-value, Fisher)
L2:4	5.2% (42/804)	2.4% (16/674)	2.27 (1.23–4.36; P = 0.00470)
L2:266*	4.8% (41/853)	2.2% (16/717)	2.21 (1.20–4.26; P = 0.00661)
E1:491*	2.5% (20/811)	0.90% (6/669)	2.79 (1.07–8.55; P = 0.0274)
L2:419	0.70% (6/852)	0% (0/715)	— (0.99–Inf; P = 0.0346)
E5:44	45.3% (167/369)	36.5% (80/219)	1.44 (1.01–2.06; P = 0.0387)

*Sub/lineage-defining position

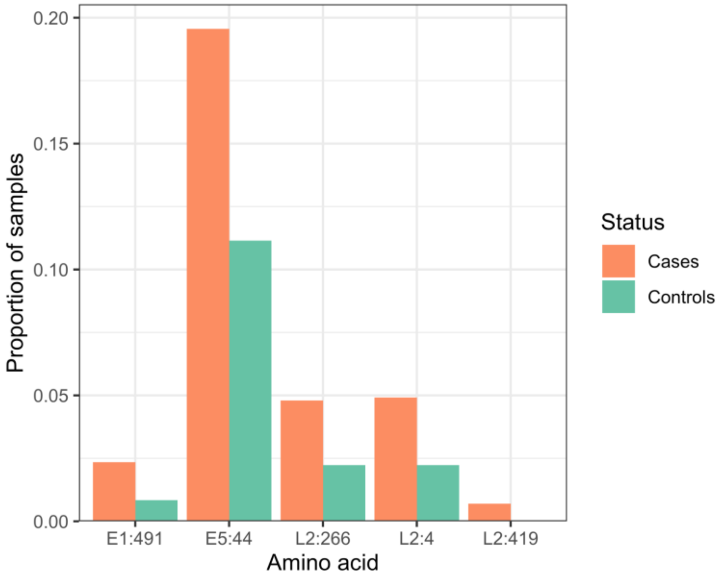

Figure: Proportion of samples with a minor variant, by case/control status.
Analysis was limited to PaP A1/A2 samples to control for the effects of sub/lineage.

Although this new analysis did not yield significant results, it does allow us to specify which of the eight E1 positively selected codons underlied the ORF's case/control difference: they were positions 38 (0.12% of cases vs. 0% controls have a variant), 100 (0.72% vs. 0.14%), 208 (0.12% vs. 0%), and 294 (0.35% vs. 0%). The remaining four positively selected positions had no variation in A1/A2 (219, 597), had variants that were not rare (220), or were inconsistent with the pattern (position 78 with 0.12% vs. 0.14%). To reflect this, we have included this information in the new Suppl. Fig. S6 and made the following modification to the Results: "Specifically, cases had more rare variation than controls at E1 positions 38 (N-terminal domain), 100 (nuclear export signal), and 208 and 294 (DNA-binding domain; UniProt ID: P03114) (Suppl. Fig. S6; Suppl. Table S6)" (lines 209–211).

Finally, regarding Figures 4a and 4b, we agree that they show a lot of information but feel this is critical to retain, as it places the positively selected codons in the context of the full viral genome and communicates the relative rarity of positive selection compared to purifying selection. It is also useful for a quick assessment of differing ORF sizes, which helps for interpreting the differing numbers of positively selected codons in each ORF (e.g., few in L1 despite its length).

6. Lines 200-202. Out of 854 CIN3/cancers, how many samples had this rare NS variant? Or are these multiple rare variants? How many common variants were detected, and what is their association outcome?

RESPONSE: *This information is important and was provided in Suppl. Fig. S6 (original text lines 202-203). There are 17 samples with rare amino acid variants at positively selected sites in E1, of which one is a control, five are CIN2, and eleven are CIN3+; this includes multiple rare variants (shown in Suppl. Fig. S6 and now also provided in the new Suppl. Table S6, noted above).*

We detected a total of 551 polymorphic amino acid positions across the A1/A2 viral genomes; of those, there were 13 (2.4%) that had a frequency >5% to be considered common. Please see the table of variants provided in the response to comment #5 above that includes the one common variant that we detected associated with outcome: E5 position 44 (OR 1.44, 95% CI 1.01–2.06; P = 0.0387). However, none of these variants were associated with outcome after correction for multiple tests.

As for the previous point, we have added the following text and reference to the new Suppl. Table 6 with this information: "Specifically, cases had more rare variation than controls at E1 positions 38 (N-terminal domain), 100 (nuclear export signal), and 208 and 294 (DNA-binding domain; UniProt ID: P03114) (Suppl. Fig. S6; Suppl. Table S6)" (lines 209–211). Note that all variant information for all positions in all sub/lineages is also available as part of Suppl. File 1.

7. An association with "oncogenesis" is slightly overreaching; perhaps it may simply improve viral persistence? Since the same patient may have multiple variants of the HPV strain, it is challenging to pinpoint which (or all) variants are associated. In any case, higher sample numbers would be needed for rarer variants, or multiple samples from the same patient, or a validation cohort. I would suggest toning down this statement.

RESPONSE: *We agree that these variants could also be improving viral persistence and appreciate the reviewer's cautionary point on this language. We have toned down this statement to read "viral persistence or progression to cancer" (line 209). Of note, we used very stringent quality control to exclude multi-sub/lineage co-infections (see Methods and response to point #3 above), which we expect to be the individuals with multiple HPV16 strains noted, in order to pinpoint the associated variants; this association analysis was additionally limited to samples with A1/A2 virus to further mitigate any influence from HPV16 sub/lineages.*

8. "In contrast, no association was observed for the known protective allele B*15:01 (Fig. 5e), which had five epitopes." Is B*15:01 the only known protective allele for cervical cancer? Was HLA-C not investigated? (Please check Leo et al. (citation 35), Figure 3) or Bowden et al. (citation 36).

RESPONSE: *The reviewer makes the important point that several MHC loci/alleles are associated with cervical disease across various studies. However, this is complicated by the fact that the alleles are not independent, due to the existence of complex linkage relationships (haplotypes) among the HLA loci. After controlling for (conditioning upon) such linkage, both Leo et al. 2017 and Bowden et al. 2021 identify robust associations only with HLA-B for HPV16.*

*Leo et al. 2017 explain in their section "HLA protective alleles": "Conditioning on HLA-B*15 completely controls for the association at HLA-C*0303 (OR = 0.80, P = 0.03), whereas conditioning on HLA-C*0303 leaves residual association at HLA-B*15 (OR = 0.7, P = 2.04 x 10^{-5}),* ***implying that the causative association is with HLA-B*15****" and "This indicates that there are separate protective associations with the HLA Class II haplotype HLA-DRB1*13/HLA-DQB1*0603/HLA-DQA1*0101,* ***and the HLA Class I allele HLA-B*15, and that other protective allelic associations are likely to be due to linkage disequilibrium*** *with these associated variants"* ***[emphasis added].***

Further, we decided to limit to the alleles Leo et al. identified as having an association specifically for HPV type 16, the subject of our own study. In their section "Associations with cervical cancer subtypes", subsection "HPV genotypes", they state that for HPV16 "The HLA Class I ***risk*** *was most strongly associated with* ***HLA-B*07:02****… [and] r****educed risk*** *was seen most strongly with* ***HLA-B*15****"* ***[emphasis added]*** *(see their Table 5, "HPV16" columns, where the P values at the A and C loci are orders of magnitude higher). Bowden et al. 2021 similarly only report robust findings for HLA-B among the HLA class I loci, along with several other non-class I loci. Thus, we remain confident in our interpretation of the literature that the HLA class I alleles most robustly associated with HPV16-related cervical disease are B*07:02 (risk) and B*15:01 (protective).*

*Finally, as a more practical matter, those HLA-A and -C alleles that did have marginal associations in Leo et al. (A*03:01, C*03:03, and C*07:02) often had insufficient numbers of epitopes in IEDB to allow us to test their associations with positive selection: there were zero C*03 epitopes, three C*07:02 epitopes, and although we were able to test A*03:01 (13 epitopes), they did not reveal any association (shown in Figure 5e).*

9. In the discussion, you state that you did not analyse within-host variation per sample- this is a major drawback of this study. Were the hosts only infected with HPV16? How were other HPV types excluded?

RESPONSE: *We did not analyze within-host variation because our aim was to examine the historical evolution of the virus (long-term) rather than its within-host change within a single patient (short-term). However, we did initially survey within-host variation with the aim of excluding samples with multi-sub/lineage co-infection (see response to comment #4 above and related additions).*

While we removed samples with multiple HPV16 sub/lineages, we did not remove samples co-infected with multiple HPV types. This will not affect our HPV16 sequence data, phylogenies, or dN/dS analyses. First, types differ by >10% in their L1 ORF, and often more in other ORFs, and their sequence data were excluded from our analyses methodologically by (1) type-specific primers and (2) read quality-control and mismatch criteria (see Cullen et al. 2015, our ref. 73). Thus, even in the presence of multi-type co-infection, the HPV16 sequence data are clean, and their phylogenies will be unaffected. Second, for dN/dS, we only considered the internal branches of the phylogenetic tree, which eliminated any changes unique to just one sample and its co-infection status (see point #4 above, Methods lines 604–614, and Suppl. Fig. S5).

With respect to persistence and progression, there is a well-known phenomenon of "one lesion, one virus" in which clonal expansion of one type occurs as others are lost. This is an inevitable part of progression. HPV16 is by far the most carcinogenic HPV type and is considered to be the 'causitive type' if multiple co-infected types are present. As such, excluding multi-type co-infections would introduce a severe bias by disproportionately excluding controls from our data. Moreover, multi-type co-infection is a common feature of HPV16's "ecology", and even those samples that are not currently co-infected (disproportionately cases) may have experienced co-infection in the past.

Given the irrelevance to our sequence data and evolutionary analyses, the potential for introduction of systematic bias, and the inability to determine which mono-infected samples were previously co-infected, we opted not to exclude multi-type co-infection, as also done in previous HPV16 studies. To make this clear to the reader, we have added the following to the Methods: "We did not exclude samples co-infected with multiple HPV types, since the presence of other types would not affect our HPV16 sequence data or evolutionary analyses, and their exclusion would systematically deplete controls" (lines 482–484).

Minor comments:

1. Lines 94-96: "the most consistently identified locus in genome-wide association studies (GWAS) of cervical precancer/cancer is the HLA region 29–36" -> only citations 32-36 are GWASs.

RESPONSE: *We thank the reviewer for catching this; we have changed the sentence to read "genome-wide (GWAS)[29–33] and targeted[34–36] genetic association studies of cervical precancer/cancer".*

2. Does the ethnicity of the human samples make a difference in the HPV lineages studied here?

RESPONSE: *Host genetic ancestry is related to the HPV16 sub/lineage distribution, as the sublineages are highly correlated with geographic location. We consider the inclusion of samples from multiple ancestries/ethnicities and geographic locations—all sequenced and analyzed identically—to be a major advantage of our study. Specifically, the phylogenetic and other evolutionary methods we employ both account for genetic relatedness of viral samples and also gain power when additional genetic variation is included. The viral genetic ancestry "tree" of each sub/lineage can be considered a statistically independent "trial" or opportunity for evolutionary variation to occur at any given site, including sub/lineage-defining sites, thus augmenting our power to detect selection.*

3. A note on how the samples were genotyped for HPV would be good to include.

RESPONSE: *We apologize for not including these methods; since these data were previously published we had simply referred to those studies and left some of this information out of the Methods. We have now added the following information to the Methods about the samples and HPV genotyping for each study on lines 429–456; see response to major comment #1 above.*

4. Using NetMHCpan, why were only viral 9-mers queried?

RESPONSE: *This is a great question, and we also considered analyzing all lengths from 8 to 11 amino acids (aa), as NetMHCpan allows CTL epitope queries of any of these lengths. However, we decided to limit this analysis to 9-mers for several related reasons. The main reason is that 9-mers are by far the most common CTL epitope length. This is true both for MHC class I binders overall, and specifically for HPV16, as recently shown by Miyauchi et al. 2023 (ref. 19) for CAL-27 cells (A*26:08 / A*26:08; B*44:03 / B*51:01; C*07:02 / C*16:01). Relatedly, as a consequence of their preponderance, 9-mers dominate the training data for NetMHCpan, such that predictions are most reliable for 9-mers, and less so for other lengths. Finally, even for lengths other than 9 aa, the NetMHCpan model identifies a 9 aa binding "core" that informs predictions. Thus, we felt it was safest to limit this analysis to 9-mers. To clarify this, we have added the following sentence to the Methods (lines 555–556): "Only 9-mers were queried because this is the most common CTL epitope length and dominates the training data of NetMHCpan".*

5. "To calculate normalized STOP rates for each ORF, the number of samples with a premature STOP was divided by the number of STOP-proximal codons for each ORF (HPV16REF sequence)." Is there a citation or precedent for this?

RESPONSE: *This normalized STOP rate is analogous to dN and dS in that a measure of diversity is being divided (normalized) by the number of sites of the relevant type. Although we are not aware of a citation for our specific method, there is precedent for estimating similar terminating-site statistics, e.g., the "dN/dS ratio for nonsense mutations" in Martincorena et al. 2017 (Cell 171:1029-1041.e21). This is appropriate because the number of sites is proportional to the number of opportunities for each mutation type. In the standard genetic code utilized by HPV16, the numbers of sites in random protein-coding regions are approximately 71% nonsynonymous (N), 25% synonymous (S), and 4% terminating*

(T = nonsense = STOP). Instead of using these global averages, we used the exact counts of STOP sites in each ORF for HPV16REF. The only difference from dN and dS is that we are using the number of samples with a STOP mutation observed, rather than the mean number of pairwise differences between all samples, which is appropriate because no sample contains more than one such mutation (multiple hits are negligible). Such normalization is critical because the ORFs differ in length and number of STOP sites, so the numbers of STOP mutations are not comparable between them without controlling for these differences.

While most site-based evolutionary analyses are focused on viable changes and species-level evolution, and therefore explicitly exclude terminating changes, analyzing them makes sense in the context of dead-end (no onward transmission) and within-host viral evolution of single samples.

Reviewer #2 (Remarks to the Author):
The manuscript by Nelson et al. reports a comprehensive sequence analysis of HPV16 intra-type variant genomes, focusing on the presence of codons under positive selection across the entire viral genome. Although negatively selected codons predominate, 56 codons under positive selection are identified, and these are enriched for lineage-defining sites. Among the positively selected codons, E1 rare variants show a statistically stronger association with cervical precancer/cancer cases. The positively selected codons are also found located at the HPV16 CTL epitopes reported in the database.
Overall, this study provides a valuable resource for the HPV research community, but warrants further functional studies for validation.

RESPONSE: *We thank the reviewer for the positive assessment and do hope the HPV community will utilize our work. We have revised our manuscript, highlighted all changes in yellow, and provided point-by-point responses to all comments below. This includes an exhaustive cervical precancer/cancer case vs. control analysis of all individual polymorphic amino acid positions in our HPV16 dataset, several new supporting analyses, the addition of Suppl. Tables S6 and S7, and a new Suppl. Fig. S7.*

Major comments:
(1) Regarding the set of HPV16 genome sequences analyzed in this study, the IARC collection comprises sequences gathered from around the world. As shown in Table 1, the IARC sequence data differs significantly from the other two datasets in the distribution of HPV16 variants. Thus, from the perspective of viral evolution, particularly when considering the selection process, it is not appropriate to analyze viral sequences from the United States alongside sequences from around the world.

RESPONSE: *This is an important question and we thought a lot about how geographic and other population substructure affects viral evolution. We used phylogenetic methods for reconstructing the ancestry relationships among the viral isolates in our dataset, because these evolutionary trees allowed us to account for genetic relatedness regardless of a sample's geographic origin. We think including multiple geographic locations is instead a strength of our study, since it increases the breadth of selection pressures that will be surveyed. Even if the same evolutionary change arises in different*

regions (or different changes in the same region), phylogenetic methods will still group the most closely related samples together (subject to signal). On one extreme, two geographically close isolates could be very distantly related (say, an A1 sequence vs. a D2/D3 sequence sampled from the same location); on the other extreme, two geographically distant isolates could be very closely related (say, two A1 sequences from distant locations, where one was carried by travel across the world before infecting someone else).

Important context, noted in the paper's Introduction (lines 60–61), is that the sublineages of HPV16 began diverging ~500 kya, such that a given A1 sequence and D2/D3 sequence last shared a common ancestor at that time. Thus, even if an A1 and D2/D3 isolate were sampled from the same human community, this would not imply they are any more closely related. By using robust phylogenetic methods, the mostly closely-related isolates were appropriately clustered in the phylogenetic tree regardless of where they were obtained.

One related issue is the differing genetic makeups of different human populations. Different populations represent distinct "environments" for the virus, including different frequencies of HLA alleles, which could exert different selective pressures on different viral variants. Thus, sampling from as wide a range of locations as possible would increase the power to detect selection in our study, by exposing the isolates under study to as many environments (hosts) as possible, without losing track of which isolates are most closely related (determined phylogenetically).

To clarify this issue, we have added the following to the Results: "These trees allowed us to determine the evolutionary relationships among isolates regardless of geographic origin" (lines 185–186).

(2) In this study, does the term "lineage-defining sites" mean "sublineage-defining sites"? Some of them seem to be found only in the A4 sublineage as shown in Figure 1. Do the authors strictly distinguish between lineage and sublineage throughout the text?

***RESPONSE:** We thank the reviewer for pointing out that our use of the term "lineage-defining" in the manuscript was confusing. We were referring to both lineage-defining and sublineage-defining sites, therefore, we have now changed the term to "sub/lineage-defining" throughout the manuscript to be more clear, and edited the text to read "A1/A2, A3, A4, B, C, and D2/D3; hereafter 'sub/lineages'" (line 125). (The separate terms 'lineage' or 'sublineage' are retained on the few occasions where they remain appropriate.) We had previously used "lineage-defining site" as a catch-all to include sites at which variants distinguish either lineages or sublineages, i.e., using 'lineage' in the evolutionary sense.*

(3) Line 145: "Thus, the codons at which sublineages differed also had elevated πN/πS and tended to be the most variable positions within each sublineage." The codons at which sublineages differed (sublineage-defining sites) should be conserved within each sublineage?

***RESPONSE:** We are pleased that the reviewer calls attention to this point, as it is a major and seemingly paradoxical observation of our study: sub/lineage-defining sites are indeed the **least conserved** within sublineages. To see how this can be so, note that sub/lineage defining sites are those at which sub/lineages differ in their major (most common) nucleotide. Critically, these genome*

positions are not necessarily fixed (100%) for their differences; instead, our methods determined ~91% as an optimal allele frequency cut-off for distinguishing sub/lineages, i.e., a sub/lineage-defining site was called if a sub/lineage's major nucleotide differed from at least one other sub/lineage, and had a frequency of at least ~91% in that sub/lineage. (If a single sub/lineage differed from others but its variant was present at only, say, 80% frequency, it was not included.) Thus these sites can harbor genetic variation within sub/lineages, with up to ~9% being an alternative allele. Even given this relatively strict criterion, sub/lineage-defining sites still had more within-sub/lineage variation than the remainder of the genome. This is easily explained if the sites that have been most variable throughout HPV16 evolution continue to be the most variable in the present, because they experience elevated mutation, relaxed purifying selection, positive selection, or a combination of all these forces.

(4) The genetic differences between A3 and A1/2 variants are not particularly large, and the presence of A3 sublineage is ambiguous, as evidenced by the very low number of A3 variants in the PaP and SUCCEED cohorts. Therefore, including the A3 variants separately in the analysis may not be particularly meaningful.

RESPONSE: *We agree that the A3 sublineage is small and poorly characterized. However, previous studies included this sublineage and none of our conclusions depend on A3 alone. Thus, despite its limited value, we ultimately felt A3 was useful to retain for completeness, and because it represents a phylogenetically independent test of positive selection at several codons (Fig. 4a).*

(5) Do the E1 residues (38, 78, 100, 208, 219, 220, 294, 597) that are predicted to undergo positive selection play any functional role in the protein? How is the functional change in E1 associated with the development of cervical cancer?

RESPONSE: *To address this important question, we have (1) performed an additional analysis of E1 protein structure and the predicted impact of observed amino acid variants, and (2) incorporated additional studies of E1's function.*

First, these positions are distributed among the E1 protein's N-terminal regulatory (positions 38, 78, 100), DNA-binding (208, 219, 220, 294), and helicase (597) domains. Position 100 is located specifically within the nuclear export signal (NES) (UniProt ID: P03114). Second, we used the experimentally determined hexameric E1–DNA complex structure from bovine papillomavirus (BPV) (PDB ID: 7APD) to superimpose the 3D structure of HPV16 E1 monomer (already predicted with AlphaFold2) and identify the HPV16 E1 residues at the interface of the protein-DNA complex. Under the assumption that HPV16 E1 engages DNA in a similar binding mode and interaction geometry as that of BPV, residues L208, F219, and S220 in the DNA-binding domain were predicted to be in the proximity of the DNA molecule, which suggests they have a role in facilitating DNA binding and unwinding. Of these, residue 208 was closest in terms of its distance from the interacting DNA molecule, highlighting its significance.

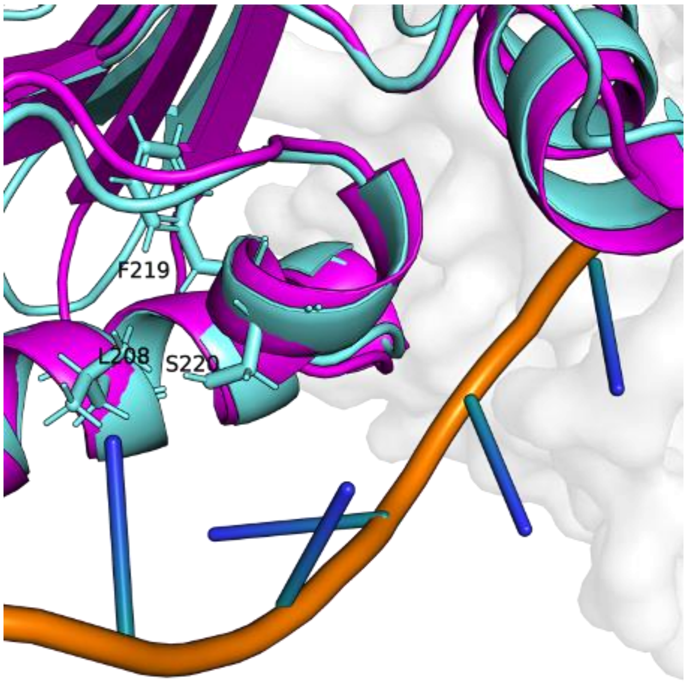

Third, we used an orthogonal approach to evaluate the effect on E1 stability of all amino acid changes observed in our dataset. To do this, we used DynaMut2 (Rodrigues et al., 2021, Protein Sci. 30, 60–69) and DUET (Pandurangan and Blundell, 2020, Protein Sci. 29, 247–257), which report the effect of amino acid changes on the thermodynamic stability of a protein as ΔΔG, the difference in Gibbs free energies of the mutant and wildtype proteins (negative values indicate a destabilizing change, positive values indicate a stabilizing change). We considered |ΔΔG| > 1 to be functionally impactful, following Capriotti et al., 2008 (BMC Bioinformatics 9:S6). By this criterion, amino acid changes observed at positions 100, 208, and 294 were all predicted to be functionally impactful (destabilizing) by at least one method. Remarkably, the mutations present at position 208 (L208Q, L208V), in the proximity of the DNA molecule, also had the most destabilizing effects on E1, suggesting this residue's changes are particularly impactful.

*Structural stability is only one aspect of function. Position 294 (DNA-binding domain) is also present in experimentally-verified epitopes of HLA-A*02:01 and -A*68:01 (IEDB IDs: 111459 and 769595). Providing critical context, literature suggests that (in addition to E6 and E7) E1 transcript levels are positively associated with progression to CIN3+: see Schmitt et al., 2011 (Molecular and Cellular Probes 25:260–265) and Baedyananda et al., 2018 (Intervirology 60:171–180). At the same time, some evidence suggests that while E1 is necessary for initial establishment and productive replication of the HPV16 genome, it may not be necessary for maintenance (Egawa et al., 2012, J Virol 86:3276–3283). Thus, selective constraint could be at least partially relaxed on the E1 ORF during persistence and progression.*

The above has important implications and connects our positively selected E1 residues to potential functional changes and the development of cervical cancer: these mutations could lead to 1) the modification of E1, which could contribute to viral persistence in the basal cell reservoir; or 2) a failure

to control E1 nuclear localization levels, namely because E1 induces the DNA damage response, dysregulated nuclear levels (e.g., via mutation in the N-terminal regulatory domain, especially the aforementioned NES) could lead to genomic instability, integration, and other changes promoting carcinogenesis (noted by Bergvall et al., 2013, Virology 445:35–56).

Finally, since our initial submission, evidence was presented at the 2025 Conference of the International Papillomavirus Society (IPVS 2025) that E1 CTL epitopes induce the strongest CTL responses of any HPV16 protein, both in terms of magnitude as well as breadth (i.e., more epitopes) (Gayton et al., 2025, "Leveraging Computational AI-based Tools to Design a Rational Therapeutic T cell-based Vaccine for HPV", IPVS 2025 conference presentation). This is consistent with E1 having a case/control difference at positively selected codons, particularly if the strongest selective pressures are due to antigen-specific immunity.

To incorporate the most important of the above points, we have made several additions to the main text. To the Results, we have added the following (lines 212–218): "To explore the possible functional roles of these E1 positions, we superimposed our AlphaFold2-predicted monomeric structure of the HPV16 E1 protein onto the experimentally determined hexameric E1–DNA complex from bovine papillomavirus (PDB ID: 7APD[45]). Positions 208, 219, and 220 were located in the proximity of the DNA molecule, suggesting roles in DNA binding and unwinding (Suppl. Fig. S7). Of these, position 208 was closest to the DNA, and its variants (L208Q, L208V) were predicted to destabilize the E1 protein structure ($\Delta\Delta G < -1.0$, DUET[46] and DynaMut2[47]s; Suppl. Table S6)".

*To the Discussion, we have added the following (lines 329–342): "These E1 variants could contribute to persistence and/or progression: E1, E6, and E7 transcript levels are all associated with progression[58,59], and because E1 induces DNA damage, failure to control its nuclear levels (e.g., due to a mutation at position 100 in the nuclear export signal) could lead to genomic instability, integration, or other changes promoting carcinogenesis[60]. In the E1 DNA-binding domain, positions 208, 219, and 220 were predicted to be close to the interacting DNA molecule, with variants at 208 predicted to destabilize the protein. Furthermore, position 294 is located in experimentally verified epitopes restricted by HLA-A*02:01 and -A*68:01 (IEDB IDs: 111459 and 769595), and recent evidence from A*02 knock-in mice suggests E1 epitopes induce the strongest CTL responses of any HPV16 protein in terms of both magnitude and breadth (Gayton et al., 2025, "Leveraging Computational AI-based Tools to Design a Rational Therapeutic T cell-based Vaccine for HPV", International Papillomavirus Society conference presentation). This evidence is consistent with E1 having a case/control difference at positively selected codons, particularly if the strongest selective pressures are due to antigen-specific immunity".*

Finally, to the Methods, we have added the following (lines 575–581): "The predicted HPV16 E1 structure was superimposed onto the bovine papillomavirus E1 structure (PDB ID: 7APD, chain H) using the cealign[88] command in PyMOL (The PyMOL Molecular Graphics System, Version 3.0 Schrödinger, LLC). DUET[46] and DynaMut2[47] were then used to predict the effects of observed amino acid variants on the protein's thermodynamic stability using the statistic $\Delta\Delta G$, the difference in Gibbs free energies of the mutant and wildtype proteins (kcal/mol; negative = destabilizing, positive = stabilizing). Following Capriotti et al.[89], we considered $|\Delta\Delta G| > 1$ to be functionally impactful".

(6) Regarding HLA haplotype, what is the frequency of B*07:02 within the general population in the US? Does that fit with the authors' scenario of virus escape from the host adaptive immunity?

> ***RESPONSE:*** *The frequency of HLA-B*07:02 is an interesting and important question, because HPV16 would be expected to evolve most strongly in response to alleles that are present at appreciable frequencies. To address this, we examined HLA data from the U.S. National Marrow Donor Program (NMDP) database (Gragert et al., 2013, new ref. 37). B*07:02 is indeed among the most common HLA-B alleles worldwide, including the United States. Using the NMDP population stratifications, B*07:02 is the single most common HLA-B allele in European Caucasians (EURCAU, 13.1%), Caucasians as a whole (CAU, 12.5%), North American Indians (AMIND, 10.1%), American Indians from Central or South America (AISC, 9.6%), and Native Americans as a whole (9.7%). Given its high frequency, HLA-B*07:02 would be among the most likely of the thousands of HLA-B alleles to exhibit strong selection pressure on human viruses, and HPV16 would have encountered this allele very frequently in virtually all populations. This is consistent with our scenario of virus escape from the host adaptive immunity and this allele exerting detectable selection on the virus.*
>
> *To provide this background information in the manuscript itself, we have added the following to the Introduction (lines 98–99): "with B*07:02 being among the most high-frequency alleles at the HLA-B locus[37]". We have also added the following to the Discussion (lines 354–356): "Consistent with this, B*07:02 is among the most common of the thousands of HLA-B alleles[37], suggesting HPV16 would have frequently encountered this allele and experienced immune selection."*

Minor issues:

(1) Line 52: cervical cancer is the fourth most common cancer among women worldwide, not the third. Please check a recent WHO estimate: https://www.who.int/news-room/fact-sheets/detail/cervical-cancer

> ***RESPONSE:*** *We thank the reviewer for catching this outdated statistic. We have modified the sentence as follows, using the suggested reference: "remains the fourth most common cancer in women worldwide[1]".*

(2) Line 143: "These results support previous observations that the average HPV16 codon is under purifying selection." The reference papers should be cited here.

> ***RESPONSE:*** *We have added citations to Chen et al. 2005, Hughes and Hughes 2005, and Burk et al. 2023.*
>
> *We thank the reviewer once again for the opportunity to clarify these important issues and improve our manuscript.*

We thank the reviewers for the additional comments, which we have implemented and provide point-by-point responses to below.

REVIEWERS' COMMENTS

Reviewer #1 (Remarks to the Author):

The authors have addressed most of my concerns and substantially improved the manuscript by adding additional analyses and supplementary information.

I have only a few minor suggestions for clarification. First, the authors state that one HPV16-positive sample per woman was analyzed and that outcomes were determined using the full follow-up period. It would be helpful to specify which time point the sequenced HPV16 genome corresponds to (e.g., baseline sample, sample closest to diagnosis, or another defined time point) and to clarify how viral clearance was defined (e.g., a single negative HPV test versus consecutive negatives).

> ***RESPONSE:*** *For our prospective PaP cohort, the first available HPV16-positive sample (i.e., baseline sample) was evaluated. Women in this cohort were undergoing routine cervical cancer screening at Kaiser Permanente and had multiple visits; for an incident infection during the study, the baseline sample would be the first HPV16+ sample, and for a prevalent infection it would be the baseline sample as she entered the study already positive for HPV16.*
>
> *Clearance was defined as at least one HPV16-negative test following an HPV16-positive test and with no subsequent HPV16-positive tests. Of note, clearance was not part of the criteria to select the controls; however, the majority of the controls in our PaP cohort did subsequently clear their infections. The controls were defined as women from the PaP cohort with baseline enrollment specimens that were HPV16-positive with no histologic evidence of CIN2 or worse at enrollment or during the follow-up study period, according to the electronic health records (402 with ≤CIN1 histology and 448 with normal or no histology during our study, i.e., biopsy not indicated).*
>
> *To clarify this, we have added the following to the Methods: "One sample per woman was analyzed, and the first available baseline HPV16-positive sample was used for sequencing" (lines 446–448).*

Second, given that approximately half of the control group includes women with CIN1, it would be useful to indicate whether the key analyses are robust when restricted to lesion-free controls (normal cytology/histology), or to briefly comment on the potential impact of including CIN1 samples in the control group.

> ***RESPONSE:*** *We randomly selected HPV16-positive samples with no histologic evidence of CIN2 or worse at enrollment or during the follow-up study period to best reflect 'controls' in the general population, so this included normal lesion-free controls and those with morphologic features of HPV infection or with equivocal cytologic changes (LSIL, ASC-US) or low-grade CIN (CIN1). We think including all these controls is more conservative actually, as the potential impact of inclusion would be expected to favor the null hypothesis of no effect. Thus, we expect our conclusions to be robust to inclusion of CIN1. In support of this, the one control with rare variation in E1 positively selected codons (PAP228214 in Suppl. Fig. S6) is CIN1/LSIL, so our case/control difference would become even more pronounced if we excluded this group.*

Third, the association involving rare nonsynonymous variants in E1 appears to be driven by a very small number of samples, resulting in wide confidence intervals. A brief discussion of the uncertainty associated with these rare variants, including the possibility that they may represent passenger mutations arising during prolonged infection rather than drivers of persistence or progression, would help contextualize this finding.

> ***RESPONSE:*** *We have added the following sentence to this Discussion: "This rare variation occurs in 11 of 854 cases with A1/A2-sublineage virus and it is possible they could represent passenger mutations arising during prolonged infection rather than drivers of persistence or progression" (lines 319–322).*
>
> *Finally, we have modified the sentence after the previous addition to clarify the significance of the finding: "However, given our previous observation that such rare viral variation is overall more common in controls[10], positively selected positions are atypical in that they instead have more rare amino acid variation in precancers/cancers" (lines 322–324).*

Finally, given the strong phylogenetic structure of HPV16 populations, it would be helpful if the authors could comment on whether the rare E1 variants observed in cases cluster within specific viral subclades. This would help clarify whether the observed association reflects independent occurrences or variation linked to underlying phylogenetic structure.

> ***RESPONSE:*** *This is an important question, because if these variants were present in only one viral subclade, having arisen only once in evolution, they could not be considered independent events. This is addressed directly by our current Suppl. Figure S6 ("Rare variation in HPV16 A1/A2 at positively selected positions in E1"), which shows that rare variants at the E1 positively selected sites arose independently at least six times in evolution. Thus, they do not constitute a single haplotype or related cluster of sequences. To clarify this in the main text, we have added the following to the Results: "These E1 variants do not cluster in a single viral subclade or haplotype, but instead arose independently several times over the course of viral evolution (Suppl. Fig. S6)" (lines 210–212).*

Overall, the manuscript has been substantially improved after revision, and I believe the remaining points can be addressed with minor clarification. The study will likely be of interest to readers studying HPV evolution and host–virus interactions.

> ***RESPONSE:*** *We thank the reviewer for the positive assessment and comments that have improved our study.*

Reviewer #2 (Remarks to the Author):

The authors addressed all my comments and the manuscript was significantly improved. Thank you for giving me the opportunity to review this important paper.

> ***RESPONSE:*** *We thank the reviewer for the positive assessment and ideas to improve our study.*
